# Click'n ROPISA: One‐Pot Synthesis of Ultrabright Fluorescent Nanoparticles Made of Polypeptides

**DOI:** 10.1002/anie.1373955

**Published:** 2026-07-15

**Authors:** Amin El Jarroudi Hammou, Juliette Lavainne, Rohit Mangalwedhekar, Jyothis Devasia, Laurent Cognet, Sébastien Lecommandoux, Colin Bonduelle, Chloé Grazon

**Affiliations:** ^1^ University of Bordeaux CNRS Bordeaux INP ISM UMR 5255 Talence France; ^2^ University of Bordeaux CNRS Bordeaux INP LCPO UMR 5629 Pessac France; ^3^ Laboratoire Photonique Numérique et Nanosciences University of Bordeaux Talence France; ^4^ LP2N, Institut d'Optique Graduate School, CNRS – UMR 5298 Talence France; ^5^ Institut Universitaire de France Paris France

**Keywords:** anisotropy, CuAAC, FRET, microscopy, *N*‐carboxyanhydride, PISA

## Abstract

Fluorescent nanoparticles are widely used in bioimaging and sensing, yet their preparation relies on multistep synthetic routes that limit scalability and structural control. Here we introduce a one‐pot strategy that couples aqueous ring‐opening polymerization‐induced self‐assembly (ROPISA) of γ‐propargyl‐L‐glutamate *N*‐carboxyanhydride with copper‐catalyzed azide–alkyne cycloaddition (CuAAC) to produce fluorescent polypeptide nanoparticles. This one‐pot “Click'n ROPISA” process enables the simultaneous formation of polypeptide nano‐objects and covalent incorporation of diverse azido‐functionalized fluorophores. The resulting assemblies exhibit tunable emission across the visible spectrum and are accessible at high solids content while maintaining high brightness and structural uniformity. Dense fluorophore incorporation enables efficient Förster resonance energy transfer (FRET) both within the assemblies and with dye‐labeled proteins, supporting sensing applications. The intense brightness of the red‐emitting nanoparticles further enables single‐particle tracking in microscopy toward advanced bioimaging.

## Graphical abstract

Self‐assembly and fluorophore conjugation proceed rapidly in a one‐pot “Click'n ROPISA” process, yielding intensely fluorescent polypeptide nanoparticles with tunable visible emission. High dye loading enables efficient energy transfer and single‐particle visualization, while the straightforward synthesis provides versatile access to bright nanomaterials for sensing and bioimaging.

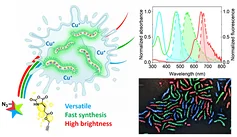

## 1 Introduction

Self‐assembly plays a central role in nanofabrication, enabling the production of nanomaterials with applications in fields ranging from biomedical engineering to electronics and fluorescence spectroscopy [1, 2, 3, 4, 5]. In particular, luminescent nanoparticles offer an attractive alternative to small organic dyes in modern bioimaging, which relies heavily on fluorescence‐based techniques. Although molecular dyes remain the most widely used probes because of their small size and accessibility, their limited brightness prevents high image resolution in microscopy and the detection of analytes at sub‐nanomolar concentrations [6, 7, 8]. In contrast, luminescent nanoparticles (NPs) offer enhanced brightness and improved photostability [7, 9, 10]. However, leading systems such as quantum dots (QDs) [11] and up‐conversion NPs [12] are typically based on non‐degradable matrices, require multistep fabrication, and raise concerns regarding long‐term environmental persistence. Organic dye‐doped polymer NPs are an emerging alternative [13, 14]. In their simplest implementation, they are obtained via nanoprecipitation of a pre‐synthesized amphiphilic polymer together with an organic dye encapsulated in the hydrophobic core [15, 16]. However, the process requires multiple preparation steps and is limited to relatively low concentrations of nanoparticles (0.001–0.02 wt% [17, 18]). Additionally, dye leakage from the hydrophobic core occurs when the fluorophores are not specifically modified or optimized for encapsulation [19].

Polymerization‐induced self‐assembly (PISA) has emerged as a powerful method that enables the simultaneous polymerization and self‐assembly of amphiphilic block copolymers in a single step and at high solid contents, leading to NPs with well‐defined and homogeneous architectures [20, 21]. Nevertheless, most PISA rely on controlled radical polymerization, yielding to non or only partially‐degradable vinyl‐based cores, while fully degradable systems remain comparatively underdeveloped [22, 23, 24, 25]. In addition, the synthesis of fluorescent PISA‐derived nanoparticles is generally carried out in mixed organic solvent systems containing water [26, 27]. Furthermore, to the best of our knowledge, the direct incorporation of fluorophores into purely aqueous PISA processes has not yet been achieved. Beyond the limited water solubility of many organic dyes, this limitation likely arises from their sensitivity to radical conditions, particularly for red‐emitting fluorophores, as well as from the incompatibility of reactive groups (e.g., maleimides, primary amines, phenols), with radical polymerization processes, especially alkyne/azido groups involved in click‐type reactions such as the Meldal‐Sharpless copper‐catalyzed azide–alkyne cycloaddition (CuAAC) [28, 29].

Herein, we overcome all these limitations through a simple one‐pot process performed entirely in water: the aqueous ring‐opening polymerization‐induced self‐assembly (ROPISA) of *N*‐carboxyanhydrides (NCA) [30]. In contrast to radical‐mediated PISA approaches, we demonstrate that this system is fully compatible with CuAAC [31]. Using propargyl‐functionalized L‐glutamate *N*‐carboxyanhydride (PLG‐NCA) [32, 33, 34] and azido‐dyes, the polymerization and click functionalization are performed in a rapid single‐step process termed ROPISA‐CuAAC (Scheme 1). This strategy affords fluorescent nanoparticles with unprecedented tunable emission across the visible spectrum (from blue‐emitting coumarins to red‐emitting cyanine derivatives) including particularly bright cyanine‐based NPs. The ROPISA‐CuAAC approach accommodates fluorophores spanning a broad range of polarity, charge, and molecular weight, underscoring its generality. In addition, we demonstrate that this novel class of nanoparticles can mediate efficient Förster resonance energy transfer (FRET) [35], both internally and with dye‐labeled proteins. The intense brightness of the cyanine nanoparticles further enables their individual localization and tracking using super‐resolution imaging techniques. Collectively, these results establish ROPISA‐CuAAC as a versatile platform for the one‐step synthesis of ultra‐bright, degradable, and addressable fluorescent nanoparticles suitable for dynamic imaging and biosensing applications.

**SCHEME 1:**
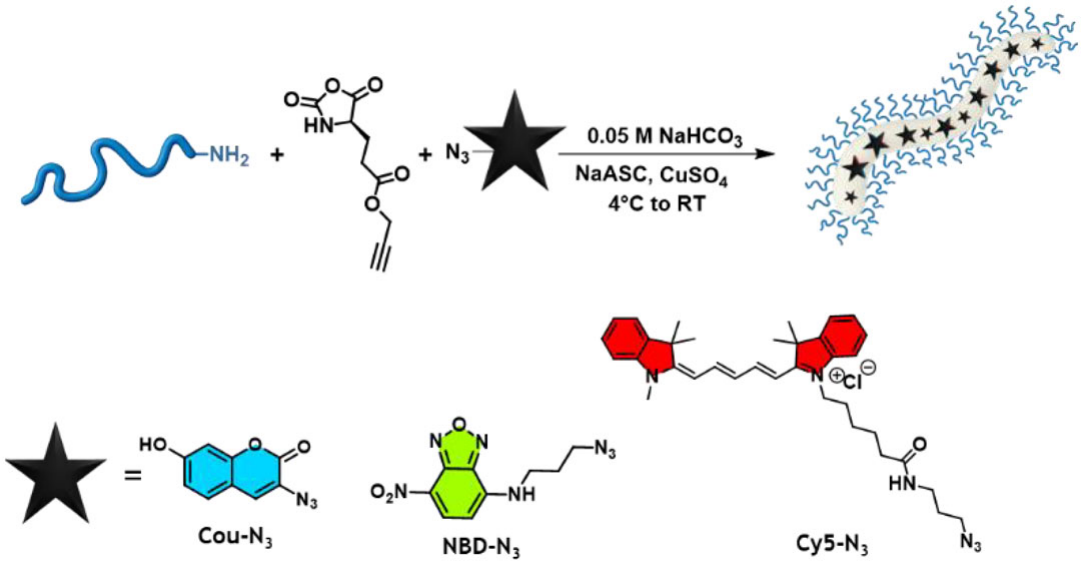
One‐step synthesis of fluorescent nanoparticles in water by ring opening polymerization induced by self‐assembly (ROPISA) combined with copper‐assisted azide alkyne cycloaddition (CuAAC): PEG‐NH_2_ initiates the polymerization of PLG‐NCA and concomitantly an azido‐dye (3‐azido‐7‐hydroxycoumarin (Cou‐N_3_), N‐(3‐azidopropyl)‐7‐nitro‐2,1,3‐benzoxadiazol‐4‐amine (NBD‐N_3_) and/or cyanine 5‐azide (Cy5‐N_3_) is grafted on the polymer backbone.

## 2 Results and Discussion

### 2.1 ROPISA

We first investigated the ability of γ‐propargyl L‐glutamate NCA (PLG‐NCA) to undergo polymerization under ROPISA conditions [30]. This monomer presents a unique case, as it is a viscous liquid at room temperature, in contrast to the solid NCAs typically employed in previously reported ROPISA [36]. The synthesis of PLG‐NCA is already described in the literature using different methods [32, 34, 37]. However, in this work, the synthesis was carried out using a phosgene‐free method [38], affording the monomer with a 49% molar yield after 3 preparation steps (Scheme S1, Figures S1–S3). For the ROPISA process, the macroinitiator poly(ethylene glycol)‐NH_2_ (PEG‐NH_2_, *M_n_* = 5000 g.mol^−1^) was dissolved in bicarbonate buffer (0.05 M, pH 8.5) and introduced to PLG‐NCA ([M]/[I] = 20) in a plastic microtube (Scheme S4). Efficient dispersion of the liquid monomer was achieved by brief vortexing followed by sonication in an ice‐cooled bath. A yellowish dispersion was obtained after 10 min. The crude product was purified by dialysis against ultrapure water for 2 days (Figure S9). Size exclusion chromatography (SEC) analysis revealed a leftward shift of the elution peak compared to the PEG initiator, confirming the successful formation of the block copolymer PEG‐*b*‐PPLG (Figure 1a). Following polymerization, the resulting nanoparticles were dialyzed and characterized by transmission electron microscopy (TEM, Figure 1c). Elongated objects with average dimensions of approximately 300 nm in length and 10 nm in width were observed. This finding confirms that, even when employing a liquid monomer, ROPISA exclusively yields elongated nanoparticles, a feature attributed to the intrinsic propensity of the polypeptide chains to adopt β‐sheet secondary structures during self‐assembly [36, 39, 40].

**FIGURE 1:**
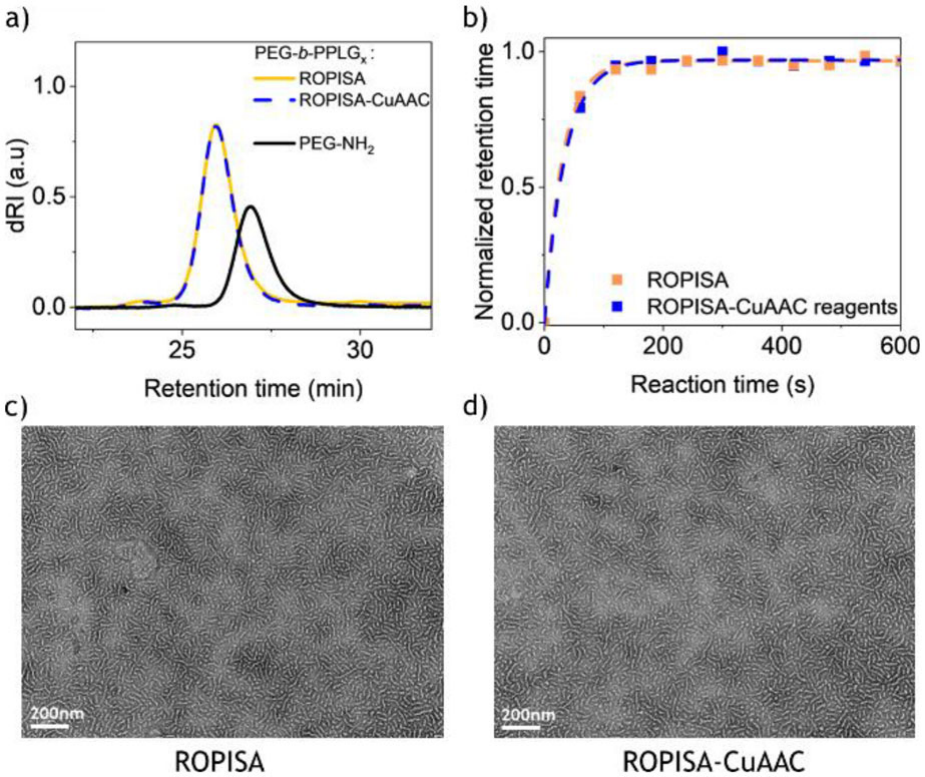
PEG_113_‐*b‐*PPLG_x_ NPs synthesized by ROPISA or in the ROPISA‐CuAAC conditions. (a) Size‐exclusion chromatograms of the polymers in DMF (+LiBr 1 mg.mL^−1^). (b) Comparison of the normalized retention time (SEC) in function of the reaction time. (c–d) TEM image of the NPs (scale bar = 200 nm, stained using uranyl acetate). (c) ROPISA. (d) ROPISA‐CuAAC.

### 2.2 ROPISA‐CuAAC

A key methodology for introducing a fluorophore is the use of Meldal‐Sharpless cycloaddition, a reaction that can be limited when carried out concomitantly with radical polymerization [28, 29]. We anticipate that our alternative strategy, will unlock new opportunities in polymer synthesis and functional nanoparticle design. To first evaluate the potential interference of copper‐based catalysts, the polymerization was conducted in the presence of copper and sodium ascorbate, hereafter referred as ROPISA‐CuAAC. Remarkably, the reaction yielded a similar yellowish dispersion, and SEC analysis showed a comparable leftward shift relative to the initiator (Figure 1a). TEM analysis confirmed that the nanoparticles retained the same anisotropic morphology observed under standard ROPISA conditions (Figure 1d). In addition, the kinetic of the formation of the PPLG block during the ROPISA or ROPISA‐CuAAc was monitored by SEC in DMF (Figures S10–S11), following acid quenching [41]. Remarkably, kinetic monitoring revealed that the reaction reached completion within only 2 min (Figures 1b) with no detectable influence of copper on the polymerization rate. While sonication is known to accelerate NCA polymerization rates [42], the process reported here proceeds very rapidly while maintaining excellent control over the polymer chains (*M_n_* = 8400 g.mol^−1^, *Ð* = 1.04). Overall, these results confirm the formation of the PEG‐*b*‐PPLG copolymer, indicating that CuAAC reagents did not interfere with the polymerization.

### 2.3 Clicking Fluorophores Using ROPISA‐CuAAC

Since ROPISA was found to be compatible with copper and ascorbate, we decided to incorporate a fluorophore into the ROPISA process to promote polypeptide grafting. The same protocol as described above was used, except that 3‐azido‐7‐hydroxycoumarin (Cou‐N_3_, 5% mol relative to PLG‐NCA) was introduced at the beginning of the polymerization together with the other reactants (Scheme S5). After the synthesis, the dispersion was dialyzed to remove copper and other salts. Elemental analysis confirmed the complete removal of copper during purification, with residual copper levels below the detection limit (see Supporting Information for details). SEC confirmed the formation of polymers with controlled molar mass (Figure 2a, *M_n_* = 7700 g.mol^−1^, *Đ* = 1.14) and the effective incorporation of the dye into the polymer backbone (with the absorbance detector set to the characteristic wavelength of coumarin; Figure S12). In addition, ^1^H NMR and absorption analysis of the purified samples allowed determination of the effective dye % in the final polymer chains (Equation S5, Table 1, Figure S13). The experimental dye % is close to the theoretical value, confirming the efficiency of the synthesis. TEM and cryo‐TEM images (Figure 2b–d) revealed elongated objects, once again, although a small fraction of spherical species was also observed in this case. The resulting nanoparticles absorbs at 350 nm and emit at 475 nm in water (Figure 2c), with a fluorescence quantum yield of 6% associated with a lifetime of 3.3 ns (Table 1). Moreover, the brightness per volume was determined using Equation S6, leading to a value of 160 M^−1 ^cm^−1 ^nm^−3^, that is, in the same order of magnitude as BlueCy‐doped in polymethacrylates NPs [43].

**FIGURE 2:**
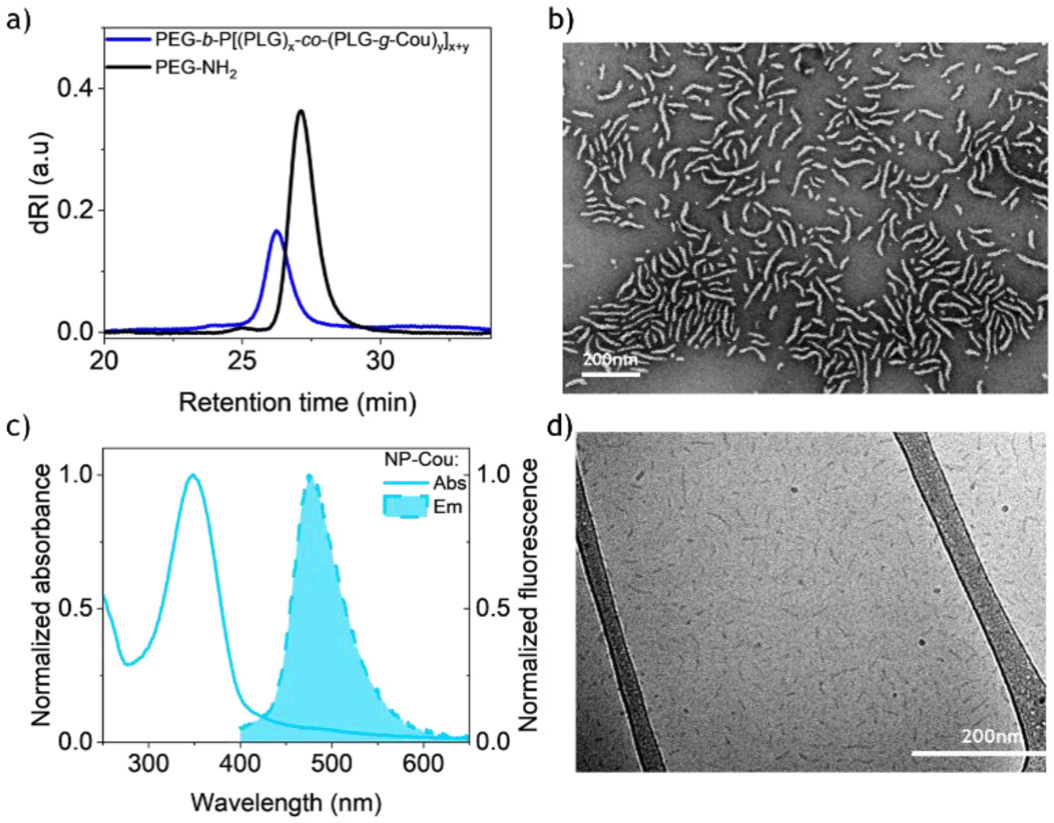
PEG_113_‐*b*‐P[(PLG)_x_‐*co*‐(PLG‐*g*‐Cou)_y_]_x+y_ NPs synthesized by ROPISA‐CuAAC. (a) Size‐exclusion chromatograms of the polymers in DMF (+LiBr 1 mg.mL^−1^) RI detection. (b) TEM image of the NPs (scale bar = 200 nm, stained using uranyl acetate). (c) Absorption and florescence emission spectra (*λ*_exc_ = 350 nm) of the NPs in water. (d) Cryo‐TEM image of the NPs in water (scale bar = 200 nm).

**TABLE 1:** Properties of the copolymers obtained by click'n ROPISA and of the corresponding fluorescent nanoparticles.

		% dye grafted			Theorical		SEC^b^		Spectroscopy				
		Theo.	exp^a^	Exp		*M*_n_^th^	*M*_n_		*λ*_abs_^max^	*λ*_em_^max^	*ϕ*_F_ ^c^	*τ* ^d^	B_v_ × 10^3^
Copolymer	Dye‐N_3_	mol%	mol%	wt%	[*M*]/[*I*]	kg.mol^−1^	kg.mol^−1^	Đ	nm	nm	%	ns	M^−1^ cm^−1^ nm^−3^
PEG_113_‐*b*‐PPLG_x_	—	—	—	—	20	8.4	8.8	1.04	—	—	—	—	—
PEG_113_‐*b*‐P[(PLG)_x_‐*co*‐(PLG‐*g*‐Cou)_y_]_x+y_	Cou‐N_3_	5%	4.8%	6%	20	8.6	7.7	1.14	350	475	6	3.3	0.16
PEG_113_‐*b*‐P[(PLG)_x_‐*co*‐(PLG‐*g*‐NBD)_y_]_x+y_	NBD‐N_3_	5%	5.2%	8%	20	8.7	9.5	1.12	470	550	7	2.6	0.34
PEG_113_‐*b*‐P[(PLG)_x_‐*co*‐(PLG‐*g*‐Cy5)_y_]_x+y_	Cy5‐N_3_	5%	4.4%	18%	20	9	8.4	1.14	650	670	11	0.8	6.7

^a^ Experimental mol% of dye grafted determined by absorption spectroscopy.

^b^ Number‐average molar mass (*M_n_*) and molar‐mass dispersity (*Đ*) determined by SEC in DMF (+1 g.L^−1^ LiBr or 8.7 g.L^−1^), using polystyrene standards for calibration.

^c^ Fluorescence quantum yield (*ϕ*_F_), using the following references: Cou: quinine bisulfate (QBS, *ϕ*_F_ = 0.546 in 0.5 M H_2_SO_4_) [53], NBD: 4‐dicyanomethylene (DCM, *ϕ*_F_ = 0.437 in EtOH) [54], and Cy5: Nile Blue (NB, *ϕ*_F_ = 0.27 in EtOH) [55].

^d^ Amplitude‐weighted average fluorescence lifetime, measured at an excitation wavelength (*λ* ^exc^) and emission wavelength (*λ* ^em^) appropriate for each dye (Cou: *λ* ^exc^ = 370 nm, *λ* ^em^ = 475 nm, NBD: *λ* ^exc^ = 455 nm, *λ* ^em^ = 560 nm, and Cy5: *λ* ^exc^ = 670 nm, *λ* ^em^ = 680 nm)‐based nanoparticles.

^e^ Brightness per volume calculated using Equation (S6).

We then investigated the kinetics of both reactions by monitoring each process independently. Cou‐N_3_ is perfectly suitable for this purpose, as it is a fluorogenic probe that enables real‐time monitoring of triazole formation by fluorescence spectroscopy [44]. As shown in Figure S15, the reaction exhibited a gradual increase in blue fluorescence, plateauing after ∼100 min. Comparative kinetic analysis of the ROPISA polymerization and the CuAAC click reaction was performed (Figure 3), and the kinetic profiles were fitted using a first‐order exponential decay model. The CuAAC reaction displays a low‐rate constant of *k*_obs_ ∼ 1 *×* 10^−3^ s^−1^. Since the click reaction follows a second‐order rate law, from this pseudo‐first‐order rate constant (*k*_obs_), a second‐order rate constant of *k_2_ = k_obs_ ×* [Cou‐N_3_]^−1^ ∼ 0.06 M^−1^.s^−1^can be determined. This *k*_2_ indicates that the CuAAC reaction is slower that the value reported CuAAC kinetics in mixed organic/water for small molecules (*k*_2_ *∼* 10–10^4^ M^−1^.s^−1^) [45, 46, 47, 48, 49], likely because of the limited solubility of reactants in aqueous media and the complexity of the ROPISA system. In opposition, the ROPISA process exhibited a significantly higher rate constant of *k*_1_ = 0.03 s^−1^. This stark contrast highlights the high efficiency of ROPISA, which reaches completion within 2 min, whereas the CuAAC reaction requires ∼100 min under identical conditions. Importantly, although performed sequentially, the CuAAC click reaction does not interfere with the ROPISA process, as evidenced by the preservation of the expected polymerization behavior and particle morphology. This absence of cross‐reactivity demonstrates the true orthogonality and robustness of the system, enabling temporal decoupling of polymerization and post‐functionalization steps. Moreover, the invariance of the self‐assembled structures during the slower CuAAC transformation suggests that the nano‐objects are kinetically trapped on the experimental timescale, allowing for late‐stage functionalization without structural reorganization. Collectively, these results establish a versatile platform for the controlled synthesis and modification of polymer nanostructures.

**FIGURE 3:**
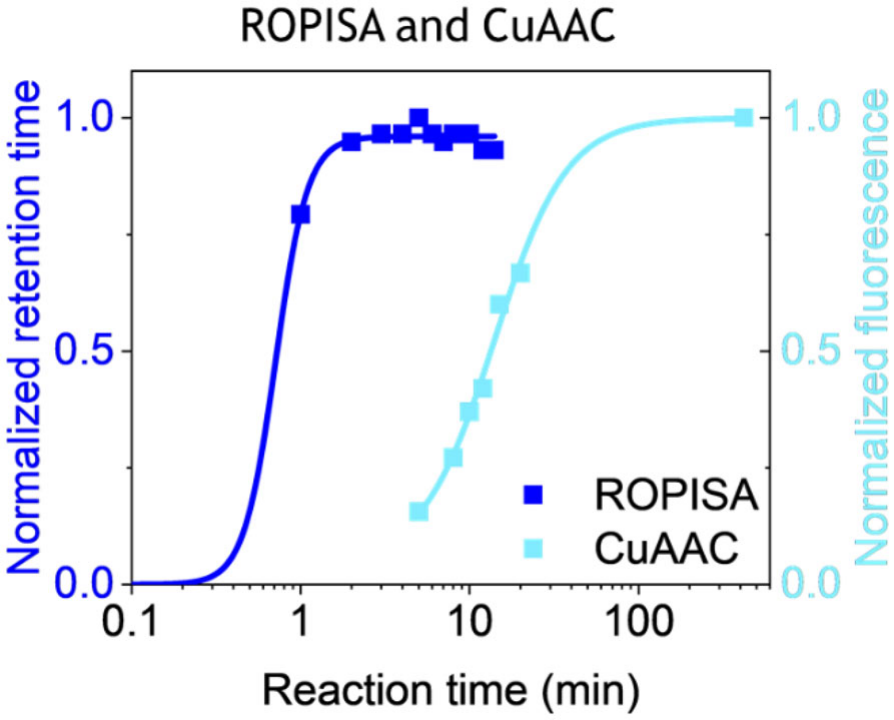
Comparison of the kinetics of the PPLG block formation (analyzed by size exclusion chromatography retention time) and the triazole formation (analyzed by fluorescence using the fluorogenic probe Cou‐N3, see Figure S15). The kinetic profiles were fitted using a first‐order exponential decay model.

### 2.4 Tuning NPs’ Fluorescence

For biological imaging, fluorescent probes emitting in the red or near‐infrared (NIR) are highly advantageous due to deeper light tissue penetration and minimized background autofluorescence. Consequently, conventional blue‐emitting dyes, such as Cou‐N3, are less suitable for in vivo applications. To expand the versatility of our system and enable the development of ratiometric fluorescent sensors, we incorporated multiple clickable dyes with distinct emission profiles: N‐(3‐Azidopropyl)‐7‐nitro‐2,1,3‐benzoxadiazol‐4‐amine (NBD‐N3, green‐emitter, synthesis described in Scheme S2, Figures S4 and S5) and cyanine 5 (Cy5‐N3, red‐emitter). Using NBD‐N3 (5 mol%) (Scheme S6), a yellow dispersion was obtained. SEC in DMF showed a shift to lower retention times, confirming copolymer formation (*M_n_* = 9500 g.mol^−1^, *Đ* = 1.12, Figure 4a). SEC absorption detection confirmed successful NBD incorporation into the polymer backbone (Figure S16). Absorption studies (Table 1) and ^1^H NMR (Figure S17) spectra on the purified copolymers indicate grafting efficiency around 100%. TEM revealed anisotropic nanoparticles (Figure 4b). Spectroscopic analysis indicated that the nanoparticles absorb at 470 nm and emit at 550 nm. Since NBD is a mildly solvatochromic dye, the positions of its absorption and emission bands provide information about its local environment. By comparing the spectra of the nanoparticles in water with the NBD in various solvents, we observed that the emission closely overlaps with that of propargyl alcohol (Figure S18), confirming that the dye is in the hydrophobic PLG environment rather than in the aqueous phase. In addition, the nanoparticles present a quantum yield of 7% and a fluorescence lifetime of 2.6 ns (Figure 4c, Table 1). The brightness per volume was determined using Equation (S6), yielding B_v_ = 340 M^−1^cm^−1^nm^−3^ (Table 1). This value is consistent with literature reports for green‐emitting nanoparticles. For instance, Z. Tian et al. [50] reported twisted perylene‐based nanoparticles emitting at 550 nm with a comparable brightness per volume (B_v_ = 310 M^−1^cm^−1^nm^−3^), confirming that such polypeptidic nanoparticles fall within the same performance range, thought higher brightness values have been reported BODIPY‐based nanoparticles [14].

**FIGURE 4:**
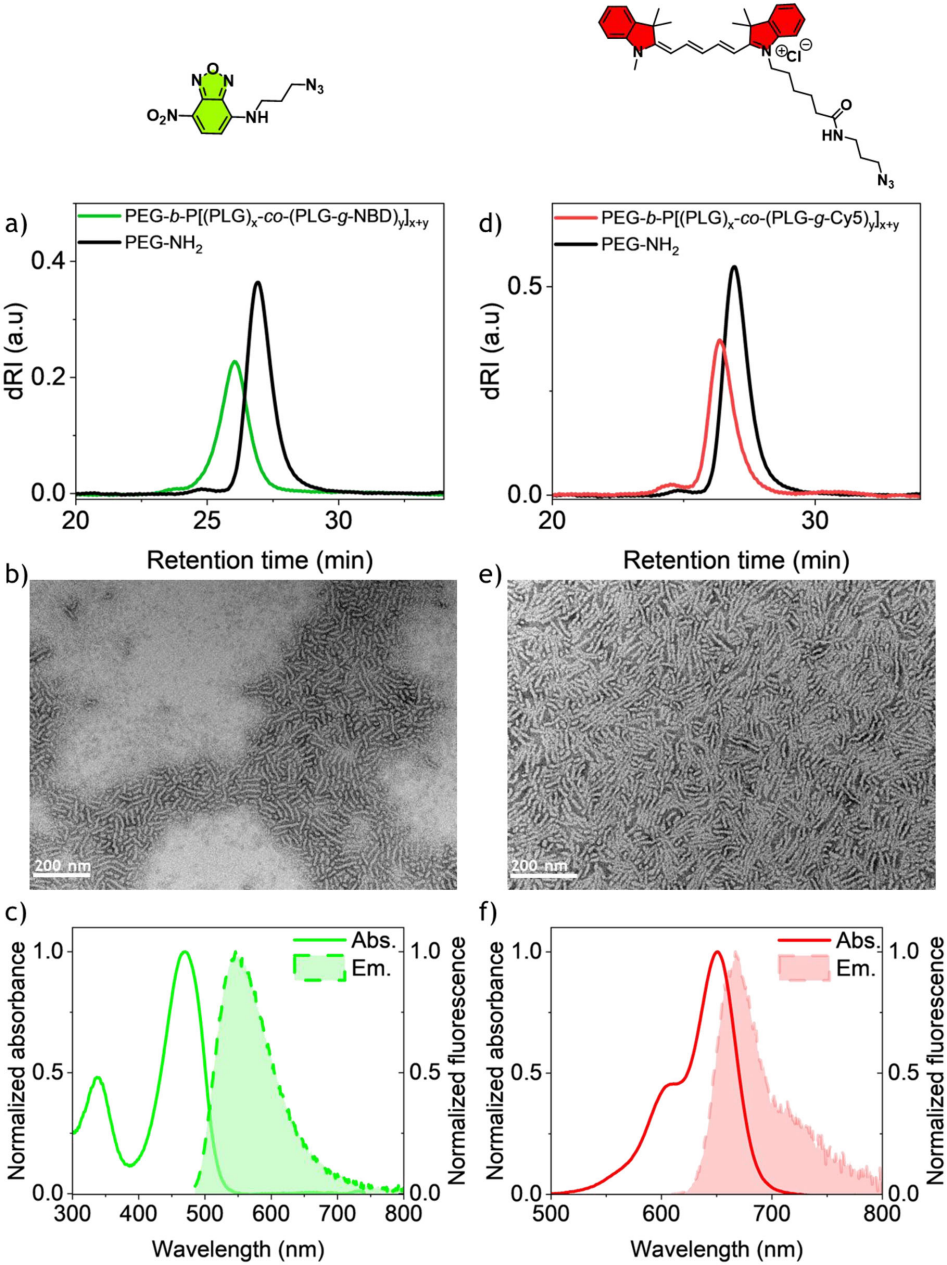
(a–c) PEG_113_‐*b*‐P[(PLG)_x_‐*co*‐(PLG‐*g*‐NBD)_y_]_x+y_ NPs synthesized by ROPISA‐CuAAC. (a) Size‐exclusion chromatograms of the polymers in DMF (+LiBr 8.7 g.L^−1^), RI detection. (b) TEM image of the NPs (scale bar = 200 nm, stained using uranyl acetate) (c) Absorption and florescence emission spectra (*λ* ^exc^ = 450 nm) of the NPs in water. (d–f) PEG_113_‐*b*‐P[(PLG)_x_‐*co*‐(PLG‐*g*‐Cy5)_y_]_x+y_ NPs synthesized by ROPISA‐CuAAC. (d) Size‐exclusion chromatograms of the polymers in DMF (+LiBr 8.7 mg.mL^−1^), RI detection. (e) TEM image of the NPs (scale bar = 200 nm, stained using uranyl acetate). (f) Absorption and florescence emission spectra (*λ* ^exc^ = 630 nm) of the NPs in water.

Finally, for Cy5 (used at 5 mol%) (Scheme S7), the reaction produced a blue dispersion. SEC analysis confirmed copolymer formation with *M_n_* = 8400 g.mol^−1^ and *Đ* = 1.14 (Figure 4d) with successful incorporation of the dye in the polymer chain (Figure S20). Absorption studies (Table 1) and ^1^H NMR (Figure S21) spectra on the purified copolymers indicates grafting efficiency around 90%–100%. Once again, TEM images showed the formation of anisotropic nanoparticles (Figure 4e). Spectroscopic characterizations in water revealed that the nanoparticles absorb at 650 nm and emit at 670 nm, similar to the isolated Cy5, with a quantum yield of 11% and a fluorescence lifetime of 0.8 ns (Figure 4f, Table 1). Such NPs exhibit an exceptional B_v_ of 6700 M^−1^cm^−1^nm^−3^ (Table 1), a value surpassing those reported in the literature for other red‐emitting nanoparticles, especially Cy5‐doped poly(methyl methacrylate) NPs (B_v_ = 4530 M^−1^cm^−1^nm^−3^) [51] or Cy5‐doped in PLGA NPs (B_v_ = 840 M^−1^cm^−1^nm^−3^) [52].

This ability to incorporate molecules of diverse structures, polarities, charges, and molar masses into the PPLG chain highlights the versatility of the ROPISA approach. Importantly, the three types of nanoparticles can be freeze‐dried for storage and subsequently redispersed in water without altering their morphology, demonstrating their practical stability and robustness for later applications (Figures S14, S19, S22, S23).

### 2.5 FRET to Study Supramolecular Copolymer Assemblies (Intra‐Particle FRET)

FRET is widely used as a molecular nanoruler [56], enabling the use of fluorescent probes to report on their local environment. Fluorescence spectroscopy thus affords a direct view of the molecular interactions within a single macromolecule or between different polymer chains [57]. To achieve FRET, a donor (D) and an acceptor (A) pair are required. In this work, two FRET pairs can be selected: Cou/NBD or NBD/Cy5. The Förster radius (R0, Equation S8) of the Cou‐NPs and NBD‐NPs pair is 2.7 nm, and of the NP‐NBD and NP‐Cy5 pair is 4.2 nm. Therefore, the three types of fluorescent nanoparticles obtained here can, in principle, engage in pairwise FRET (D/A: Cou/NBD or D/A: NBD/Cy5). However, because the S0→S_1_ absorption band of coumarin overlaps with the S_0_→S_3_ band of NBD, selective excitation of the donor is not feasible. Accordingly, the NBD–NPs/Cy5‐NPs pair was chosen as the most suitable FRET system. Using this FRET pair, we investigated the dynamics of polymer chain exchange within the nanoparticles. To this end, NBD–NPs and Cy5–NPs were mixed in pure water at C∼0.02 mg.mL^−1^, and the fluorescence emission ratio of acceptor to donor (upon selective excitation of the donor, i.e., NBD) was monitored over time. Samples were incubated at 4, 25, 37, or 50°C for up to 6 days. At 4°C, no spectral changes were detected, whereas at 50°C, a pronounced Cy5 emission band progressively appeared, indicating the apparition of energy transfer (Figure 5). This observation demonstrates that polymer chains from NBD–NPs and Cy5–NPs come into close proximity through nanoparticle mixing and dynamic polymer chain exchanges, with maximum energy transfer reached after 2 days. Similarly, FRET was observed for samples at 25°C and 37°C, with faster onset at higher temperatures. Fitting the experimental data with an exponential function enabled extraction of the characteristic relaxation time (*τ*_r_) and construction of an Arrhenius plot (Figure 5c). Activation energy can be estimated from this plot, that is, temperature dependence of the relaxation time (Equations S11–S13) and is 70 kJ.mol^−1^ for such NPs based on amphiphilic PEG‐b‐PPLG copolymers. To further elucidate this dynamic character, the glass transition temperature (*T*_g_) of the PPLG hydrophobic core (Obtained by ROP in anhydrous conditions, Scheme S3, Figure S6, Figure S7) was determined by DSC analysis, yielding a value of approximately 16°C (Figure S8), which is in good agreement with the literature value of ∼20°C [58], consistent with the observed absence of exchange at 4°C and the progressively faster dynamics at 25°C–50°C. Without entering into detailed mechanistic studies, our findings suggest that the polypeptidic nanoparticles are dynamic, with exchange processes slower than in surfactant micelles but faster than in frozen‐core micelles made of block copolymers with high *T*_g_ [59]. By contrast, when the nanoparticles are mixed using ultrasonication, energy transfer occurs almost instantaneously and nearly reaches its maximum intensity (*E* ∼ 70%, Figure S28d). This procedure enables the rapid formation of dual‐colored NPs. Of note, the sonicated nanoparticles retain the same morphology as the original ones (Figure S23).

**FIGURE 5:**
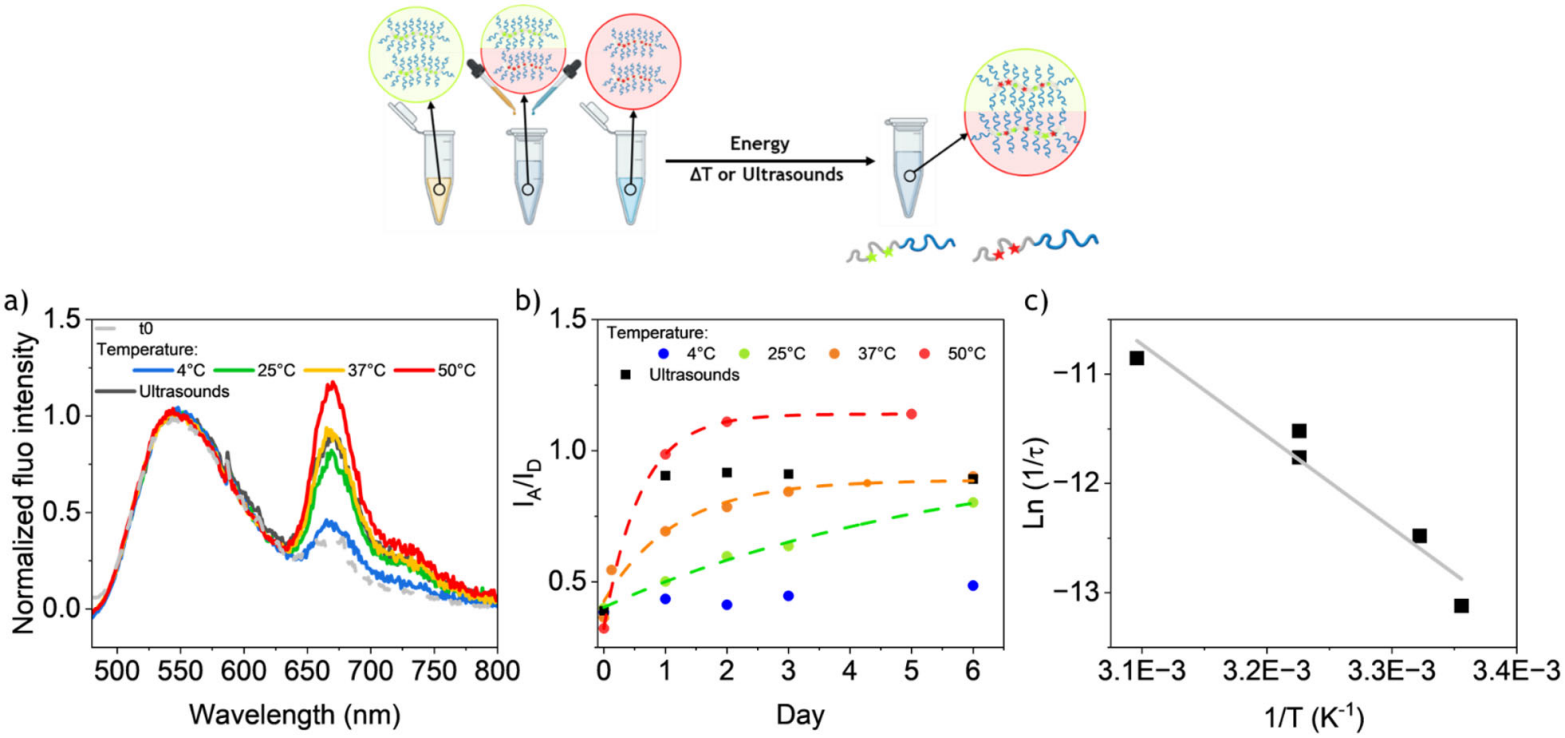
FRET to study chains exchange dynamics between donors PEG_113_‐*b*‐P[(PLG)_x_‐*co*‐(PLG‐*g*‐NBD)_y_]_x+y_ (D) and acceptors PEG_113_‐*b*‐P[(PLG)_x_‐*co*‐(PLG‐*g*‐Cy5)_y_]_x+y_ (A) in pure water at C∼0.02 mg.mL^−1^. (a) Normalized fluorescence emission spectra of the donors and acceptors NPs in water, after reaching equilibrium (*λ* ^exc^ = 470 nm). (b) Fluorescence intensity (*I*_A_@*λ* ^em^ = 670 nm and *I*_D_@*λ* ^em^ = 550 nm) ratio of the acceptor and donor over time at different temperatures or after sonication (45°C). Experimental values (points) are fitted with Equation S13 (dotted lines). (c) Arrhenius plot.

Finally, to confirm that our observations stemmed from chain exchange rather than non‐specific interparticle interactions, we gradually titrated Cy5‐labeled nanoparticles (Cy5‐NPs) into NBD‐labeled nanoparticles (NBD‐NPs) and monitored the fluorescence quenching of NBD‐NPs as a function of concentration (Stern‐Volmer analysis, Figure S28c, Equation S10; data obtained after the deconvoluted fluorescence spectra, Figures S25–S27). The linear quenching profile obtained under static conditions indicated the occurrence of collisional quenching between nanoparticles, albeit minimal at working concentrations. By contrast, when the same experiment was performed under sonication, a bell‐shaped quenching curve emerged, providing definitive evidence for the specificity of chain‐exchange interactions.

### 2.6 FRET to Study NP‐Ligand Binding (Core‐Shell FRET)

To demonstrate the potential of these nanoparticles as FRET‐based biosensors or biomarkers, we engineered biotin‐functionalized core‐shell nanoparticles (BtNBD‐NPs). In this system, 10% of the macroinitiator consist of biotin‐PEG‐NH_2_, while the remaining 90% is conventional PEG‐NH_2_. Synthesized under optimized ROPISA‐CuAAC conditions using NBD‐N_3_, these nanoparticles formed a stable yellow/orange dispersion. SEC analysis in DMF confirmed the successful formation of the copolymer, as evidenced by a clear shift to higher molar masses compared to the initiator (Figure S24a). TEM imaging further revealed the formation of anisotropic nanoparticles (Figure S24b), consistent with our design.

To validate the biotin–streptavidin recognition capability, we performed a FRET binding assay using Texas Red–labeled streptavidin (TR‐SA) as the acceptor (*R*_0_ = 4.0 nm). Given the exceptionally high affinity of the biotin–streptavidin interaction (*K*_d_ ∼10^−14^ M), we anticipated robust FRET upon complex formation. Fluorescence spectroscopy confirmed this hypothesis: gradual addition of TR‐SA to BtNBD‐NPs resulted in quenching of the NBD emission band at 550 nm, accompanied by a concomitant increase in TR‐SA emission at *λ* ^em^ = 615 nm (Figures 6a, S29, and S30). This spectral shift is characteristic of FRET, demonstrating efficient energy transfer (*E* ∼ 20%) (Figure 6b) between the NBD‐NPs and TR‐SA. Control experiments with biotin‐free NBD‐NPs showed no quenching (Figure S30), further confirming the specificity of the interaction. Such FRET efficiencies are of the same order of magnitude as those reported for dye‐labeled protein‐coated with quantum dots [8] or pdots [60], making it possible to envision similar types of FRET‐based biosensing applications for these original nanostructures.

**FIGURE 6:**
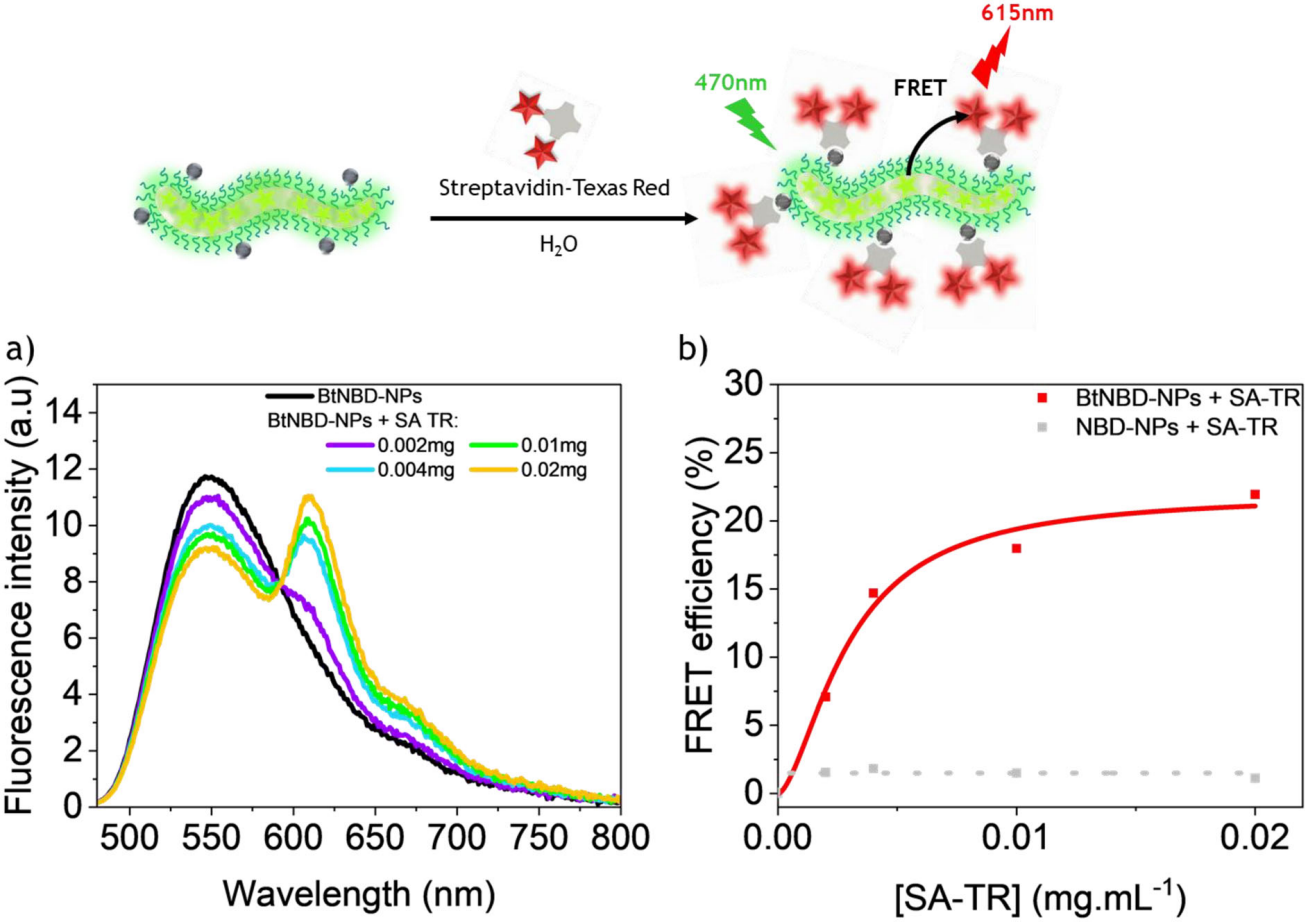
(a) Fluorescence intensity of BtNBD‐NPs excited at *λ* ^exc^ = 470 nm upon addition of gradual concentrations of TR‐SA. (b) FRET efficiency (*E*) of NBD‐NPs and BtNBD‐NPs with SA‐TR. The red curve is fitted by a Hill function.

### 2.7 Single‐Particle Fluorescence Microscopy

Finally, given the exceptional absorbance and therefore brightness of the NPs‐Cy5, we explored the possibility of imaging these nanoparticles at the single‐particle level. Figure 7a presents a representative image of NPs‐Cy5 immobilized on the surface of a glass coverslip, revealing nanoscale emitters with size below the diffraction limit (∼325 nm). To further assess the detection of single particles, we analyzed 197 individual emitting nano‐objects diffusing in an homogenous 1:1 water‐glycerol solution (Movie S1, accessible on Zenodo). After detection, we performed superlocalization of the emitters and reconstructed their trajectories [61]. This allowed calculating the evolution of the mean square displacement (MSD, Equation S14) with lag time (Figure 7b). As expected for Brownian motion, a linear increase in MSD was observed. Using the known viscosity of 50% glycerol (9.00 ± 0.09 mPa·s) [62], we determined the diffusion constant of the particles to be 0.18 ± 0.01 µm^2^.s^−1^. Applying the Stokes‐Einstein equation (Equation S15),  this value corresponds to a hydrodynamic radius of 124 ± 10 nm, consistent with the detection of single NPs‐Cy5. Finally, from the trajectories, we extracted the intensity distribution of 2669 diffusing NPs‐Cy5, yielding a nearly unimodal distribution (median intensity of 752 detected photons), as anticipated for single‐nanoparticle detection (Figure 7c).

**FIGURE 7:**
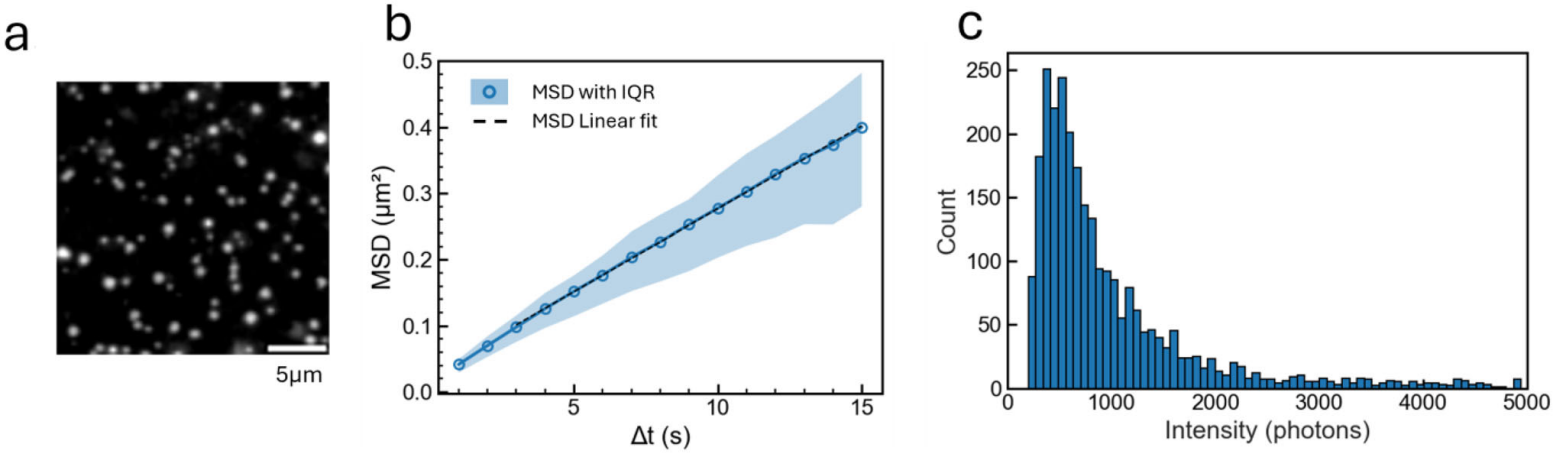
Single particle imaging and tracking: (a) Representative image of single NPs‐Cy5 in water dispersed on a coverslip. (b) The average MSD (with corresponding interquartile range, IQR) of 197 individual particles diffusing in a 1:1 water‐glycerol solution as a function of the lag time (Δ*t*), reveals Brownian motion with a diffusion coefficient of *D* = 0.18 µm^2^.s^−1^. (c) The intensity distribution of 2669 single particles is close to unimodal with a median intensity of 752 detected photons.

## 3 Conclusion

We report a one‐pot, fully aqueous strategy combining ROPISA of PLG‐NCA with CuAAC chemistry, enabling the direct formation of a unique class of anisotropic fluorescent polymer nanoparticles in a single step. This process operates at high solid contents (up to ∼7 wt%), far exceeding those typically accessible by nanoprecipitation (< 0.02 wt %) [17, 18], highlighting both its efficiency and scalability. This versatile approach enables the controlled incorporation of fluorophores during nanoparticle formation, yielding blue (Cou), green (NBD), and red (Cy5) emitting nanoparticles and allowing a detailed investigation of their spectroscopic properties. The polypeptide nature of these nanoparticles makes them potentially degradable, in contrast to conventional fluorescent nanomaterials based on inorganic or organic semiconductors, or vinyl polymers [11, 14, 17]. In addition, the covalent incorporation of dyes within the polymer chains enables tracking of the polymer architecture throughout the nanoparticle, providing enhanced robustness and stability in biological environments.

Combining NBD‐ and Cy5‐labeled nanoparticles reveals efficient FRET interactions driven by polymer chain exchange. This dynamic behavior provides a platform to probe energy transfer between anisotropic nano‐objects [12] and to investigate temporal and structural aspects of interparticle interactions. Their versatility is further illustrated by straightforward functionalization (e.g., biotin), enabling responsive FRET sensors based on the biotin–streptavidin interaction with ∼20% efficiency, comparable to established systems such as protein‐coated quantum dots or polymer dots.

Cy5‐labeled nanoparticles also exhibit exceptional brightness per unit volume, enabling detection as individual emitters. Combined with their red emission, this makes them well suited for biological applications such as deep‐tissue imaging [63] and single‐particle tracking [64, 65]. More broadly, their combination of high brightness, aqueous single‐step synthesis, high solid content, and soft anisotropic structure—typically achieved only in organic solvents [1, 2, 58, 66]—opens new opportunities in super‐resolution imaging [67, 68], biological soft matter studies [69], and spectroscopy. Their flexibility and anisotropic shape also make them attractive for investigating transport in crowded and heterogeneous media [70].

Most notably, their high brightness and deformability make these nanoparticles particularly promising for extending single‐particle tracking and diffusion studies to deep‐tissue environments [69, 71, 72], where they could enable access to regions that remain inaccessible to conventional probes.

## Author Contributions

**Amin El Jarroudi Hammou**: investigation, writing – original draft, validation, visualization, data curation. **Juliette Lavainne**: visualization, data curation, investigation. **Rohit Mangalwedhekar**: writing – original draft, visualization, investigation. **Jyothis Devasia**: investigation. **Laurent Cognet**: conceptualization, writing – review and editing, funding acquisition, supervision. **Sébastien Lecommandoux**: conceptualization, funding acquisition, writing – review and editing, supervision. **Colin Bonduelle**: conceptualization, funding acquisition, writing – review and editing, writing − original draft, supervision. **Chloé Grazon**: conceptualization, writing – review and editing, writing – original draft, supervision, project administration, validation, funding acquisition.

## Conflicts of Interest

The authors declare no conflicts of interest.

## Supporting information




**Supporting File 1**: Materials and methods, details on NCA synthesis including NMR spectra, FITR analysis, synthetic protocol of ROPISA‐CuAAC polymerization, including additional data, SEC chromatograms, FITR analysis, TEM images, fluorescence spectra between NPs interactions, FRET deconvolution spectra and FRET efficiency data.

**Supporting File 2**: anie73661‐sup‐0002‐MovieS1.avi.

## Data Availability

The data that support the findings of this study are openly available in Zenodo at https://doi.org/10.5281/zenodo.20809276.

## References

[1] F. J. M. Hoeben , P. Jonkheijm , E. W. Meijer , and A. P. H. J. Schenning , “About Supramolecular Assemblies of π‐Conjugated Systems,” Chemical Reviews 105, no. 4 (2005): 1491–1546, 10.1021/cr030070z. 15826018

[2] L. Maggini and D. Bonifazi , “Hierarchised Luminescent Organic Architectures: Design, Synthesis, Self‐Assembly, Self‐Organisation and Functions,” Chemical Society Reviews 41, no. 1 (2012): 211–241, 10.1039/C1CS15031F. 21748186

[3] H. Cabral , K. Miyata , K. Osada , and K. Kataoka , “Block Copolymer Micelles in Nanomedicine Applications,” Chemical Reviews 118, no. 14 (2018): 6844–6892, 10.1021/acs.chemrev.8b00199. 29957926

[4] M. A. Beach , U. Nayanathara , and Y. Gao , “Polymeric Nanoparticles for Drug Delivery,” Chemical Reviews 124, no. 9 (2024): 5505–5616, 10.1021/acs.chemrev.3c00705. PMC11086401 38626459

[5] C. Cummins , R. Lundy , J. J. Walsh , V. Ponsinet , G. Fleury , and M. A. Morris , “Enabling Future Nanomanufacturing Through Block Copolymer Self‐Assembly: A Review,” Nano Today 35 (2020): 100936, 10.1016/j.nantod.2020.100936.

[6] J. B. Grimm and L. D. Lavis , “Caveat Fluorophore: An Insiders' Guide to Small‐Molecule Fluorescent Labels,” Nature Methods 19, no. 2 (2022): 149–158, 10.1038/s41592-021-01338-6. 34949811

[7] A. H. Ashoka , I. O. Aparin , A. Reisch , and A. S. Klymchenko , “Brightness of Fluorescent Organic Nanomaterials,” Chemical Society Reviews 52, no. 14 (2023): 4525–4548, 10.1039/D2CS00464J. PMC10351213 37338018

[8] C. Grazon , M. Chern , and P. Lally , “The Quantum Dot vs. Organic Dye Conundrum for Ratiometric FRET‐Based Biosensors: Which One Would You Chose?,” Chemical Science 13, no. 22 (2022): 6715–6731, 10.1039/D1SC06921G. PMC9172442 35756504

[9] S. G. Stenspil and B. W. Laursen , “Photophysics of Fluorescent Nanoparticles Based on Organic Dyes—Challenges and Design Principles,” Chemical Science 15, no. 23 (2024): 8625–8638, 10.1039/D4SC01352B. PMC11168078 38873083

[10] W. R. Algar , M. Massey , and K. Rees , “Photoluminescent Nanoparticles for Chemical and Biological Analysis and Imaging,” Chemical Reviews 121, no. 15 (2021): 9243–9358, 10.1021/acs.chemrev.0c01176. 34282906

[11] I. V. Martynenko , A. P. Litvin , F. Purcell‐Milton , A. V. Baranov , A. V. Fedorov , and Y. K. Gun'ko , “Application of Semiconductor Quantum Dots in Bioimaging and Biosensing,” Journal of Materials Chemistry B 5, no. 33 (2017): 6701–6727, 10.1039/C7TB01425B. 32264322

[12] E. Madirov , C. Catros , N. Hildebrandt , and C. Grazon , “Inter‐Nanoparticle FRET for Biosensing: Photophysics Versus Size,” Angewandte Chemie International Edition 64, no. 39 (2025): e202510801, 10.1002/anie.202510801. PMC12455444 40810657

[13] A. Reisch and A. S. Klymchenko , “Fluorescent Polymer Nanoparticles Based on Dyes: Seeking Brighter Tools for Bioimaging,” Small 12, no. 15 (2016): 1968–1992, 10.1002/smll.201503396. PMC5405874 26901678

[14] C. Grazon , J. Rieger , R. Méallet‐Renault , B. Charleux , and G. Clavier , “Ultrabright Fluorescent Polymeric Nanoparticles Made From a New Family of BODIPY Monomers,” Macromolecules 46, no. 13 (2013): 5167–5176, 10.1021/ma400590q.

[15] A. Reisch , P. Didier , and L. Richert , “Collective Fluorescence Switching of Counterion‐Assembled Dyes in Polymer Nanoparticles,” Nature Communications 5, no. 1 (2014): 4089, 10.1038/ncomms5089. 24909912

[16] R. Méallet‐Renault , P. Denjean , and R. B. Pansu , “Polymer Beads as Nano‐Sensors,” Sensors and Actuators B: Chemical 59, no. 2 (1999): 108–112, 10.1016/S0925-4005(99)00205-1.

[17] K. Lix , M. V. Tran , and M. Massey , “Dextran Functionalization of Semiconducting Polymer Dots and Conjugation With Tetrameric Antibody Complexes for Bioanalysis and Imaging,” ACS Applied Bio Materials 3, no. 1 (2020): 432–440, 10.1021/acsabm.9b00899. 35019459

[18] B. Andreiuk , A. Reisch , M. Lindecker , et al., “Fluorescent Polymer Nanoparticles for Cell Barcoding In Vitro and In Vivo,” Small 13, no. 38 (2017): 1701582, 10.1002/smll.201701582. 28791769

[19] K. Trofymchuk , J. Valanciunaite , B. Andreiuk , A. Reisch , M. Collot , and A. S. Klymchenko , “BODIPY‐Loaded Polymer Nanoparticles: Chemical Structure of Cargo Defines Leakage From Nanocarrier in Living Cells,” Journal of Materials Chemistry B 7, no. 34 (2019): 5199–5210, 10.1039/C8TB02781A. 31364614

[20] N. J. W. Penfold , J. Yeow , C. Boyer , and S. P. Armes , “Emerging Trends in Polymerization‐Induced Self‐Assembly,” ACS Macro Letters 8, no. 8 (2019): 1029–1054, 10.1021/acsmacrolett.9b00464. 35619484

[21] B. Charleux , G. Delaittre , J. Rieger , and F. D'Agosto , “Polymerization‐Induced Self‐Assembly: From Soluble Macromolecules to Block Copolymer Nano‐Objects in One Step,” Macromolecules 45, no. 17 (2012): 6753–6765, 10.1021/ma300713f.

[22] P. Galanopoulo , N. Gil , and D. Gigmes , “RAFT‐Mediated Emulsion Polymerization‐Induced Self‐Assembly for the Synthesis of Core‐Degradable Waterborne Particles,” Angewandte Chemie International Edition 62, no. 16 (2023): e202302093, 10.1002/anie.202302093. 36821431

[23] L. Lei , T. P. T. Nguyen , and Y. De Rancourt De Mimérand , “PEG‐Based Core‐Degradable Nanoparticles via RAFT‐Mediated Reverse‐Sequence PISA,” Polymer Chemistry 17, no. 8 (2026): 789–795, 10.1039/D5PY01119A.

[24] T. J. Neal , L. Dalmasso , F. Coumes , and J. Rieger , “UV‐Degradable Poly(Phenyl Vinyl Ketone) Particles Produced by Polymerisation‐Induced Self‐Assembly in Ethanol or Water,” Polymer Chemistry 17, no. 3 (2026): 332–340, 10.1039/D5PY01020A.

[25] S. Zhang , R. Li , and Z. An , “Degradable Block Copolymer Nanoparticles Synthesized by Polymerization‐Induced Self‐Assembly,” Angewandte Chemie International Edition 63, no. 12 (2024): e202315849, 10.1002/anie.202315849. 38155097

[26] Y. Zhou , Z. Wang , and Y. Wang , “Azoreductase‐Triggered Fluorescent Nanoprobe Synthesized by RAFT‐Mediated Polymerization‐Induced Self‐Assembly for Drug Release,” Polymer Chemistry 11, no. 35 (2020): 5619–5629, 10.1039/D0PY00826E.

[27] J. Wang , Y. Zhu , and S. Zhou , “Nano‐Fluorophores Prepared by Polymerization‐Induced Self‐Assembly and Its Application in Cell Imaging,” Dyes and Pigments 190 (2021): 109353, 10.1016/j.dyepig.2021.109353.

[28] V. Ladmiral , T. M. Legge , Y. Zhao , and S. Perrier , ““Click” Chemistry and Radical Polymerization: Potential Loss of Orthogonality,” Macromolecules 41, no. 18 (2008): 6728–6732, 10.1021/ma8010262.

[29] Z. Geng , J. J. Shin , Y. Xi , and C. J. Hawker , “Click Chemistry Strategies for the Accelerated Synthesis of Functional Macromolecules,” Journal of Polymer Science 59, no. 11 (2021): 963–1042, 10.1002/pol.20210126.

[30] C. Grazon , P. Salas‐Ambrosio , and E. Ibarboure , “Aqueous Ring‐Opening Polymerization‐Induced Self‐Assembly (ROPISA) of N‐Carboxyanhydrides,” Angewandte Chemie International Edition 59, no. 2 (2020): 622–626, 10.1002/anie.201912028. 31650664

[31] R. Huisgen , Proceedings of the Chemical Society, October (1961): 357–396, 10.1039/PS9610000357.

[32] A. C. Engler , H. Lee , and P. T. Hammond , “Highly Efficient ‘Grafting Onto’ a Polypeptide Backbone Using Click Chemistry,” Angewandte Chemie International Edition 48, no. 49 (2009): 9334–9338, 10.1002/anie.200904070. PMC3870472 19902445

[33] S. W. Morton , X. Zhao , M. A. Quadir , and P. T. Hammond , “FRET‐Enabled Biological Characterization of Polymeric Micelles,” Biomaterials 35, no. 11 (2014): 3489–3496, 10.1016/j.biomaterials.2014.01.027. PMC4040354 24477190

[34] M. Nguyen , J.‐L. Stigliani , C. Bijani , P. Verhaeghe , G. Pratviel , and C. Bonduelle , “Ionic Polypeptide Polymers With Unusual β‐Sheet Stability,” Biomacromolecules 19, no. 10 (2018): 4068–4074, 10.1021/acs.biomac.8b01084. 30204420

[35] R. Su , L. Francés‐Soriano , and P. I. Diriwari , “FRET Materials for Biosensing and Bioimaging,” Chemical Reviews 125, no. 19 (2025): 9429–9551, 10.1021/acs.chemrev.5c00386. 40875965

[36] H. Beauseroy , F. Salimi , J. Aujard‐Catot , L. Alembik , S. Lecommandoux , and C. Bonduelle , “Aqueous Ring‐Opening Polymerization‐Induced Self‐Assembly (ROPISA): Tailoring Anisotropic Nanoparticles Through Amino Acid N‐Carboxyanhydride (NCA) Monomer Selection,” Macromolecules 58, no. 13 (2025): 6466–6479, 10.1021/acs.macromol.5c01089.

[37] T. P. Nguyen , A. D. Easley , and N. Kang , “Polypeptide Organic Radical Batteries,” Nature 593, no. 7857 (2021): 61–66, 10.1038/s41586-021-03399-1. 33953410

[38] H. Leuchs , “Ueber die Glycin‐Carbonsäure,” Berichte der Deutschen Chemischen Gesellschaft 39, no. 1 (1906): 857–861, 10.1002/cber.190603901133.

[39] M. Spyridakou , I. Tzourtzouklis , and R. Graf , “Multiple Levels of Organization in Amphiphilic Diblock Copolymers Based on Poly(γ‐Benzyl‐l‐Glutamate) Produced by Aqueous ROPISA,” Biomacromolecules 26, no. 3 (2025): 1892–1903, 10.1021/acs.biomac.4c01657. PMC11898064 39919302

[40] C. Grazon , P. Salas‐Ambrosio , and S. Antoine , “Aqueous ROPISA of α‐Amino Acid *N*‐Carboxyanhydrides: Polypeptide Block Secondary Structure Controls Nanoparticle Shape Anisotropy,” Polymer Chemistry 12, no. 43 (2021): 6242–6251, 10.1039/D1PY00995H.

[41] Y. Hu , Z.‐Y. Tian , and W. Xiong , “Water‐Assisted and Protein‐Initiated Fast and Controlled Ring‐Opening Polymerization of Proline *N*‐Carboxyanhydride,” National Science Review 9, no. 8 (2022): nwac033, 10.1093/nsr/nwac033. PMC9438472 36072505

[42] B. Liu , R. Fang , and W. Li , “Fast Catalyst‐Free Synthesis of Stereoselective Polypeptides via Hierarchical Chiral Assembly,” Journal of the American Chemical Society 146, no. 24 (2024): 16558–16566, 10.1021/jacs.4c03281. 38858162

[43] P. Ashokkumar , N. Adarsh , and A. S. Klymchenko , “Ratiometric Nanoparticle Probe Based on FRET‐Amplified Phosphorescence for Oxygen Sensing With Minimal Phototoxicity,” Small 16, no. 32 (2020): 2002494, 10.1002/smll.202002494. 32583632

[44] K. Sivakumar , F. Xie , B. M. Cash , S. Long , H. N. Barnhill , and Q. Wang , “A Fluorogenic 1,3‐Dipolar Cycloaddition Reaction of 3‐Azidocoumarins and Acetylenes,” Organic Letters 6 , no. 24 (2004): 4603–4606, 10.1021/ol047955x. 15548086

[45] T. Luu , K. Gristwood , J. C. Knight , and M. Jörg , “Click Chemistry: Reaction Rates and Their Suitability for Biomedical Applications,” Bioconjugate Chemistry 35, no. 6 (2024): 715–731, 10.1021/acs.bioconjchem.4c00084. PMC11191409 38775705

[46] K. Nian , Y. Liu , and Y. Qiu , “In Cu‐Click: DNA‐Enhanced Ligand Enables Live‐Cell, Intracellular Click Chemistry Reaction With Copper Catalyst,” Nature Communications 16, no. 1 (2025): 4788, 10.1038/s41467-025-60143-3. PMC12102219 40410175

[47] Z. Zhou and C. J. Fahrni , “A Fluorogenic Probe for the Copper(I)‐Catalyzed Azide−Alkyne Ligation Reaction: Modulation of the Fluorescence Emission via ^3^(n,Π*)−^1^(π,Π*) Inversion,” Journal of the American Chemical Society 126, no. 29 (2004): 8862–8863, 10.1021/ja049684r. 15264794

[48] S. I. Presolski , V. Hong , S.‐H. Cho , and M. G. Finn , “Tailored Ligand Acceleration of the Cu‐Catalyzed Azide−Alkyne Cycloaddition Reaction: Practical and Mechanistic Implications,” Journal of the American Chemical Society 132, no. 41 (2010): 14570–14576, 10.1021/ja105743g. PMC2956586 20863116

[49] T. Y. Leung , L. Qi , K. Liu , D. Sen , and H.‐Z. Yu , “Short DNAzymes for Efficient Catalysis of ‘Click’ Reactions in Solution and on Surface,” Langmuir 40, no. 39 (2024): 20576–20583, 10.1021/acs.langmuir.4c02314. 39305250

[50] Z. Tian , A. D. Shaller , and A. D. Q. Li , “Twisted Perylene Dyes Enable Highly Fluorescent and Photostable Nanoparticles,” Chemical Communications 45, no. 2 (2009): 180–182, 10.1039/B815507K. 19099061

[51] S. Egloff , N. Melnychuk , E. Cruz Da Silva , A. Reisch , S. Martin , and A. S. Klymchenko , “Amplified Fluorescence In Situ Hybridization by Small and Bright Dye‐Loaded Polymeric Nanoparticles,” ACS Nano 16, no. 1 (2022): 1381–1394, 10.1021/acsnano.1c09409. 34928570

[52] A. Wagh , S. Y. Qian , and B. Law , “Development of Biocompatible Polymeric Nanoparticles for In Vivo NIR and FRET Imaging,” Bioconjugate Chemistry 23, no. 5 (2012): 981–992, 10.1021/bc200637h. 22482883

[53] C. Würth , M. Grabolle , J. Pauli , and M. Spieles , “Resch‐Genger, U. Relative and Absolute Determination of Fluorescence Quantum Yields of Transparent Samples,” Nature Protocols 8, no. 8 (2013): 1535–1550, 10.1038/nprot.2013.087.. 23868072

[54] J. M. Drake , M. L. Lesiecki , and D. M. Camaioni , “Photophysics and Cis‐Trans Isomerization of DCM,” Chemical Physics Letters 113, no. 6 (1985): 530–534, 10.1016/0009-2614(85)85026-0.

[55] R. Sens and K. H. Drexhage , “Fluorescence Quantum Yield of Oxazine and Carbazine Laser Dyes,” Journal of Luminescence 24–25 (1981): 709–712, 10.1016/0022-2313(81)90075-2.

[56] W. R. Algar , N. Hildebrandt , S. S. Vogel , and I. L. Medintz , “FRET as a Biomolecular Research Tool — Understanding Its Potential While Avoiding Pitfalls,” Nature Methods 16, no. 9 (2019): 815–829, 10.1038/s41592-019-0530-8. 31471616

[57] P. Rajdev and S. Ghosh , “Fluorescence Resonance Energy Transfer (FRET): A Powerful Tool for Probing Amphiphilic Polymer Aggregates and Supramolecular Polymers,” Journal of Physical Chemistry B 123, no. 2 (2019): 327–342, 10.1021/acs.jpcb.8b09441. 30407823

[58] Y.‐C. Lin and S.‐W. Kuo , “Hierarchical Self‐Assembly and Secondary Structures of Linear Polypeptides Graft Onto POSS in the Side Chain Through Click Chemistry,” Polymer Chemistry 3, no. 1 (2011): 162–171, 10.1039/C1PY00381J.

[59] T. Nicolai , O. Colombani , and C. Chassenieux , “Dynamic Polymeric Micelles Versus Frozen Nanoparticles Formed by Block Copolymers,” Soft Matter 6, no. 14 (2010): 3111, 10.1039/B925666K.

[60] K. Lix , K. D. Krause , H. Kim , and W. R. Algar , “Investigation of the Energy Transfer Mechanism Between Semiconducting Polymer Dots and Organic Dyes,” Journal of Physical Chemistry C 124, no. 31 (2020): 17387–17400, 10.1021/acs.jpcc.0c04983.

[61] L. Cognet , C. Leduc , and B. Lounis , “Advances in Live‐Cell Single‐Particle Tracking and Dynamic Super‐Resolution Imaging,” Current Opinion in Chemical Biology 20 (2014): 78–85, 10.1016/j.cbpa.2014.04.015. 24875636

[62] A. Volk and C. J. Kähler , “Density Model for Aqueous Glycerol Solutions,” Experiments in Fluids 59, no. 5 (2018): 75, 10.1007/s00348-018-2527-y.

[63] J. V. Frangioni , “In Vivo Near‐Infrared Fluorescence Imaging,” Current Opinion in Chemical Biology 7, no. 5 (2003): 626–634, 10.1016/j.cbpa.2003.08.007. 14580568

[64] N. Ruthardt , D. C. Lamb , and C. Bräuchle , “Single‐Particle Tracking as a Quantitative Microscopy‐Based Approach to Unravel Cell Entry Mechanisms of Viruses and Pharmaceutical Nanoparticles,” Molecular Therapy 19, no. 7 (2011): 1199–1211, 10.1038/mt.2011.102. PMC3129551 21654634

[65] D. Jin , P. Xi , B. Wang , L. Zhang , J. Enderlein , and A. M. van Oijen , “Nanoparticles for Super‐Resolution Microscopy and Single‐Molecule Tracking,” Nature Methods 15, no. 6 (2018): 415–423, 10.1038/s41592-018-0012-4. 29808018

[66] Z. M. Hudson , D. J. Lunn , M. A. Winnik , and I. Manners , “Colour‐Tunable Fluorescent Multiblock Micelles,” Nature Communications 5, no. 1 (2014): 3372, 10.1038/ncomms4372. 24594554

[67] A. M. Szalai , B. Siarry , and J. Lukin , “Super‐Resolution Imaging of Energy Transfer by Intensity‐Based STED‐FRET,” Nano Letters 21, no. 5 (2021): 2296–2303, 10.1021/acs.nanolett.1c00158. 33621102

[68] W. Li , G. S. Kaminski Schierle , B. Lei , Y. Liu , and C. F. Kaminski , “Fluorescent Nanoparticles for Super‐Resolution Imaging,” Chemical Reviews 122, no. 15 (2022): 12495–12543, 10.1021/acs.chemrev.2c00050. PMC9373000 35759536

[69] J. Estaun‐Panzano , S. Nandi , and Q. Gresil , “Intracellular α‐Synuclein Assemblies Are Sufficient to Alter Nanoscale Diffusion in the Striatal Extracellular Space,” NPJ Parkinsons Disease 10, no. 1 (2024): 236, 10.1038/s41531-024-00850-8. PMC11686398 39738158

[70] N. Fakhri , F. C. MacKintosh , B. Lounis , L. Cognet , and M. Pasquali , “Brownian Motion of Stiff Filaments in a Crowded Environment,” Science 330, no. 6012 (2010): 1804–1807, 10.1126/science.1197321. 21205665

[71] A. Lee , A. A. Simon , and A. Boyreau , “Identification of Early Stage Liver Fibrosis by Modifications in the Interstitial Space Diffusive Microenvironment Using Fluorescent Single‐Walled Carbon Nanotubes,” Nano Letters 24, no. 18 (2024): 5603–5609, 10.1021/acs.nanolett.4c00955. 38669477

[72] A. G. Godin , J. A. Varela , and Z. Gao , “Single‐Nanotube Tracking Reveals the Nanoscale Organization of the Extracellular Space in the Live Brain,” Nature Nanotech 12, no. 3 (2017): 238–243, 10.1038/nnano.2016.248. 27870840

