# Supplementary material for: Click'n ROPISA: One‐Pot Synthesis of Ultrabright Fluorescent Nanoparticles Made of Polypeptides
Source: Angew Chem Int Ed Engl. 2026 Jul 15;65(41):e1373955. doi: 10.1002/anie.1373955 (PMC13638097; doi:10.1002/anie.1373955)
Supplement: Supplementary file 1 [file ANIE-65-e1373955-s001.pdf]

Electronic Supplementary Information

Click'n ROPISA: One-pot synthesis of Ultrabright Fluorescent nanoparticles made of polypeptides

Amin El Jarroudi Hammou,[1,2] Juliette Lavainne,[1] Rohit Mangalwedhekar,[3,4] Jyothis Devasia,[1] Laurent Cognet,[3,4] Sébastien Lécommandoux,[2,5] Colin Bonduelle,[2] Chloé Grazon[1, 2]

1. Univ. Bordeaux, CNRS, Bordeaux INP, ISM, UMR 5255, F-33400 Talence, France
2. Univ. Bordeaux, CNRS, Bordeaux INP, LCPO, UMR 5629, F-33600 Pessac, France
3. Laboratoire Photonique Numérique et Nanosciences, Univ. Bordeaux, 33400 Talence, France
4. LP2N, Institut d'Optique Graduate School, CNRS – UMR 5298, 33400 Talence, France
5. Institut Universitaire de France (IUF)

Corresponding Authors
Chloé Grazon
Email : chloe.grazon@u-bordeaux.fr
Colin Bonduelle
Email : colin.bonduelle@enscbp.fr

Table of contents

1. Chemicals ... 2
2. Methods ... 2
3. Calculations ... 4
4. Synthesis procedures ... 7
4.1. Synthesis of ɣ-Propargyl-L-glutamate *N*-Carboxyanhydride (PLG-NCA) ... 7
4.2. Synthesis of N-(3-Azidopropyl)-7-nitro-2,1,3-benzoxadiazol-4-amine (NBD-N_3) ... 10
4.3. Ring Opening Polymerization of PLG-NCA ... 12
4.4. Synthesis of nanoparticles (NPs) by ROPISA ... 13
5. Spectra analysis ... 24
5.1. Fluorescence spectra of NBD-NPs with Cy5-NPs ... 24
5.2. Fluorescence spectra of NBD-NPs with Cy5-NPs after ultrasounds ... 24
5.3. Fluorescence spectra of BtNBD-NPs with SA-TR ... 26
References ... 27

1. Chemicals

All commercially-available chemicals were used as received. L-glutamic acid was purchased from thermo scientific. Propargyl alcohol, di-tert-butyl dicarbonate (Boc_2O), sulfuric acid (H_2SO_4), tetrahydrofuran (THF), thionyl chloride ($SOCl_2$), copper sulphate pentahydrate ($CuSO_4.5H_2O$), L-ascorbic acid and sodium bicarbonate were supplied from Sigma Aldrich. Amino-poly(ethylene glycol) (PEG_{113}-NH_2, M_n=5000 g.mol^{-1}, $Đ$=1.05) was purchased from RAPP Polymer Gmbh (Tübingen, Germany). Biotin-poly(ethylene glycol) (Bt-PEG_{71}-NH_2, M_n=3400g.mol^{-1}, $Đ$=1.06) was supplied by Laysan Bio. γ-Benzyl-L-glutamate *N*-carboxyanhydride (BLG-NCA) was supplied from PMC Isochem. 3-azido-7-hydroxycoumarin (Cou-N_3) was purchased from Tokyo Chemical Industry. Cyanine 5 azide (Cy5-N_3) was purchased from Lumiprobe GmbH. Streptavidin Texas-red was purchased from Vector laboratories. Ultrapure water was obtained from a Millipore's Simplicity ® water purification system with a resistivity of 18.2 MΩ.cm^{-1}.

2. Methods

^{1}H and ^{13}C NMR spectra were recorded at the CESAMO platform (ISM, univ. Bordeaux), either on a Bruker Avance I 300 spectrometers operating at 300 MHz or 75 MHz respectively, or on a Bruker Avance III spectrometer equipped with a 5 mm BBFO (broad band fluorine observation) liquid probe, at 298 K operating at 400 MHz or 100 MHz respectively. NMR spectra were also recorded in LCPO using a Bruker Avance 400 at 400 MHz or 75MHz. Chemical shifts (δ) are reported in ppm and residual nondeuterated solvent were used as an internal reference. Coupling constants (J) are given in Hertz. The deuterated solvents are supplied by Eurisotop (DMSO) or Sigma-Aldrich (D_2O and $CDCl_3$).

Size Exclusion Chromatography (SEC). Polymer molar masses were determined by SEC using dimethylformamide (DMF + LiBr 1 g.L^{-1} or 8.7 g.L^{-1}) as eluent. Measurements were performed on an Ultimate 3000 system from ThermoFischer Scientific (Ilkirch, France) equipped with a diode array detector (DAD). The system also includes a differential refractive index detector dRI from Wyatt technology (Santa Barbara CA, USA). Polymers were separated on three Shodex Asahipack gel columns [GF 310 (7.5 × 300 mm^2), GF510 (7.5 × 300 mm^2), exclusion limits from 500–300 000 Da] at a flowrate of 0.5 mL.min^{-1}. Columns temperature was held at 50 °C. Easivial™ kit of Polystyrene from Agilent (Santa Clara CA, USA) was used as calibration standard (M_n from 162 to 364 000 Da).

Fourier transform infrared (FTIR). FTIR spectra were collected on a Bruker Vertex 70 or on a JASCO FT/IR-4600 spectrometers equipped with a diamond ATR tool. The powders are deposited directly on the crystal and the spectra were recorded in the spectral region from 1000 to 2000 cm^{-1} using 32 or 64 scans with a resolution of 4 cm^{-1}. A background was recorded before loading the samples onto the ATR crystal for measurements.

Transmission Electron Microscopy (TEM). The images were recorded on a Hitachi H7650 microscope working at 80 kV. Samples were prepared by deposing a drop of 0.1 mg. mL^{-1} NP dispersion onto a copper grid and removing excess after 2 min. Grids were negatively stained with 1% uranyl acetate to contrast the objects.

Cryo-TEM. For cryo-TEM observation, grids were mounted onto a Gatan 626 cryoholder and transferred to a Tecnai F20 microscope (Thermo Fisher Scientific) operated at 200 kV. Images were recorded with an Eagle 2k CCD camera (FEI, USA).

Differential scanning calorimetry (DSC). Differential scanning calorimetry (DSC) measurements were performed using a TA Instruments DSC Q200 LN2 apparatus with a heating and cooling ramp of 10°C min^{-1}. Melting temperatures and glass transition temperatures were determined using the TA Universal Analysis software.

Thermogravimetric analysis (TGA). Thermo-gravimetric measurements (TGA) of polymers samples (~10 mg) were performed on a TA Instruments Q500 from room temperature to 200°C with a heating rate of 10°C.min^{-1}. The analyses were investigated under nitrogen atmosphere with platinum pans.

Inductively coupled plasma optical emission spectroscopy. ICP-OES analysis were carried out at ICMCB-Bordeaux, France to evaluate the quantity of residual copper after dialysis purification. The samples were mineralized by treatment with hydrochloric acid at 60 °C, followed by dilution prior to analysis. The solutions were measured five times using an Agilent 5800 ICP-OES, after calibration of copper using a standard curve prepared from a 1 g. L^{-1} mono-elemental solution diluted in 25 mL volumetric flasks.

Photophysical characterizations. The absorption and fluorescence emission spectra were respectively recorded on a Jasco V670 and an Edinburgh instruments FLS 1000 spectrometers. Samples were analyzed in spectroscopic grade solvents or in distilled water. The solutions were placed in quartz cuvettes with an optical path length of 1 cm, except for the thermodynamically study (Arrhenius plot), where samples were analyzed in PMMA microcuvette of 500 µL (path length = 10 mm). All fluorescence emission spectra were corrected for a device function and recorded at 90° on samples with an absorbance of less than 0.1 at the excitation wavelength. For each sample, the fluorescence quantum yield (ϕ_F) was measured three times and calculated relative to a reference[1] (quinine bisulfate ϕ_F = 0.546 in 0.5 M H_2SO_4 for Cou-N_3,[2] 4-Dicyanomethane (DCM) ϕ_F=0.437 in EtOH for NBD-N_3,[3] Nile Blue (NB) ϕ_F =0.27 in EtOH for Cy5-N_3 [4]) using the equation:

$$\phi_F = \phi_{F,ref} \times \frac{Grad}{Grad_{ref}} \times \frac{n^2}{n_{ref}^2} \quad (1)$$

Where $\phi_{F,ref}$ is the fluorescence quantum yield of the reference, *Grad* and $Grad_{ref}$ are respectively the slopes of the integrated fluorescence intensity *vs* absorbance at the excitation wavelength for the sample and reference, and n^2 and n^2_{ref} are respectively the refractive indices of the sample and reference solutions.

Fluorescence lifetime measurements were performed on an Edinburgh Instruments FLS1000 using a Horiba NanoLed with Controler NL-C2 for excitations at λ^{exc}=370nm for Cou-NPs, at λ^{exc}=455nm for NBD-NPs and at λ^{exc}=670nm for Cy5-NPs. Aqueous dispersion of Ludox (30% in water, Sigma–Aldrich) was used to record the instrument response (IRF). Fluorescence decays (I(t)) were adjusted using the reconvolution method of the Fluoracle software. The NPs fluorescence decays in water were fitted with a sum of two exponentials:

$$I(t) = \sum_i a_i \, e^{-\frac{t}{\tau_i}} \quad (2)$$

Where I is the normalized fluorescence intensity, t is the delay time and a_i are the normalized pre-exponential coefficients and τ_i the individual fluorescence lifetimes. The amplitude average fluorescence lifetime (< τ >) is calculated using:

$$<\tau \geq \sum_i a_i \tau_i \quad (3)$$

Single-particle tracking measurements. First, to image immobilized single particles, the freeze-dried NPs-Cy5 were dispersed in ultrapure water at a concentration of 1 mg.mL^{-1}. This stock solution was then diluted 50 times in ultrapure water, and spincoated onto a clean glass coverslip to obtain a homogenous distribution of immobilized nanoparticles on the glass surface. Next, to image diffusing particles, the stock was diluted 50 times in a 1:1 ultrapure water-glycerol (Sigma Aldrich) solution. A slide was prepared by carefully sandwiching 1 µL of the prepared sample between a glass slide and a coverslip using a 50 µm imaging spacer in-between. The sample prepared was imaged on a Nikon Eclipse Ti inverted microscope equipped with a 60× water-immersion objective (NA 1.27). Movies of diffusing particles were recorded. Single particles were localized using the ThunderSTORM[5] plugin on Fiji ImageJ, followed by trajectory reconstruction using custom python codes built on the Trackpy[6,7] library.

3. Calculations

Theorical Molar Mass

The theoretical molar mass is the molar mass calculated from the known molecular structure and reaction parameters, assuming a full conversion and that all dyes are clicked:

$$M_n{}^{th} = M_I + \frac{1}{[I]} \times (M_{dye} \times [dye] + (M_{R-NCA)} - M_{co_2}) \times [R - NCA]) \quad (4)$$

Where $M_n{}^{th}$ is defined as the theorical molar mass (g.mol^{-1}), M_I is the molar mass of the initiator, [I] is the concentration of the initiator, M_{dye} is the molar mass of the dye, [dye] is the concentration of the dye, $M_{R\text{-}NCA}$ is the molar mass of the NCA, M_{CO2} is the molar mass of CO_2 that triggers the reaction and [R-NCA] is the concentration of the NCA.

Determination of dye %

The molar percentage of dye per polymer after the ROPISA-CuAAC polymerization and purification by dialysis is estimated by measuring the absorbance of a given mass of freeze-dried NPs recovered in a known volume of water, using the appropriated molar absorption coefficient of the dye ($\varepsilon_{\text{Cou}}{}^{\text{max}}$=10900 M^{-1}cm^{-1},[8] $\varepsilon_{\text{NBD}}{}^{\text{max}}$=20000 M^{-1}cm^{-1} and $\varepsilon_{\text{Cy5}}{}^{\text{max}}$ = 250000 M^{-1}cm^{-1} [9]) and using the Beer-Lambert equation:

$$A = \varepsilon \times \text{l} \times \text{c} \quad (5)$$

Brightness per volume the brightness per volume (B_V) of the doped nanoparticles is calculated assuming that the fluorescent core is only constituted of the dye and the PPLG, using the following equation,

$$\text{B}_\text{V} = \frac{\rho \times \text{Na} \times \varepsilon \times \phi_F \times w_{dye}}{M_{dye}} \quad (6)$$

Where Na is the Avogadro's number (6.022×10^{23} mol^{-1}), the ε is the molar extinction coefficient of the dye (in $M^{-1}.cm^{-1}$), ϕ_F is the quantum yield of the NP, ρ is the density of the NP hydrophobic core, assuming $\rho=1.35 g.cm^{-3}$ for polypeptides[10], w_{dye} is the mass fraction of the dye in the hydrophobic core and M_{dye} is the molar mass of the dye.

Spectra deconvolution. Spectral unmixing was performed to quantify the relative contributions of donor and acceptor fluorescence to each recorded FRET spectrum using non-negative least squares (NNLS) regression. The method assumes that each measured spectrum can be described as a linear combination of donor and acceptor reference spectra. Specifically, the measured spectrum (S(λ)) was modeled as: $S(\lambda) = c_D \cdot D(\lambda) + c_A \cdot A(\lambda) + \varepsilon(\lambda)$
where D(λ) and A(λ) represent the averaged donor-only and acceptor-only reference spectra, respectively, c_D and c_A are non-negative scalar coefficients (≥0) corresponding to their relative contributions, and ε(λ) denotes the residual error.
Reference spectra were generated by averaging all donor-only and acceptor-only measurements after subtraction of the mean background signal and interpolation onto a common wavelength axis. For each FRET sample, background fluorescence was removed using a corresponding background spectrum acquired under matching conditions.
The donor (F_D or F_{DA}) and acceptor (F_A) fluorescence contributions were obtained from the fitted components ($c_D \cdot D(\lambda)$) and ($c_A \cdot A(\lambda)$), respectively. All data processing steps, including reference spectrum generation, interpolation, background subtraction, NNLS fitting, residual analysis, and generation of publication-quality figures and CSV output tables, were implemented in a single automated Python script using NumPy, SciPy, Pandas, and Matplotlib.

FRET efficiency The FRET efficiency is defined as:

$$E = 1 - \frac{F_{DA}}{F_D} \quad (7)$$

Where F_{DA} is the donor fluorescence intensity in the presence of the acceptor and F_D is the donor fluorescence intensity in the absence of the acceptor.

The Förster radius is calculated using:

$$R_0 = 0.021 \times (\kappa^2\, n^{-4}\, \phi_D\, J)^{\frac{1}{6}} \quad (8)$$

Where κ^2 is the orientation factor between the donor and acceptor transition dipoles (≈2/3 for random orientation), n is the refractive index of the medium, for the intra FRET assays n=1.59 (organic core) and for the core-shell FRET assays n=1.33 (water), ϕ_D is the quantum yield of the donor and J is the spectral overlap integral between the donor emission and the acceptor absorption, calculated as:

$$J = \int f_D(\lambda)\ \varepsilon_A(\lambda)\ \lambda^4\ d\lambda \quad (9)$$

The Stern-Volmer equation for fluorescence quenching is defined as:

$$\frac{F_0}{F} = 1 + K_{SV}[Q] \quad (10)$$

Where, F_0 is the fluorescence intensity of the donor in the absence of quencher, F is the fluorescence intensity in the presence of the quencher. F_0 and F are determined by the spectra deconvolution method. K_{SV} is the Stern-Volmer quenching constant and $[Q]$ is the quencher

concentration. For purely dynamic (collisional) quenching, a linear Stern–Volmer plot is expected.

Energy of activation of inter-chains exchanges between nanoparticles is calculated using the Arrhenius equation:

$$k = Ae^{\frac{-E_a}{RT}} \tag{11}$$

Where k is the rate constant, T is the absolute temperature, A is the pre-exponential factor or Arrhenius factor, E_a is the molar activation energy for the reaction and R is the universal gas constant. To determine the energy of activation, the formula may be expressed as:

$$\ln k = \ln A - \frac{E_a}{R}\frac{1}{T} \tag{12}$$

The mathematic function used to extract the rate constant k for the Arrhenius plot is:

$$y = y_0 + Ae^{(-kt)} \tag{13}$$

Single particle tracking and diffusion analysis

The Mean Squared Displacement (MSD) was calculated and fitted by:

$$MSD(\Delta t) = \epsilon - \frac{4}{3} \times \ Dt_E + 4Dt \tag{14}$$

Here, Δt represents the lag time, t_E represents the exposure time of the camera, ϵ relates to the localization uncertainty (σ) for a static particle by $\epsilon = \sigma^2$, and D is the diffusion coefficient. Using the fitted diffusion coefficient, we determine the static localization uncertainty to be σ=57 nm. Finally, the hydrodynamic radius (r) of the particle was approximated using the Stokes-Einstein relation given by:

$$D = \frac{k_B T}{6\pi\eta r} \tag{15}$$

Where k_B is the Boltzmann coefficient, T is the absolute temperature and η is the viscosity of the solution in Pa.s. The distribution of particle intensities was obtained by extracting the intensity of the first localization of 2669 trajectories.

4. Synthesis procedures

4.1. Synthesis of ɣ-Propargyl-L-glutamate *N*-Carboxyanhydride (PLG-NCA)

For the synthesis of PLG-NCA, the protocol was adapted from [11–14].

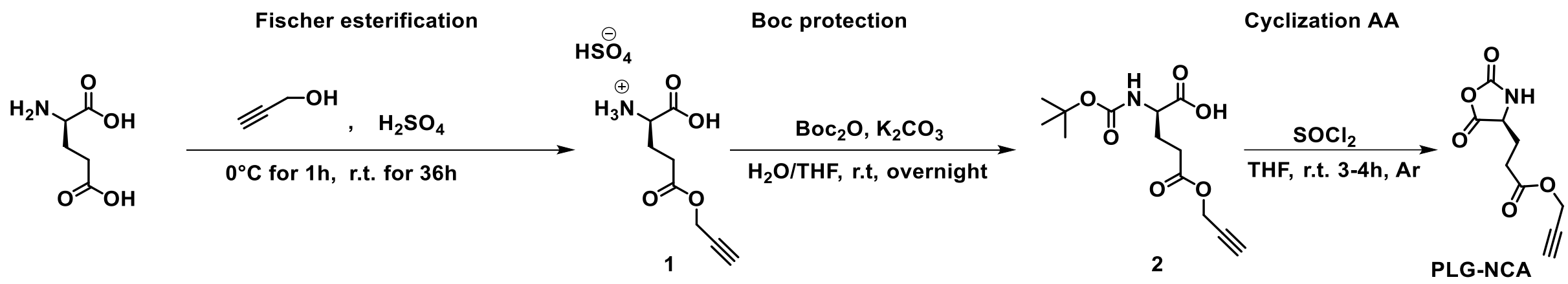

Scheme SI 1: Propargyl-L-glutamate N-carboxyanhydride synthesis (PLG-NCA) in 3 steps: esterification, Boc protection and NCA formation through the Leuch's method.

4.1.1. Synthesis of ɣ-Propargyl-L-glutamate (1)

L-glutamic acid (1.00 g, 6.8 mmol) was suspended in propargyl alcohol (20 mL, 0.34 mol, 50 equiv.) in a three-neck round-bottom flask under an argon atmosphere. Concentrated sulfuric acid (0.55 mL, 10.2 mmol, 1.5 equiv.) was added dropwise at 0 °C. The reaction mixture was stirred at 0 °C for 1 hour, then allowed to warm to room temperature. It was stirred for an additional 36 hours, until the amino acid had fully dissolved. The reaction mixture was then filtered, and the product was precipitated by addition into cold diethyl ether. A white-to-brown solid precipitated and was collected by centrifugation. The solid was washed and dried under vacuum to yield the product as a white/brown powder (m=1.3g, molar yield= 87%).

^{1}H NMR (400MHz, D_2O, δ, ppm): 4.75 (d, *J* = 2.5 Hz, 2H, CH_2O), 4.12 (t, *J* = 6.7 Hz, 1H, CH), 2.93 (t, *J* = 2.5 Hz, 1H, CCH), 2.69 (td, *J* = 7.5, 2.4 Hz,2H, CO-CH_2-CH_2-CH), 2.29 (m, 2H, CO-CH_2-CH_2-CH).

4.1.2. Synthesis of Boc-γ-propargyl-L-glutamate N-Carboxyanhydride (2)

Propargyl-L-glutamate (550 mg, 2.5 mmol) was dissolved in a 1:1 mixture of water and tetrahydrofuran (80 mL) in a round-bottom flask. Then, di-tert-butyl dicarbonate (Boc_2O, 1 g, 5.0 mmol) and potassium carbonate (K_2CO_3, 700 mg, 5.0 mmol) were added to the solution. The reaction mixture was stirred overnight at room temperature. After completion, the solvent was partially removed under reduced pressure, leaving a small amount of liquid. Then, water (25 mL) was added to the residue, and the mixture was extracted three times with diethyl ether using a separatory funnel. The combined organic phases were set aside. The remaining aqueous phase was acidified dropwise with 6 M HCl until a white suspension was formed. The acidified solution was then extracted four times with diethyl ether. The combined ether extracts were dried over anhydrous sodium sulfate, filtered, and concentrated under reduced pressure to yield a yellowish oil that was identified as Boc-γ-propargyl-L-glutamate. (m = 593 mg, molar yield= 84%).

^{1}H NMR (400MHz, $CDCl_3$, δ, ppm): 5.17-5.15 (d, *J* = 8.1 Hz, 1H, NH), 4.68-4.67 (d, *J* = 2.5 Hz 2H, CH_2O), 4.35-4.34 (d, *J* = 5.5 Hz 1H, CH), 2.51-2.49 (m, 2H, CH_2CH_2O), 2.47 (t, *J* = 2.5 Hz 1H, CCH), 2.22-2.00(m, 2H, CH_2CH_2O), 1.43(s, 9H, Boc).

4.1.3. Cyclization into the Propargyl-L-glutamate N-carboxyanhydride (PLG-NCA)

In a flame-dried Schlenk flask under an argon atmosphere, Boc-γ-propargyl-L-glutamate (430 mg, 1.5 mmol) was dissolved in 5 mL of dry tetrahydrofuran (THF). Then, thionyl chloride

($SOCl_2$, 0.2 mL, 2.2 mmol) was added dropwise to the reaction mixture, which was stirred at room temperature under argon for 4 hours. ^{1}H NMR spectroscopy was used to monitor the reaction progress and confirm the complete cyclization of the amino acid to the corresponding *N*-carboxyanhydride (NCA). Once complete, the solvent was removed under reduced pressure. The resulting yellow oil was redissolved in dry THF and filtered through a celite pad to purify it. The purified solution was transferred under argon with a cannula into a second Schlenk flask. The solvent was then removed under vacuum to yield a yellow/orange viscous oil. The crude product was further purified by washing with cold pentane to afford the final NCA product as an orange oil (m = 213mg, molar yield = 67%, purity >95%). The synthesized PLG-NCA was stored at -10°C under argon atmosphere and weighed in an argon-saturated atmosphere glove box.

^{1}H NMR (400MHz, $CDCl_3$, δ, ppm): 6.48 (d, 1H, NH); 4.72-4.71 (d, J = 2.5 Hz, 2H, CH_2O), 4.42 (t, J = 6.1 Hz, 1H, CHCO), 2.62 (t, J = 6.8 Hz, 2H, CH_2CH_2O), 2.52 (t, J = 2.5 Hz 1H, CCH), 2.30-2.16 (m, 2H, CH_2CH_2O).

^{13}C NMR (400MHz, $CDCl_3$, δ, ppm): 171.9 (CH_2COO), 169.3 (COO (ring)), 151.6 (OCNH), 77.2 (CCH), 75.7 (CCH), 57.0(CHNH), 52.8(CH_2CO), 29.9 (CH_2COO), 27.05 (CH_2).

FITR: Characteristic bands of NCA C=O elongations at **1787**cm^{-1} and **1854**cm^{-1}.

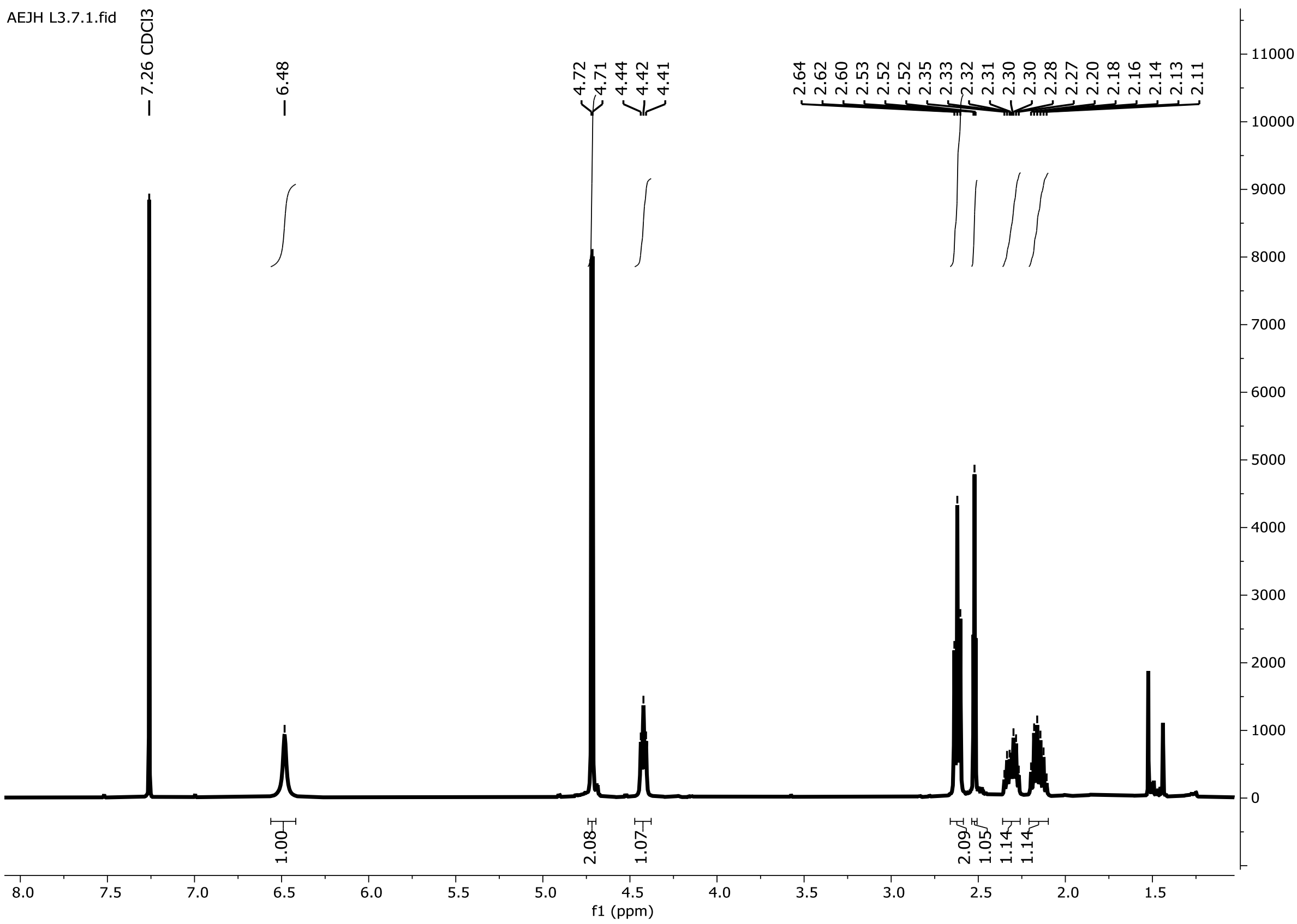

Figure SI 1: ^{1}H NMR of PLG-NCA in $CDCl_3$.

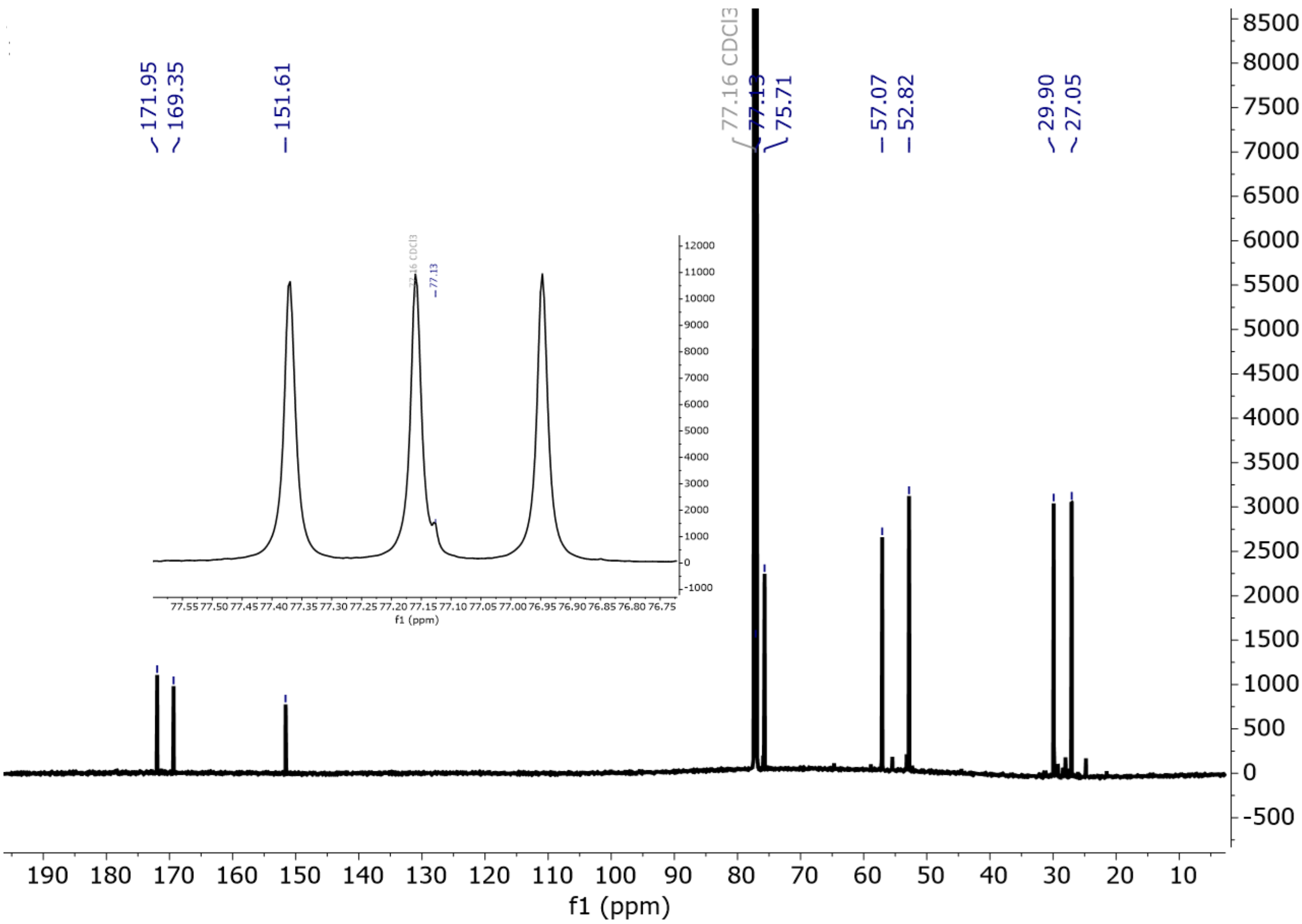

Figure SI 2: ^{13}C NMR of NCA-PLG in $CDCl_3$.

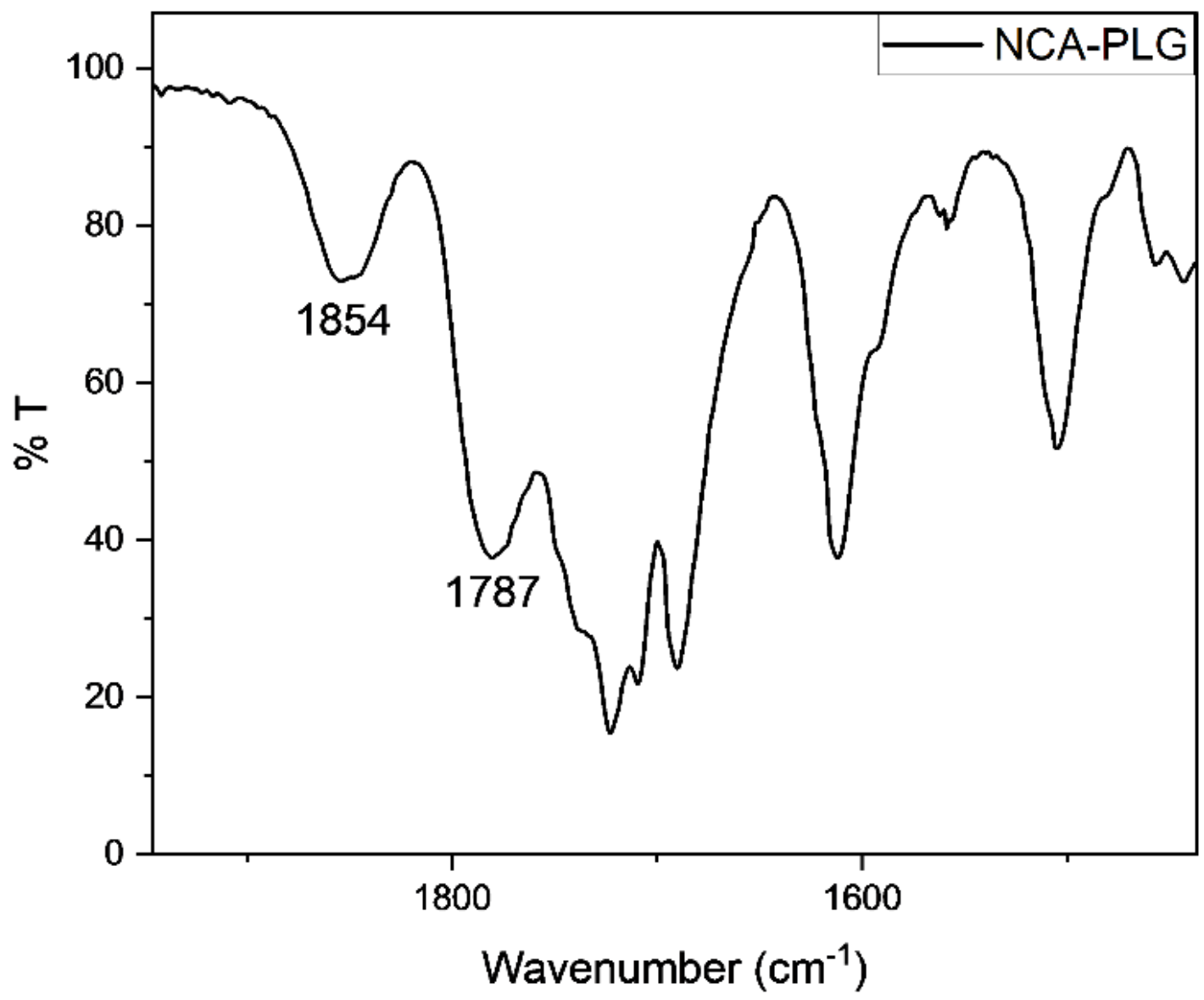

Figure SI 3: FTIR of PLG-NCA.

4.2. Synthesis of N-(3-Azidopropyl)-7-nitro-2,1,3-benzoxadiazol-4-amine ($NBD-N_3$)

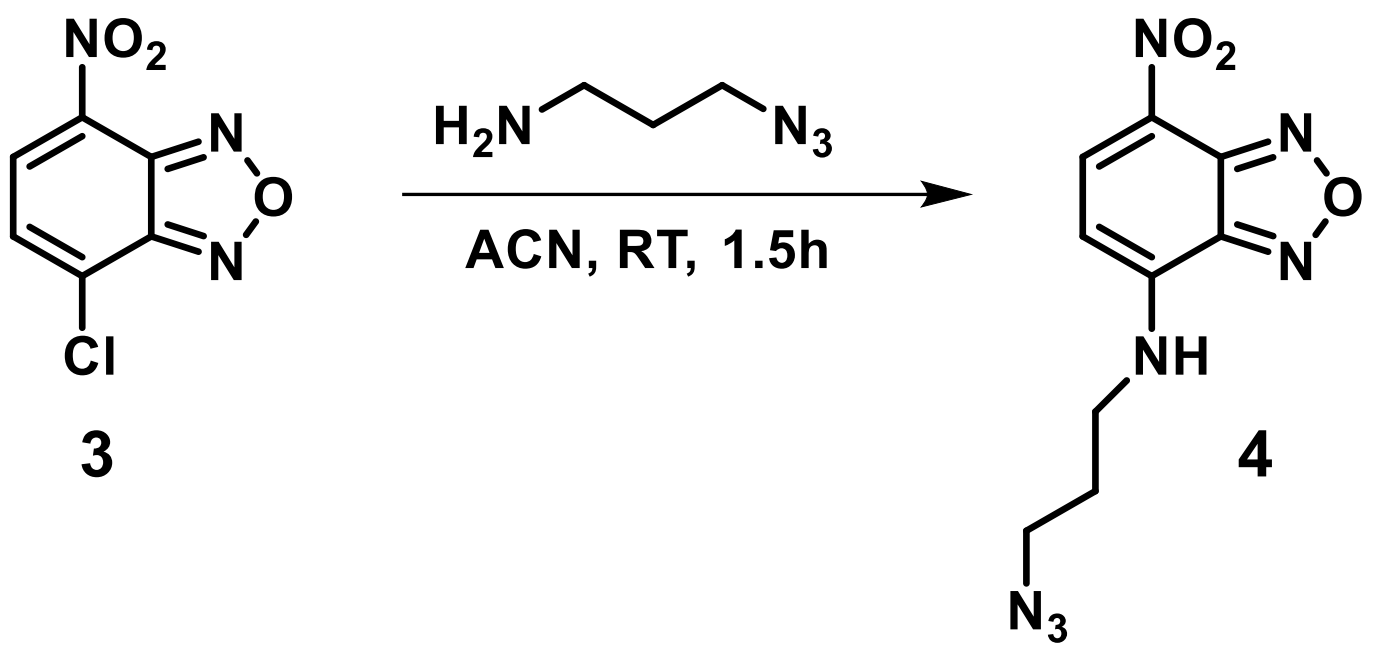

Scheme SI 2: N-(3-Azidopropyl)-7-nitro-2,1,3-benzoxadiazol-4-amine synthesis.

4-chloro-7-nitrobenzofurazan (80 mg, 4 µmol) and 3-azido-1-propanamine (44 mg, 4.4 µmol) were added to a round flask balloon, then 20 mL of acetonitrile was incorporated into the reaction mixture. The reaction was stirred at room temperature for 2 hours. The reaction was monitored by TLC using dichloromethane (DCM) as the mobile phase. The product was obtained after purification by silica chromatography in DCM. A brown powder was obtained with a molar yield 57%.

^{1}H NMR (300MHz, $CDCl_3$, δ, ppm): 8.53 (d, J = 8.6, 0.5 Hz 1H, CH_{arom}), 6.42 (s, 1H, NH), 6.24 (d, J = 8.6 Hz 1H, CH_{arom}), 3.63 (m, 4H, 2 x CH_2), 2.10 (tdd, J = 6.5, 5.7 Hz, 2H, CH_2).

^{13}C NMR (300MHz, $CDCl_3$ δ, ppm): 144.4, 143.9, 143.7, 136.4,124.7, 98.8, 49.2, 41.7, 27.9.

HRMS (ESI): m/z $[M+Na]^+$ calcd for $C_9H_9N_7O_3Na$ 286.07g/mol; found 286.06g/mol.
Rf=0.33 (100% DCM).

ε_{NBD}^{max} = 20000$M^{-1}cm^{-1}$ in propargyl alcohol.

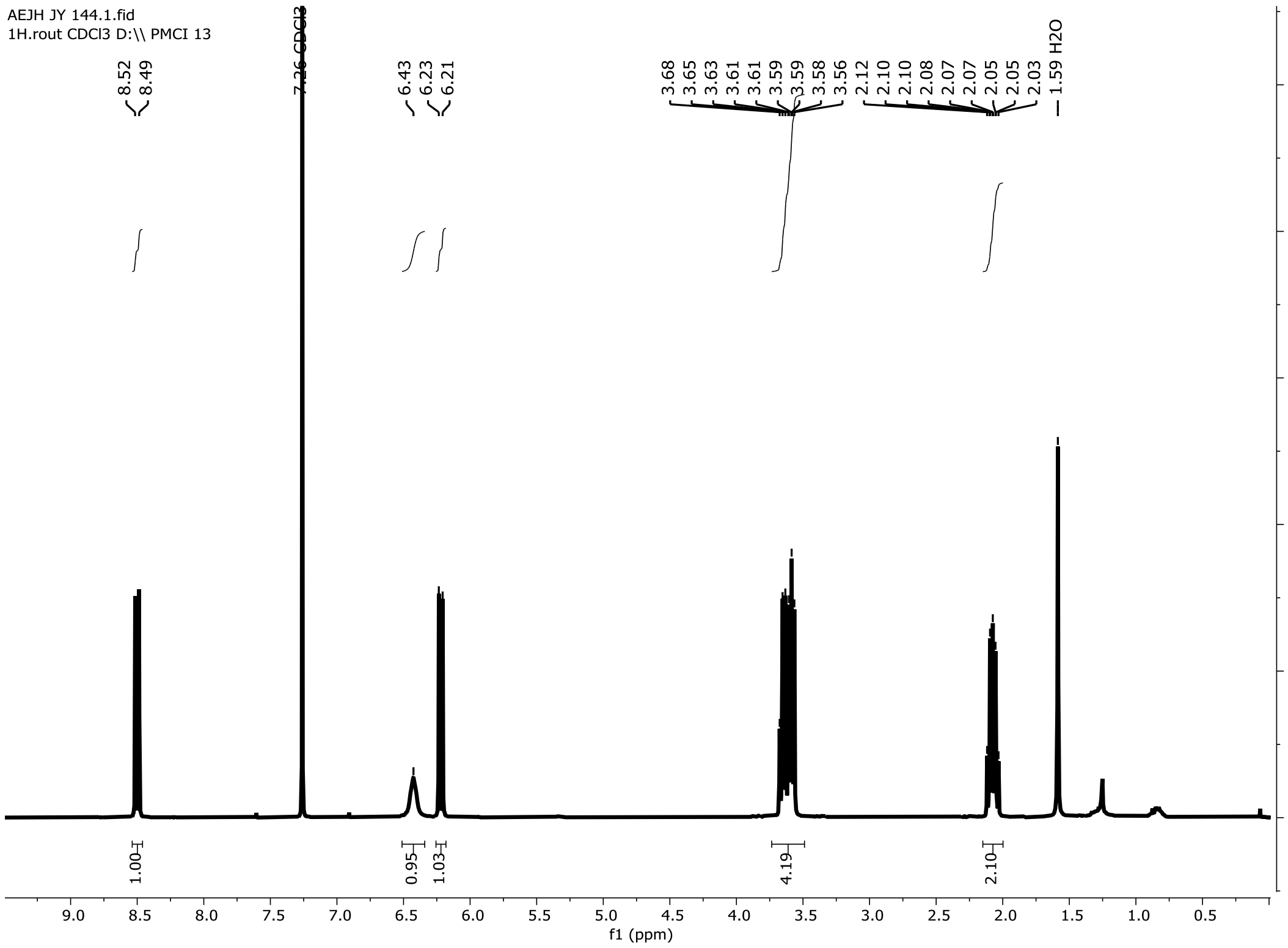

Figure SI 4: ^{1}H NMR of N-(3-Azidopropyl)-7-nitro-2,1,3-benzoxadiazol-4-amine in $CDCl_3$.

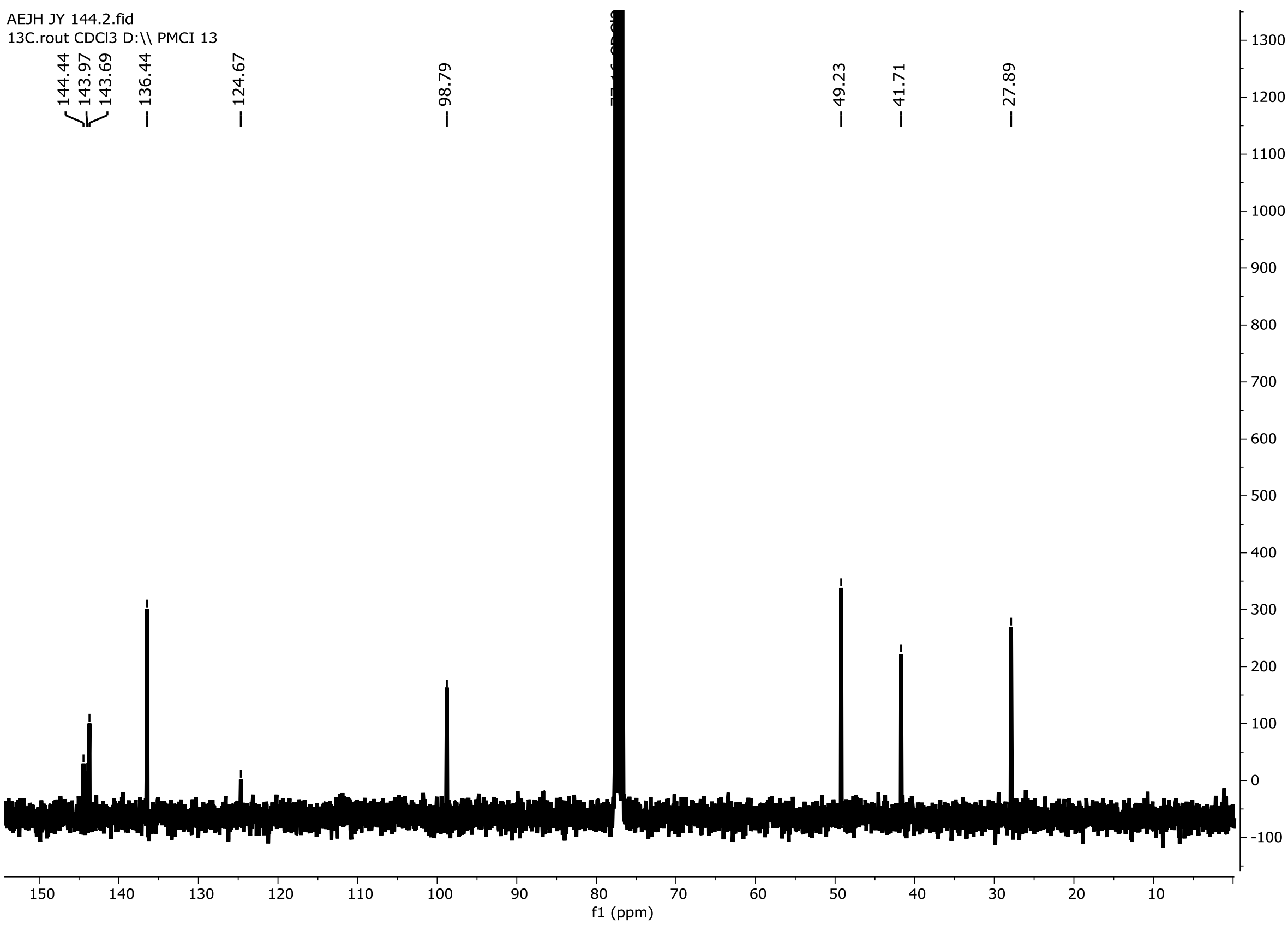

Figure SI 5: ^{13}C NMR of N-*(3-Azidopropyl)-7-nitro-2,1,3-benzoxadiazol-4-amine in $CDCl_3$.*

4.3. Ring Opening Polymerization of PLG-NCA

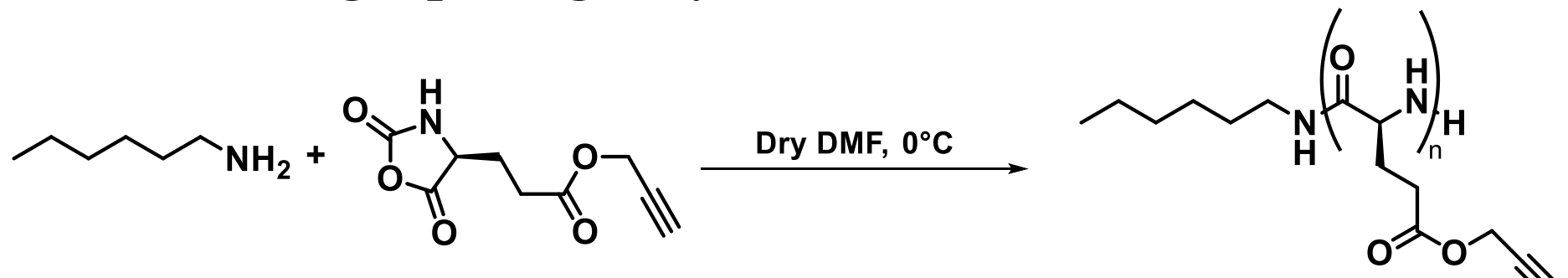

NCA-PLG PPLG

Scheme SI 3: Ring opening polymerization of propargyl-L-glutamate N-carboxyanhydride PLG-NCA initiated by hexylamine.

In a Schlenk under argon, PLG-NCA (150 mg, 0.7 mmol) was added to 2 mL of dry DMF. Then, freshly distilled hexylamine (3.1 µL, 23.7 µmol) was added to the PLG-NCA. The reaction mixture was stirred in a cold bath at 0 °C for 2 hours and then at room temperature for 24 hours. FTIR monitoring revealed the disappearance of the bands at 1787 cm^{-1} and 1854 cm^{-1}. The polymer was finally obtained by precipitation in ether as a yellowish powder and dried under vacuum. Mass **yield=** 78 %. SEC analysis indicates M_n=9.9 $Kg.mol^{-1}$ $Đ$=1.3.

^{1}H NMR (400MHz, $CDCl_3$ + 15% TFA, δ, ppm): 4.72-4.69 (m, 70H, $HCCH_2O$), 4.09-4.08 (m, 20H, NHCHCO), 2.55-2.48 (m, 95H, CH_2CH_2CO + $HCCH_2O$), 2.21-2.03 (m, 62H, CH_2CH_2CO), 1.58 (s, 2H, CH_2, Hexylamine), 1.32-1.27 (m, 6H, 3*CH_2, hexylamine), 0.89-0.86 (t, 3H, CH_3, hexylamine).

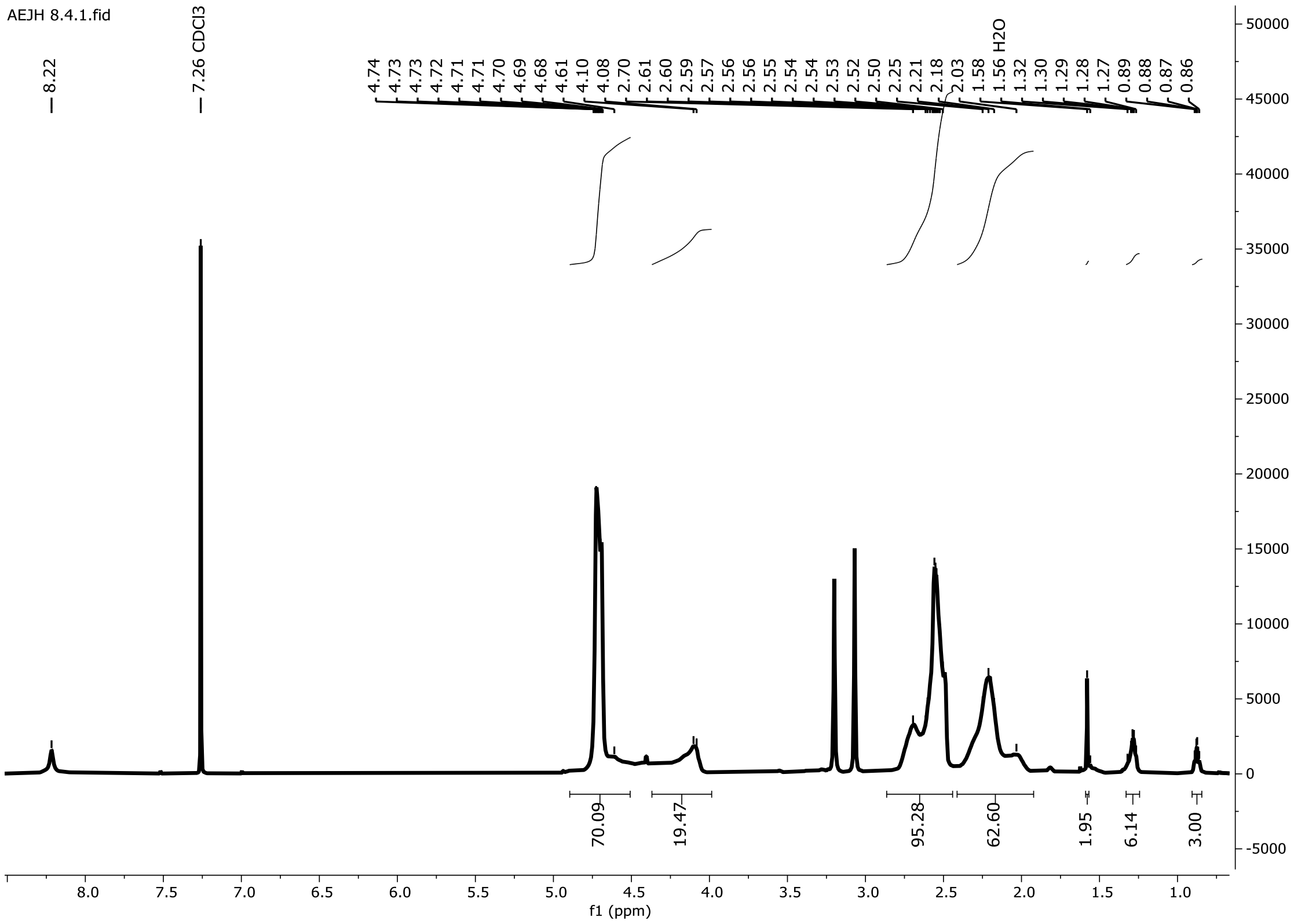

Figure SI 6: ^{1}H NMR of Hex-PPLG in $CDCl_3$ + TFA.

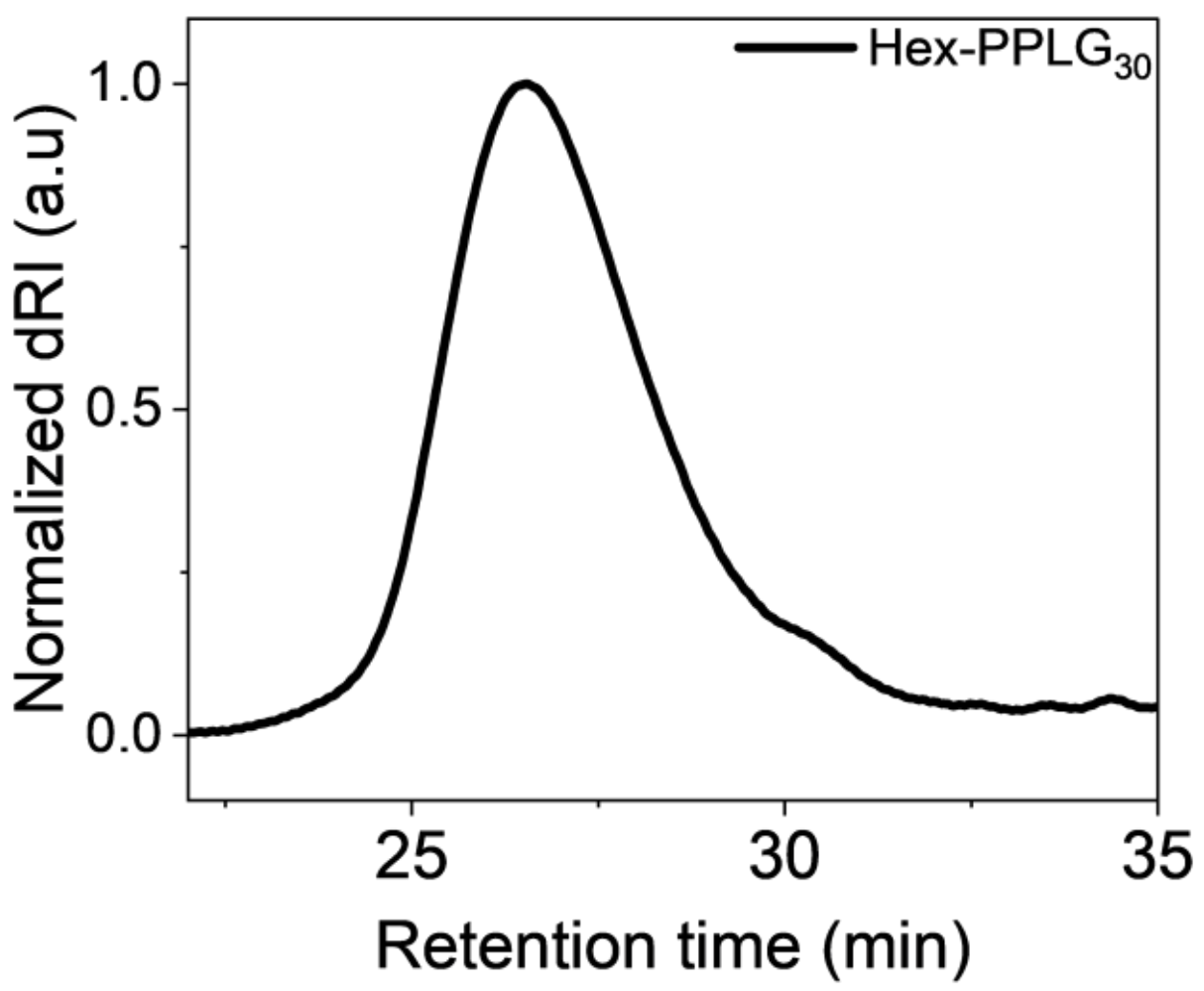

Figure SI 7: Size-exclusion chromatogram of hex-PPLG$_{30}$ in DMF (+LiBr 1mg.mL^{-1}).

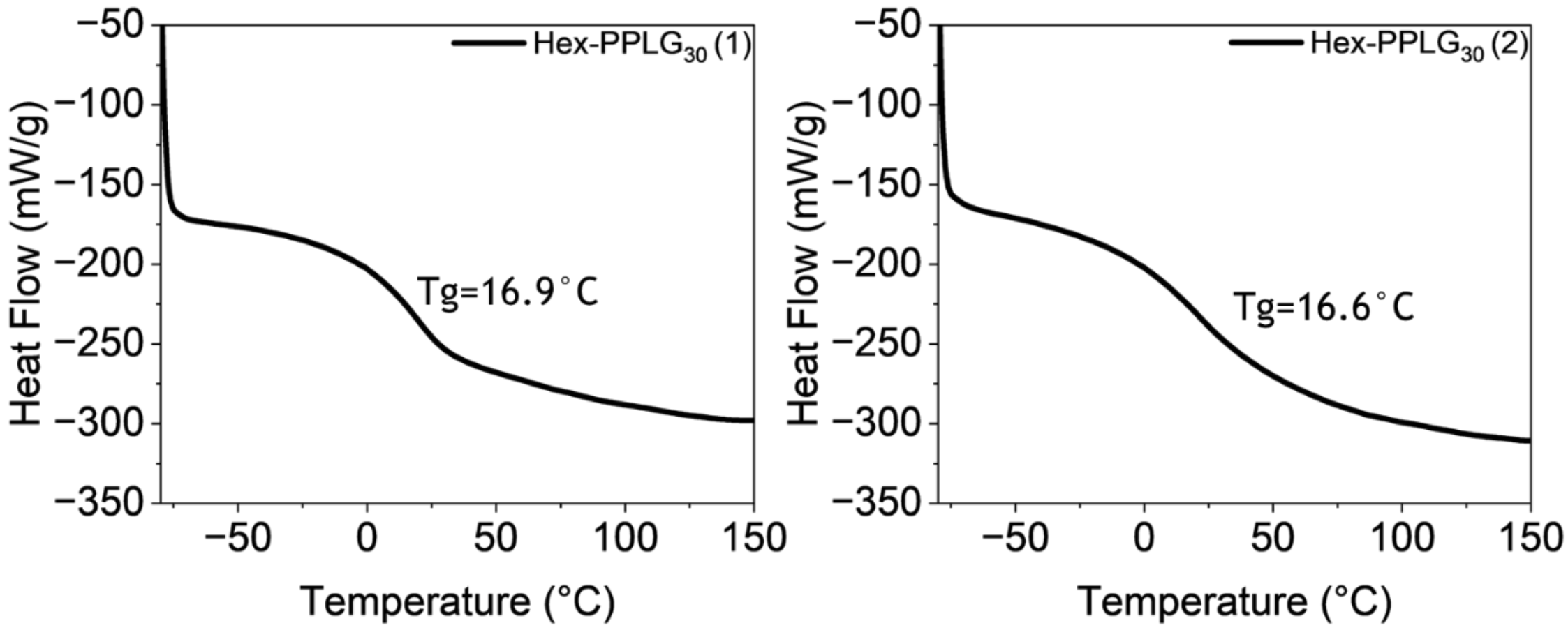

Figure SI 8: Thermogravimetric analysis of homopolymer of PPLG using hexylamine as initiator. Both curves correspond to the third heating step, since the two other heating steps allowed the solvent to be removed.

4.4. Synthesis of nanoparticles (NPs) by ROPISA

4.4.1. Synthesis of PLG-NPs

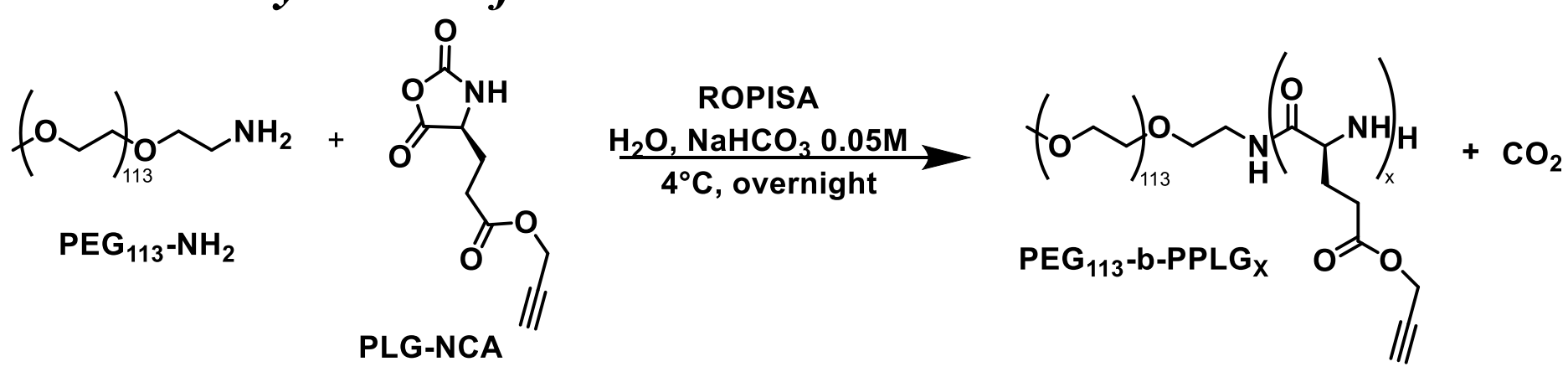

Scheme SI 4: ROPISA of PLG-NCA initiated by a PEG-NH$_2$.

ROPISA protocol:

In a glove box, PLG-NCA (32.1 mg, 0.15 mmol, 20 eq.) was weight into 2 mL microcentrifuge tube. Then, the tube was removed from the glove box and cooled in an ice bath. A solution of $NaHCO_3$ (0.05 M, pH = 8.5, 0.93 mL) containing the initiator PEG$_{113}$-NH$_2$ (38 mg, 7.6 µmol, [M]/[I] = 20) was added to the PLG-NCA. The mixture was left under strong agitation (solid content 7%): the reaction was stirred first in a cold bath with ultrasounds and then at room temperature overnight with a magnetic stirrer. A yellow opalescent dispersion was obtained. The dispersion was transferred to a 3.5 kDa dialysis membrane and dialyzed against ultrapure

water for 2 days. An aliquot was taken for structural analyses of the NPs (TEM, DLS), and the rest of the sample was freeze-dried. A yellow powder was obtained with a mass yield of 88%. SEC analysis indicates M_n=8.8 Kg.mol^{-1} $Đ$=1.1.

^{1}H NMR (400 MHz, DMSO-d$_6$, δ, ppm): 8.11 (s, 20H, NH), 4.67 (d, 34H, CH$_2$), 4.34 – 4.03 (m, 21H, CH), 3.51 (s, 452H, 2*CH$_2$ (PEG)), 3.24 (s, 3H, CH$_3$ (PEG)), 2.37 (d, 24H, CH), 2.08 – 1.70 (m, 40H).

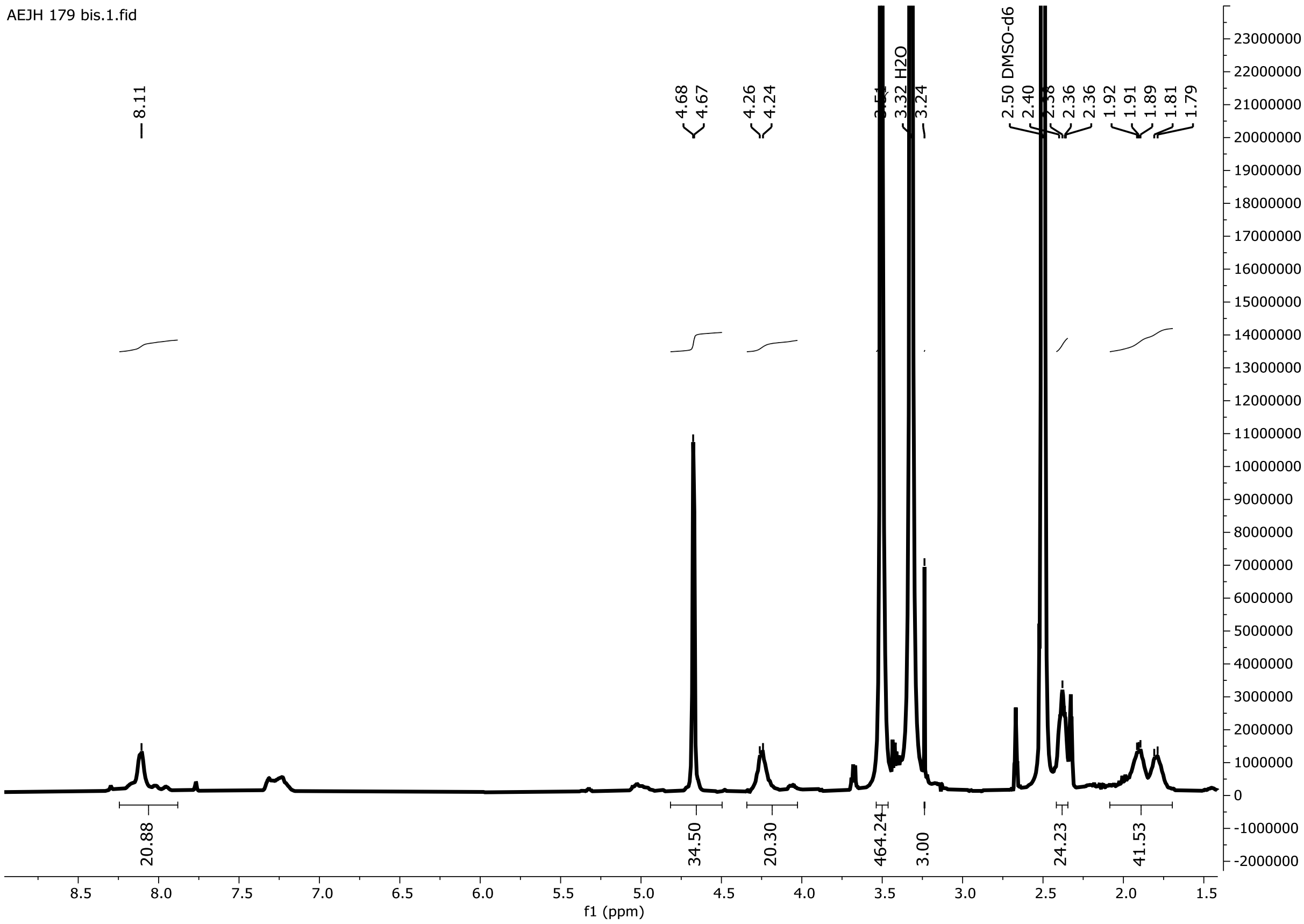

Figure SI 9: ^{1}H NMR of PEG$_{113}$-b-PPLG$_x$ in DMSO-d6.

ROPISA kinetic study:

The polymerization of PLG-NCA was initiated by PEG-NH$_2$ in the ROPISA conditions given above (aqueous NaHCO$_3$ solution) and the chain growth was followed by SEC. The PLG-NCA (50.6 mg, 0.240 mmol) was placed in a microcentrifuge tube that was then cooled in an ultrasonic bath filled with ice. A solution of NaHCO$_3$ (0.05 M, pH = 8.5, 1.5 mL) containing the initiator PEG$_{113}$-NH$_2$ (59.4 mg, 0.012 mmol) was quickly added at time 0, and the reaction was performed with ultrasounds agitation (80 W). The reaction was monitored using an HCl-quenching protocol, as previously reported.[15] At specific time intervals, 100 µL of the reaction mixture was quenched with 20 µL of 15 wt.% HCl. The sample was then freeze-dried and analyzed by SEC in DMF to monitor the increase in molar mass over time.

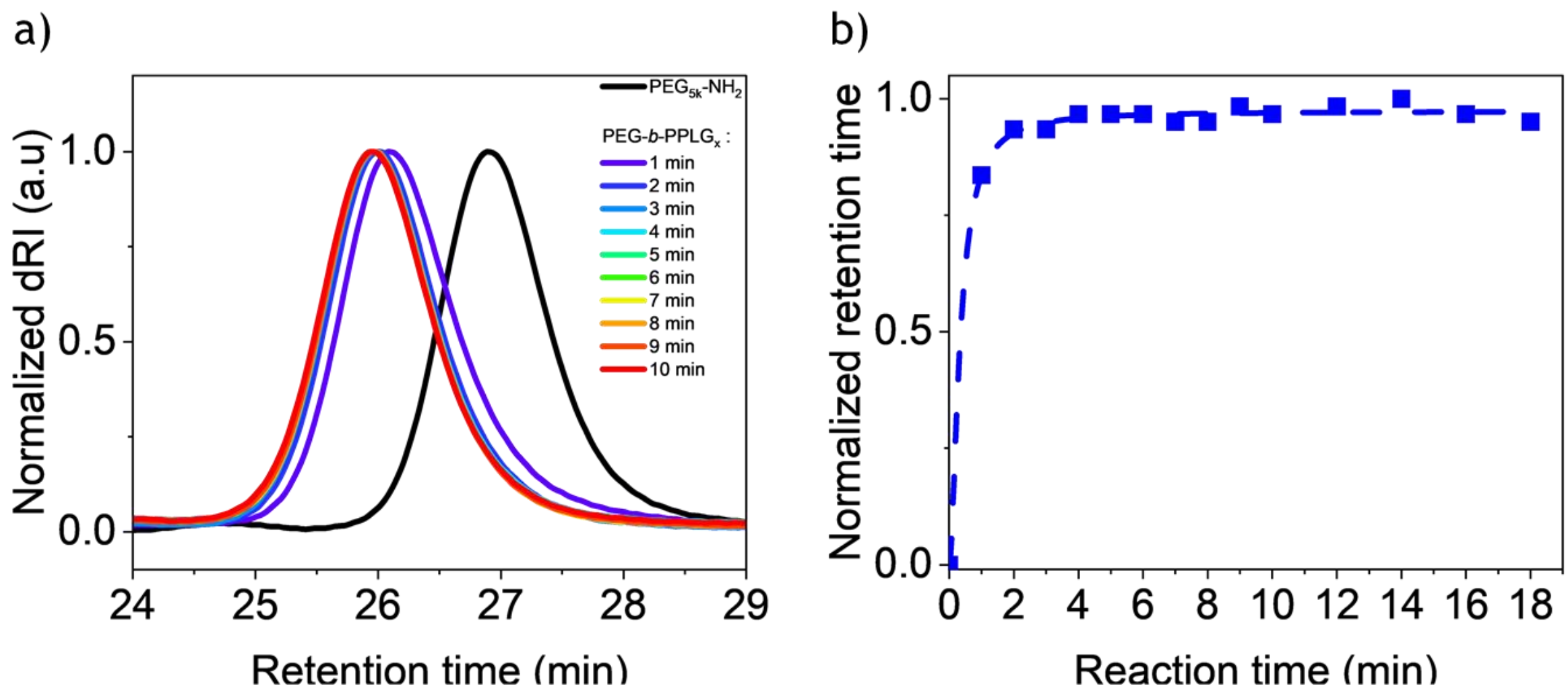

Figure SI 10: ROPISA of PLG-NCA imitated by PEG_{113}-NH2 ([M]/[I] = 20) (a) Size exclusion chromatograms in DMF (+LiBr 1mg.mL^{-1}), RI detection. b) Evolution of the retention time (SEC) in function of the reaction time, normalized using the final retention time.

ROPISA-CuAAC kinetic study:

The same kinetic protocol (described in the previous paragraph) was used to monitor the ring-opening polymerization under ROPISA-CuAAC conditions. In brief, PLG-NCA (43.7 mg, 0.207 mmol) was placed in a microcentrifuge tube that was then cooled in an ultrasonic ice bath. A solution containing the initiator PEG-NH_2 (51.6 mg, 0.010 mmol), $CuSO_4$ (0.26 mg, 1 µmol, 0.05 eq) and sodium ascorbate (2.07 mg, 10.4 µmol, 0.5 eq) in $NaHCO_3$ (1.3 mL) was quickly added to the tube at time 0. The reaction was performed under ultrasounds.

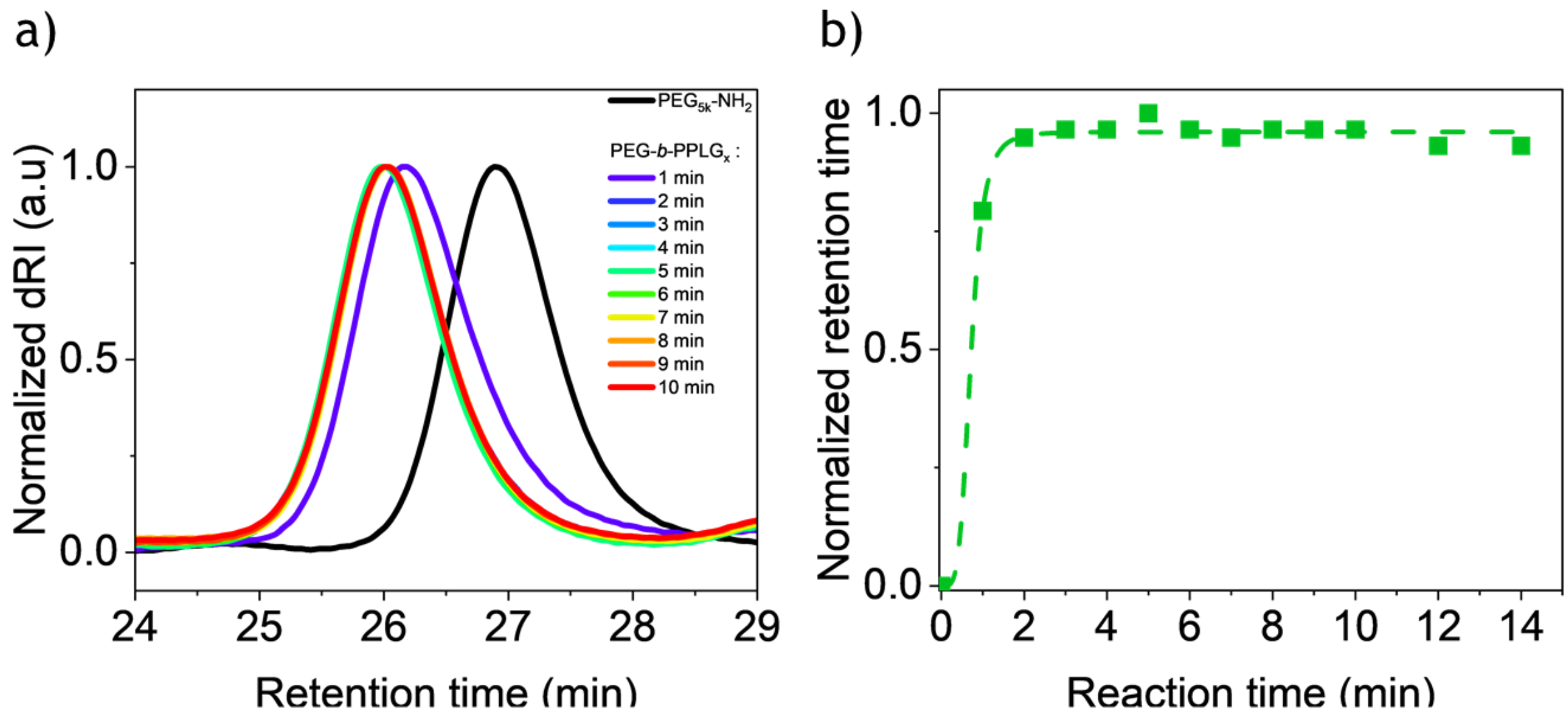

Figure SI 11: ROPISA-CuAAC of PLG-NCA imitated by PEG_{113}-NH2 ([M]/[I] = 20) a) Size exclusion chromatograms in DMF (+LiBr 1mg.mL^{-1}), RI detection. b) Evolution of the retention time (SEC) in function of the reaction time, normalized using the final retention time.

4.4.2. Synthesis of blue NPs using Cou-N_3

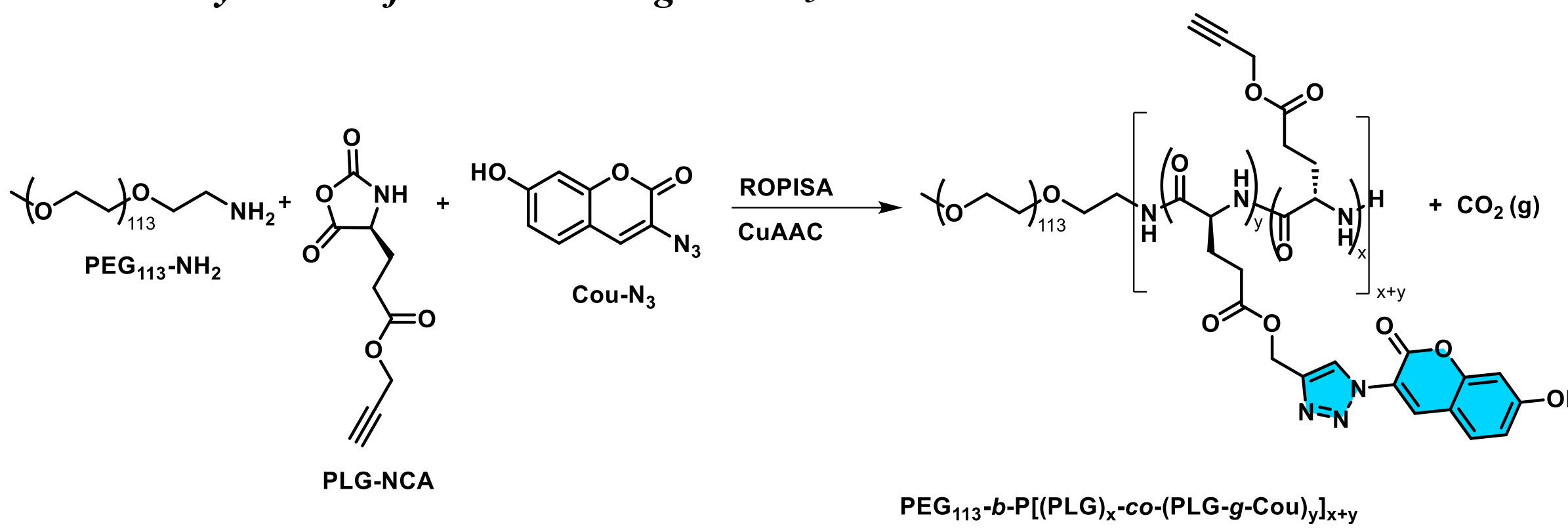

Scheme SI 5: ROPISA-CuAAC of PLG-NCA and cou-N_3 initiated by PEG-NH_2.

In a glove box, PLG-NCA (10.0 mg, 47 µmol, 20 eq.) was weighted into a 2 mL microcentrifuge tube. Then, the tube was removed from the glove box and cooled in an ice bath. A solution of $NaHCO_3$ (0.05 M, pH = 8.5, 0.3 mL) containing the initiator PEG_{113}-NH_2 (11.8 mg, 2.4 µmol, [M]/[I] = 20), 3-azido-7-hydroxycoumarin (0.5 mg, 2.4 µmol, 5 mol %), sodium ascorbate (0.23 mg, 1.2 µmol, 0.5 eq) and copper sulfate (0.03 mg, 0.12 µmol, 0.05 eq) was added to the PLG-NCA. The mixture was left under strong agitation (solid content 7%): the reaction was stirred first in a cold bath with ultrasounds and then at room temperature overnight with a magnetic stirrer. A yellow/brown opalescent dispersion was obtained. The dispersion was transferred to a 3.5-kDa dialysis membrane and dialyzed against ultrapure water containing 10 mg/L ethylenediaminetetraacetic acid (EDTA) for the first day, followed by ultrapure water for 2 days. An aliquot was taken for structural analyses of the NPs (TEM, DLS), and the rest of the sample was freeze-dried. A brown powder was obtained with a mass yield of 81%. SEC analysis indicates M_n=7.7 Kg.mol^{-1} $Đ$=1.1.

^{1}H NMR (400 MHz, DMSO-d$_6$, δ, ppm): 8.58-8.52 (m, 1H, $H_{triazole\ ring}$), 8.21-7.95 (m, 17H, NH), 7.74 (d, 2H, 2*H_{arom} (Cou)), 6.93 – 6.76 (m, 2H, 2*H_{arom} (Cou)), 4.67 (d, 33H, CH_2), 4.38 – 4.00 (m, 20H, CH), 3.51 (s, 452H, 2*CH_2 (PEG)), 3.24 (s, 3H, CH_3 (PEG)), 2.39 (m, 32H, CH+CH_2), 2.13 – 1.59 (m, 38H, CH_2).

ICP-OES: Cu (mg.g^{-1}) < 0.06.

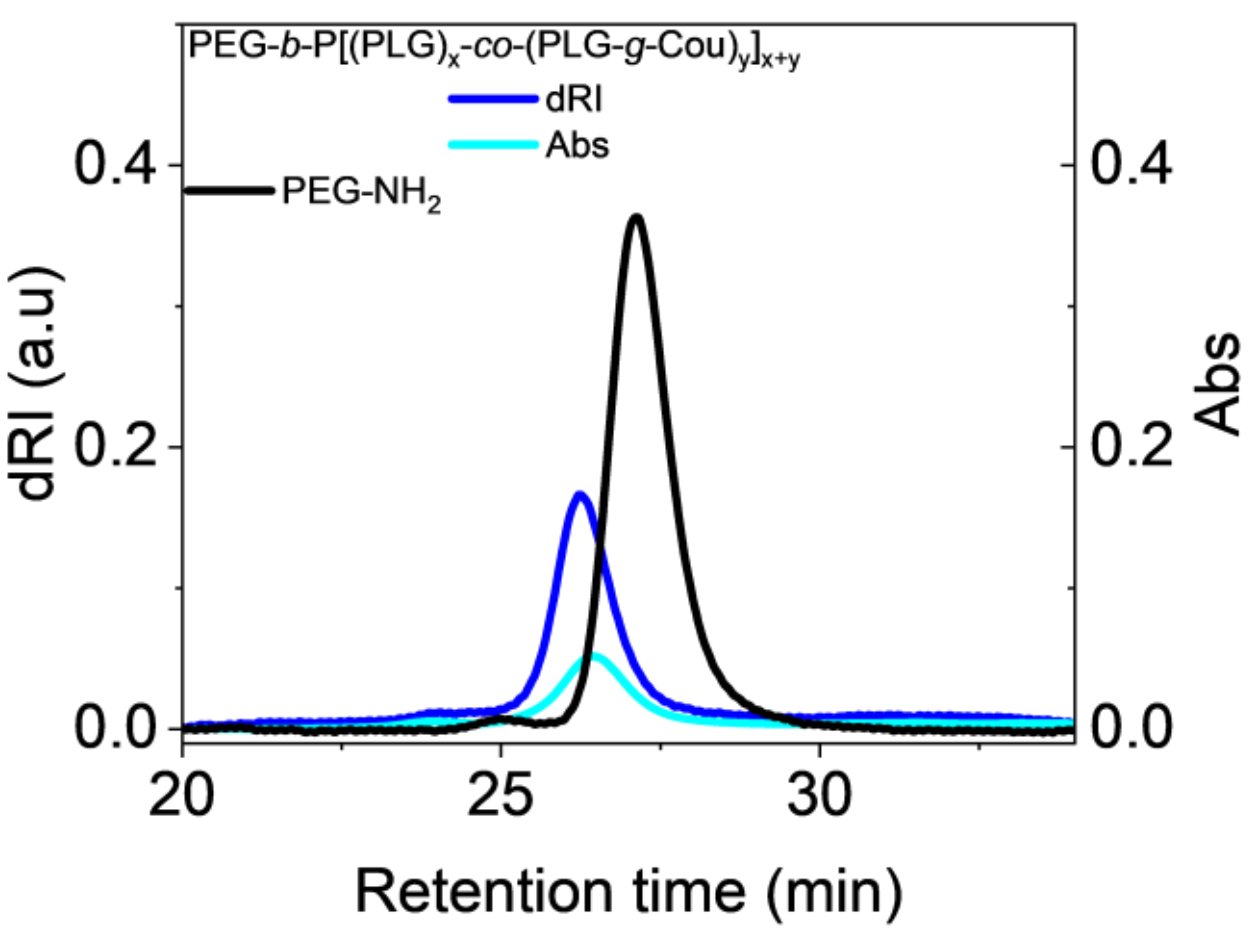

Figure SI 12: Size exclusion chromatograms in DMF (+LiBr 1mg.mL^{-1}), RI detection, Abs detector.

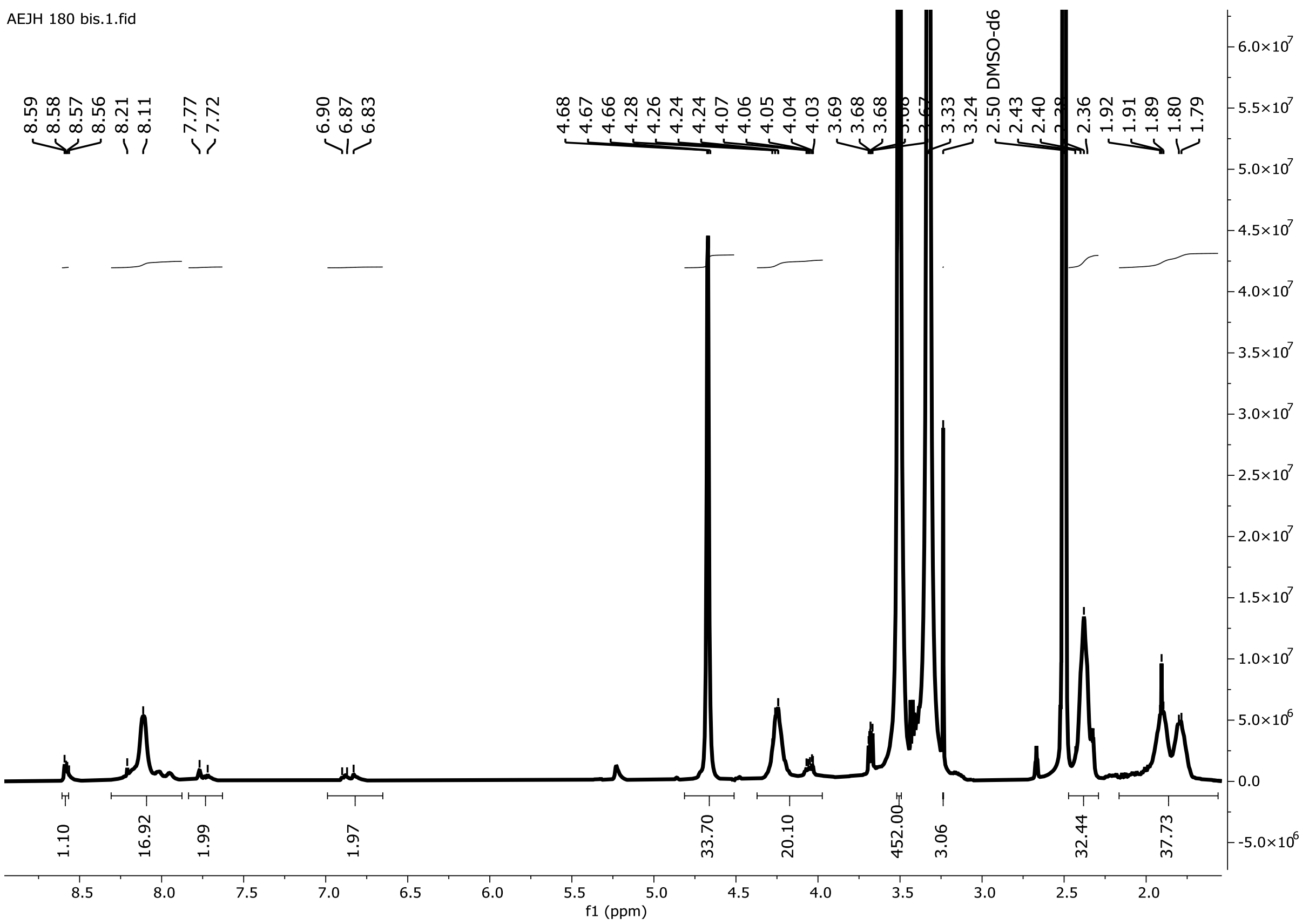

Figure SI 13: ^{1}H NMR of PEG$_{113}$-b-P[(PLG)$_x$-co-(PLG-g-Cou)$_y$]$_{x+y}$ in DMSO-d$_6$.

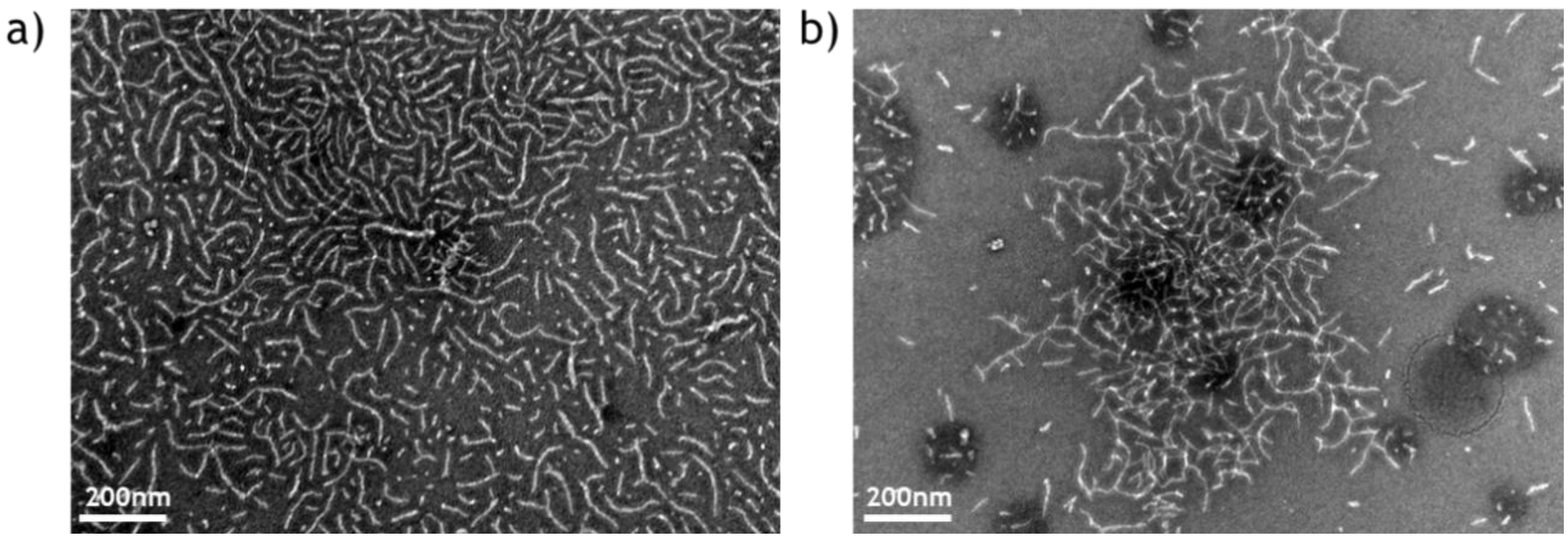

Figure SI 14: a) TEM image (scale bar = 200nm, stained using uranyl acetate) of PEG$_{113}$-b-P[(PLG)$_x$-co-(PLG-g-Cou)$_y$]$_{x+y}$ NPs synthesized by ROPISA b) TEM image (scale bar = 200nm, stained using uranyl acetate of PEG$_{113}$-b-P[(PLG)$_x$-co-(PLG-g-Cou)$_y$]$_{x+y}$ NPs synthesized by ROPISA after lyophilization and re-dispersion in water.

Click chemistry kinetic:

The speed of the CuAAC reaction was monitored using the 3-azido-7-hydroxycoumarin as a fluorogenic dye.[16] PLG-NCA (22.6 mg, 0.11 mmol) was weighted in a microcentrifuge tube inside a glove box. Then, it was removed from the glove-box and placed in an ultrasonic bath filled with ice. An aqueous solution of $NaHCO_3$ (0.7 mL), containing the initiator PEG$_{113}$-NH$_2$ (26.4 mg, 5.3 µmmol), 3-azido-7-hydroxycoumarin (2.2 mg, 11 µmol), $CuSO_4$ (0.13 mg, 1 µmol, 0.05 eq) and NaAsc (1.1 mg, 5 µmol) was added to the tube under ultrasound for 10 min.

Then, a magnetic stirring bar was added to the dispersion, and the sample was kept under magnetic agitation for 3 h at room temperature. At specific times, 20 µL of the reaction mixture was quenched with 4 µL of 15 wt.% HCl. Then, 3 µL of the sample was diluted in 1.5 mL of acetone, transferred to a quartz cuvette, and its fluorescence was recorded (λ^{exc} = 350 nm).

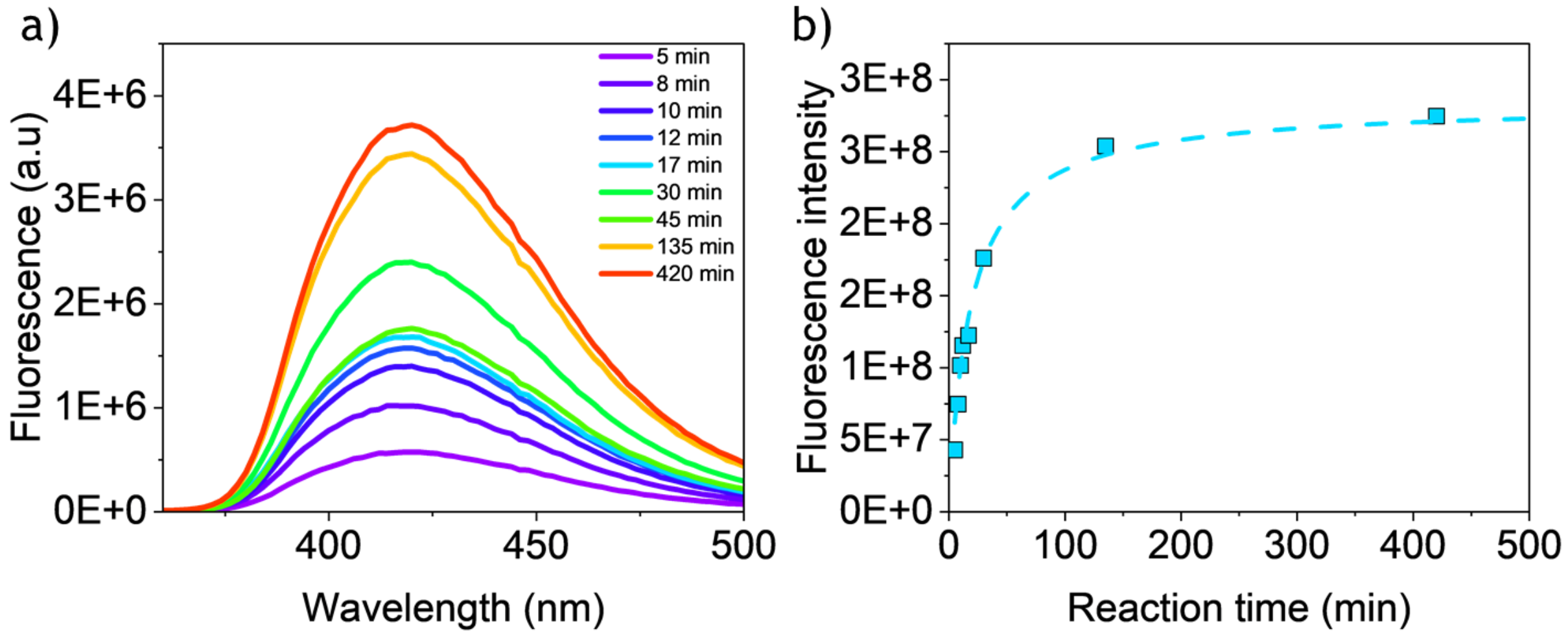

Figure SI 15: a) Fluorescence intensity evolution over time (λ^{exc}= 350 nm), of samples dispersed in acetone, containing PEG_{113}-b-P[(PLG)$_x$-co-(PLG-g-Cou)$_y$]$_{x+y}$ polymer in the ROPISA-CuAAC conditions and quenched with HCl. b) Total fluorescence intensity of the same samples in function of the reaction time.

4.4.3. Synthesis of green NPs using NBD-N_3

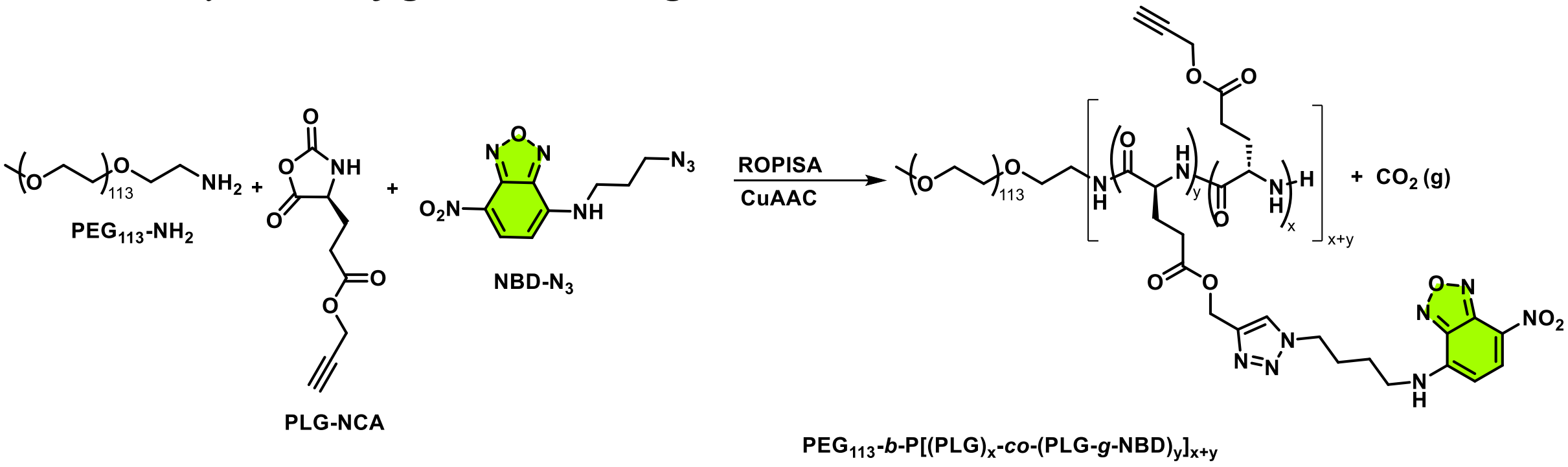

Scheme SI 6: ROPISA-CuAAC of PLG-NCA and NBD-N_3 initiated by PEG-NH_2.

In a glove box, PLG-NCA (7.0 mg, 33 µmol, 20 eq.) was weighted into a 2 mL microcentrifuge tube. Then, the tube was removed from the glove box and cooled in an ice bath. A solution of $NaHCO_3$ (0.05 M, pH = 8.5, 0.21 mL) containing the initiator PEG_{113}-NH_2 (8.3 mg, 1.7 µmol, [M]/[I] = 20), *N*-(3-azidopropyl)-7-nitro-2,1,3-benzoxadiazol-4-amine (0.4 mg, 2 µmol, 5 mol %), sodium ascorbate (0.16 mg, 1 µmol, 0.5 eq.) and copper sulfate (0.02 mg, 0.1 µmol, 0.05 eq.) was added to the PLG-NCA. The mixture was left under strong agitation (solid content 7 %): the reaction was stirred first in a cold bath with ultrasounds and then at room temperature overnight with a magnetic stirrer. A yellow, opalescent dispersion was obtained. The dispersion was transferred to a 3.5 kDa dialysis membrane and dialyzed against ultrapure water containing EDTA (10 mg/L) for the first day, followed by ultrapure water for 2 days. An aliquot was kept for structural analysis (DLS, TEM, NTA) and the rest was freeze-dried. A yellow powder was obtained with a mass yield of 83%. SEC analysis indicates M_n=9.5 Kg.mol^{-1} $Đ$=1.1.

^{1}H NMR (400 MHz, DMSO-d$_6$, δ, ppm): 8.48 (s, 1H, $H_{triazole\ ring}$), 8.20-7.95 (s, 18H, NH), 7.77 (s, 1H, H_{arom} (NBD)), 6.37 (s, 1H, H_{arom} (NBD), 4.67 (d, *J* = 2.5 Hz, 38H, CH_2), 4.24 (s, 20H, CH), 3.51 (s, 452H, 2*CH_2 (PEG)), 3.24 (s, 3H, CH_3, (PEG), 2.38 (t, 37H, CH+CH_2), 2.03 – 1.63 (m, 40H, CH_2).

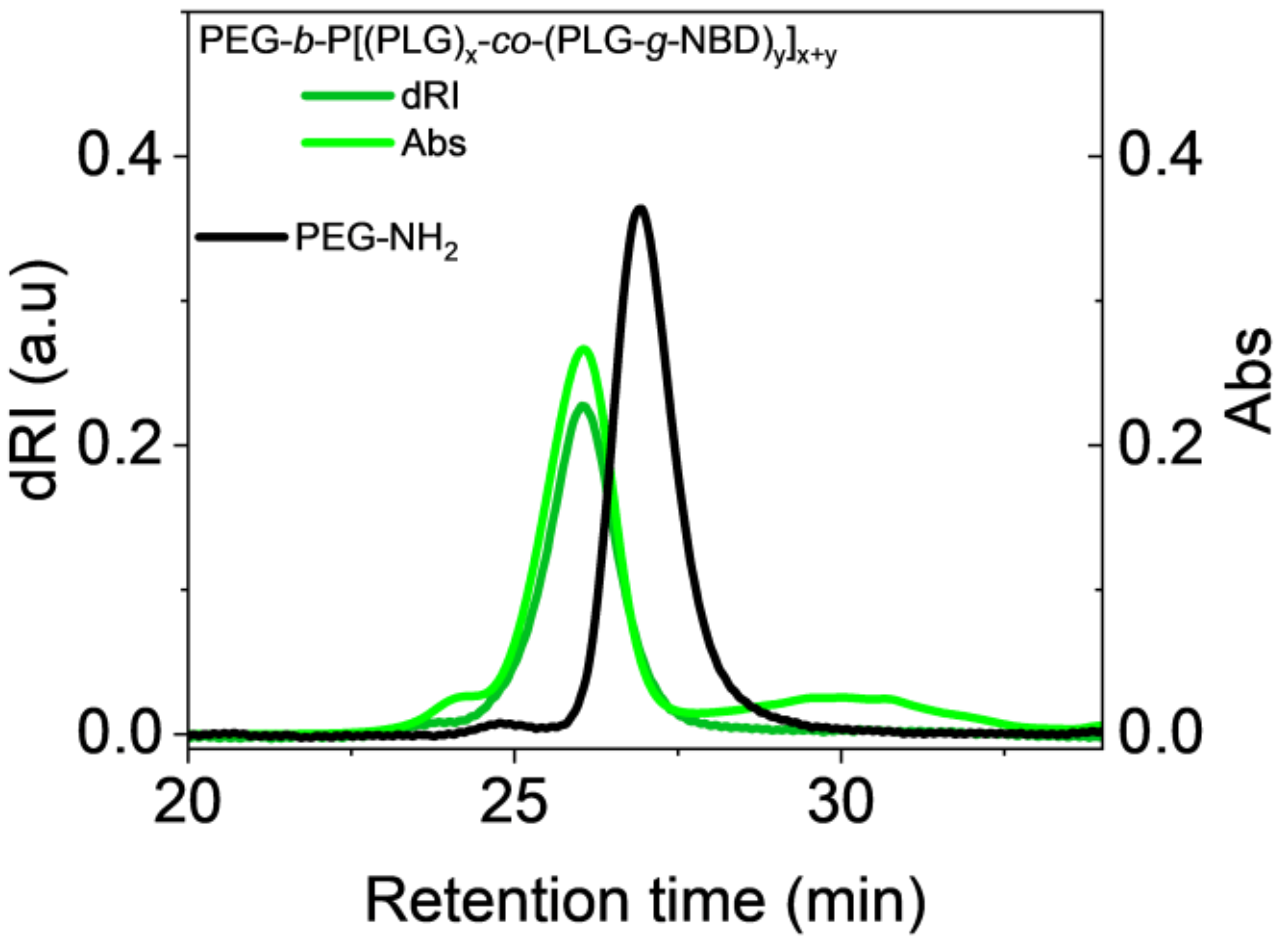

Figure SI 16: Size exclusion chromatograms in DMF (+LiBr 8.7 mg. mL^{-1}), RI detection, Abs detector.

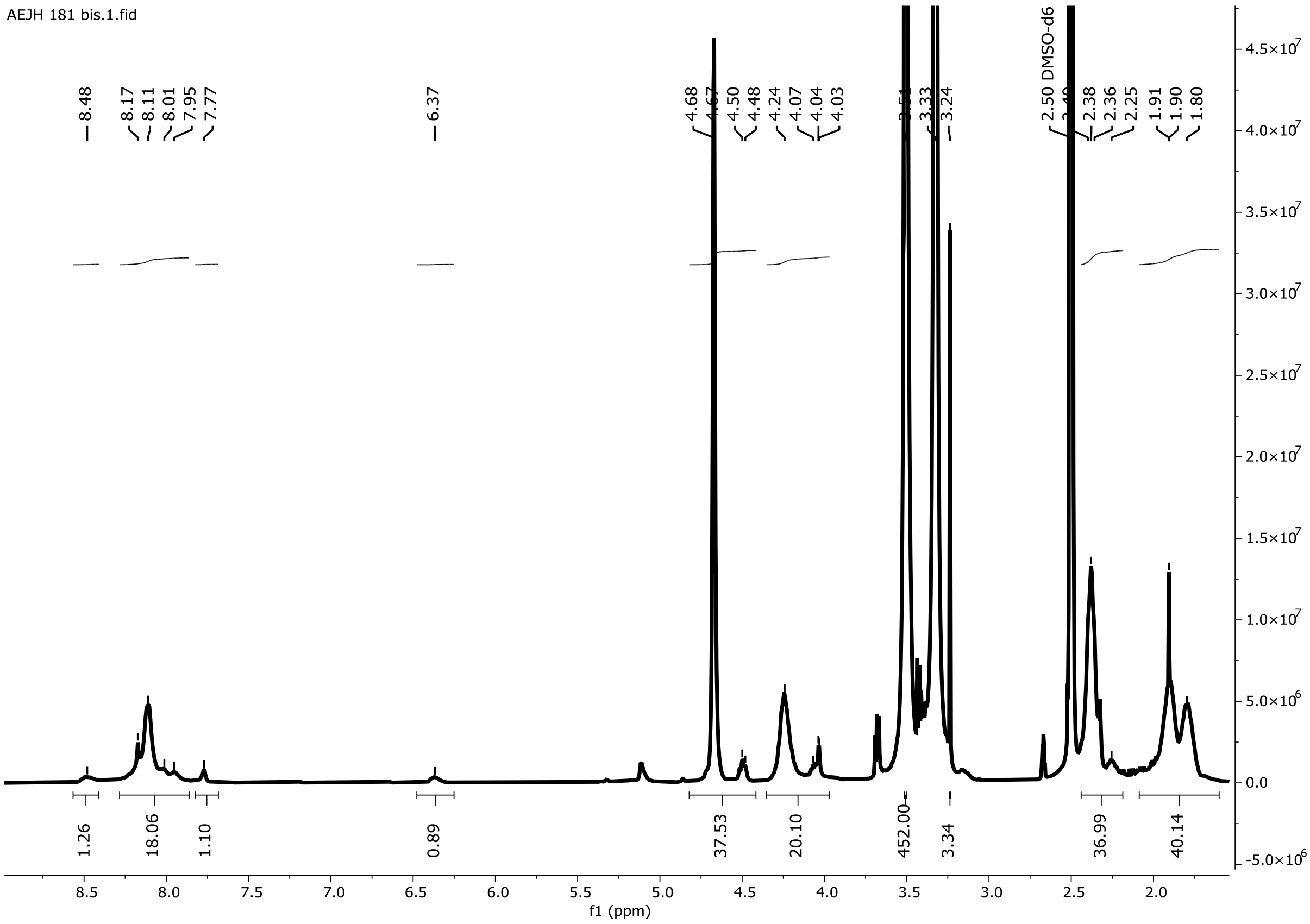

Figure SI 17: ^{1}H NMR of PEG$_{113}$-b-P[(PLG)$_x$-co-(PLG-g-NBD)$_y$]$_{x+y}$ in DMSO-d$_6$.

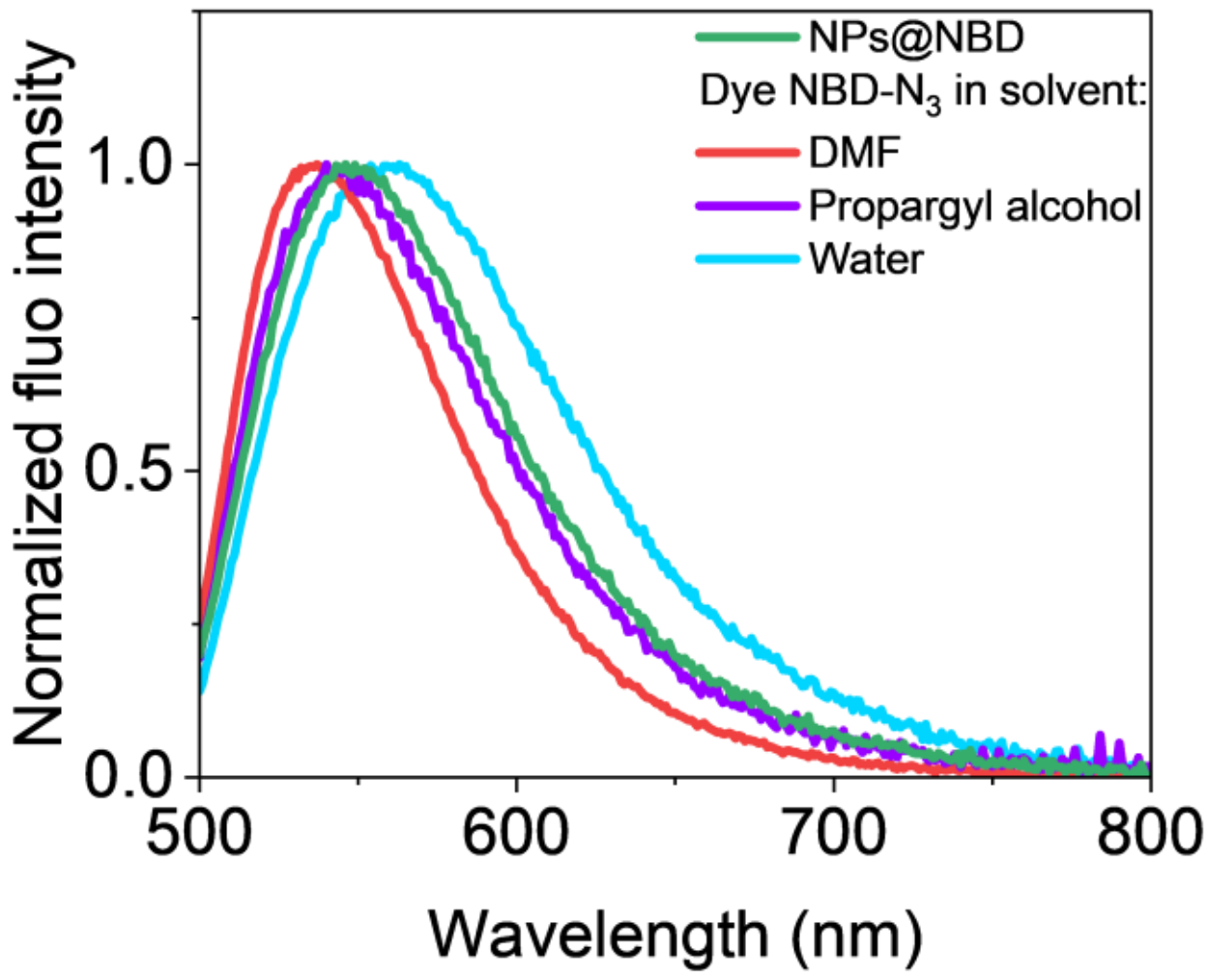

Figure SI 18: Normalized fluorescence spectra of the NBD-N_3 dye in different solvents overlapped with the one of NBD-NPs in water at λ^{exc}=470nm.

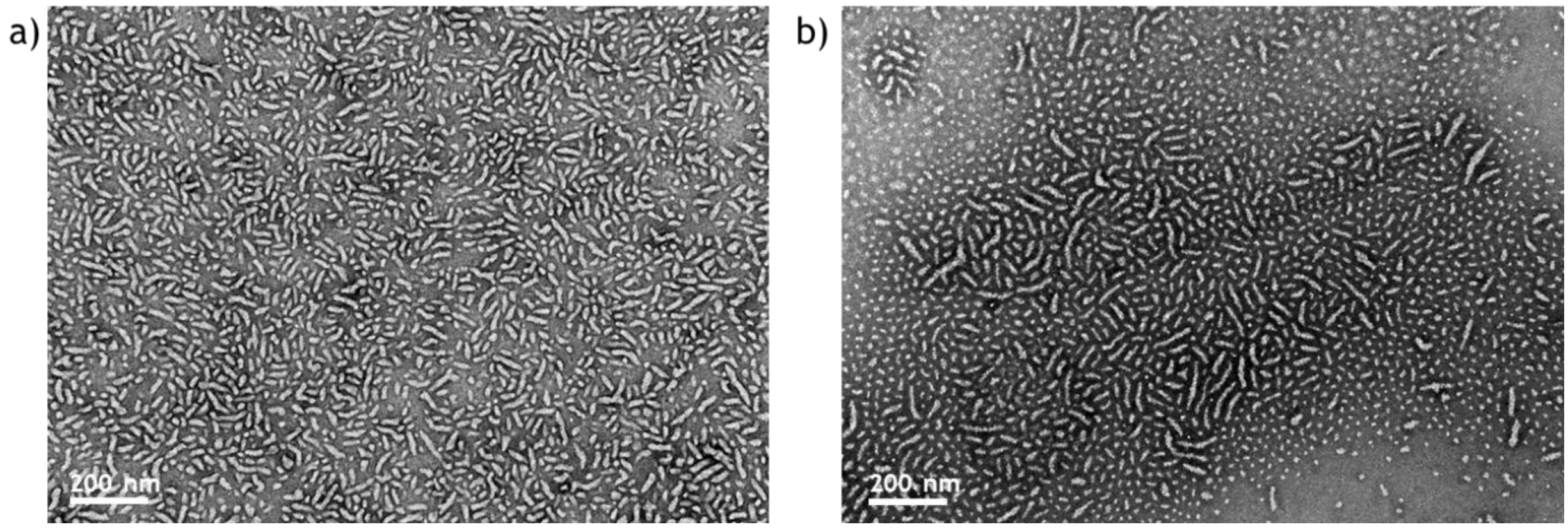

Figure SI 19: TEM images of NBD-NPs, stained using uranyl acetate. a) after ROPISA-CuAAC. b) after freeze-dried and re-suspended in ultra-pure water.

4.4.4. Synthesis of red NPs using Cy5-N_3

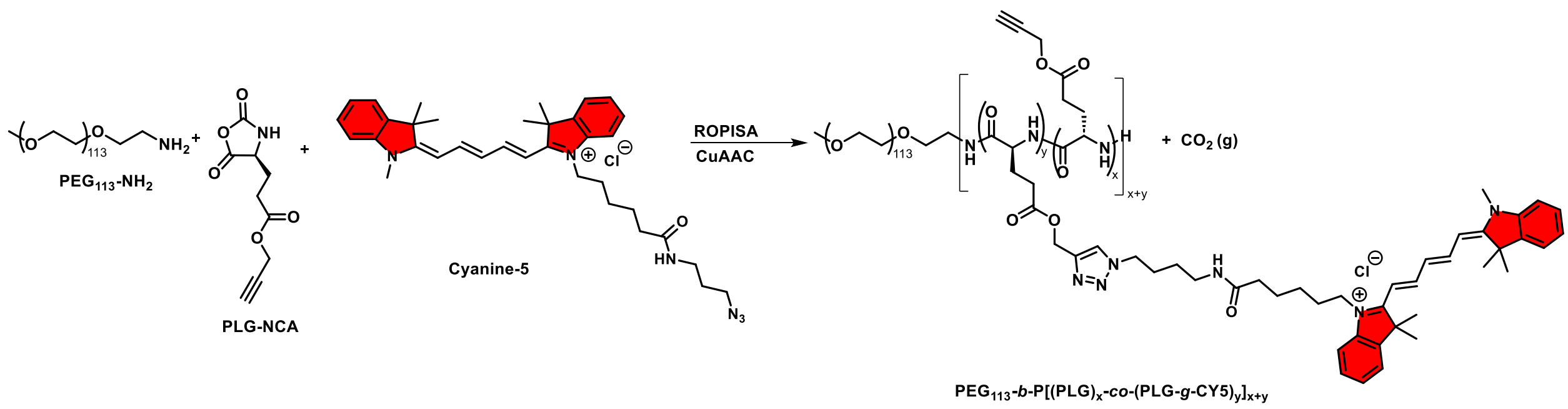

Scheme SI 7: ROPISA-CuAAC of PLG-NCA and Cy5-N_3 initiated by PEG-NH_2.

In a glove box, PLG-NCA (12.8 mg, 61 µmol, 20 eq.) was weighted into 2 mL microcentrifuge tube. Then, the tube was removed from the glove box and cooled on an ice bath. A solution of $NaHCO_3$ (0.05M, pH=8.5, 0.4 mL) containing the initiator PEG_{113}-NH_2 (15.2 mg, 3.0 µmol, [M]/[I]=20), cyanine-5 azide (1.8mg, 30µmol, 5 mol %), sodium ascorbate (0.3 mg, 1.5 µmol, 0.5 eq.) and copper sulphate (0.04mg, 0.15µmol, 0.05 eq.) was added to the PLG-NCA. The mixture was left under strong agitation (solid content 7%): the reaction was stirred first in a cold bath with ultrasounds and then at room temperature overnight with a magnetic stirrer. A

blue opalescent dispersion was obtained. The dispersion was transferred to a 3.5 kDa dialysis membrane and dialysed against ultrapure water containing EDTA (10 mg. L^{-1}) for the first day, followed by ultrapure water for 2 days. An aliquot was kept for structural analysis (DLS, TEM, NTA) and the rest was freeze-dried. A blue powder was obtained with a 79% mass yield. SEC analysis indicates M_n=8.4 Kg.mol^{-1} $Đ$=1.1.

^{1}H NMR (400 MHz, DMSO-d_6, δ, ppm): 8.45 (s, 1H, $H_{triazole}$), 8.35 (s, 2H, H_{arom} (Cy5)), 8.15-7.96 (m, 18H, NH), 7.77 (s, 2H, H_{arom}, (Cy5)), 7.60 (d, 2H, H_{Cy5}), 7.43 – 7.33 (m, 3H, H_{Cy5}), 7.24 (m, 3H, H_{Cy5}), 6.70 – 6.50 (m, 3H, H_{Cy5}), 6.28 (dd, 2H, H_{Cy5}), 5.32 (s, 2H, H_{Cy5}), 5.28 – 5.04 (m, 5H, H_{Cy5}), 4.67 (d, 36H, CH_2), 4.38 – 4.09 (m, 20H, CH), 3.70 – 3.66 (m, 3H, CH_{3Cy5}), 3.51 (s, 452H, 2*CH_2 (PEG-NH2)), 3.21 (s, 3H, CH_3 (PEG), 2.84 (s, 4H, H_{Cy5}), 2.40-2.32 (m, 38H, CH_2+CH) 1.84 (d, 40H, CH_2), 1.67 (s, 6H, H_{Cy5}), 1.23 (s, 10H, H_{Cy5}).

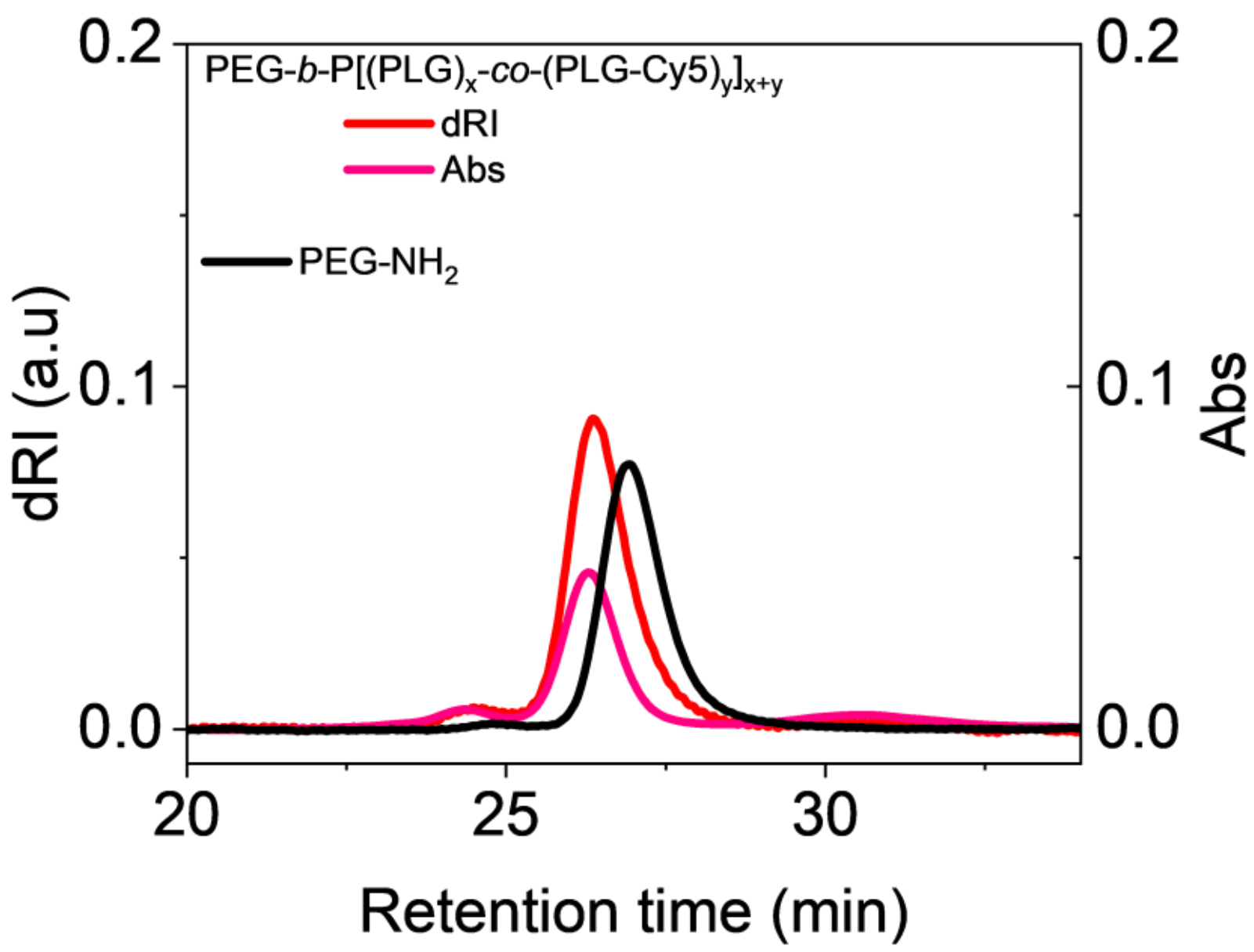

Figure SI 20: Size exclusion chromatograms in DMF (+LiBr 8.7 mg. mL^{-1}), RI detection, Abs detector.

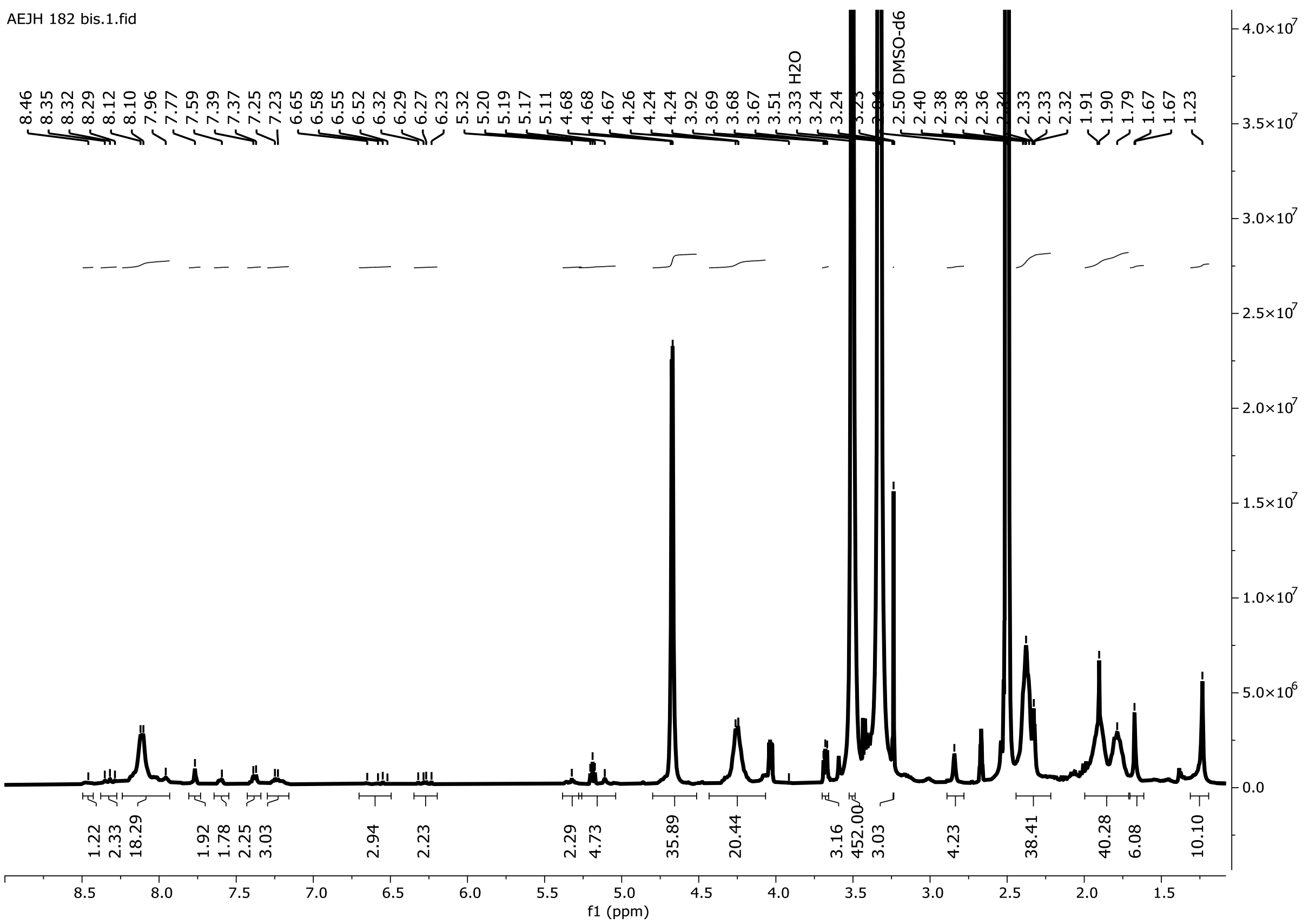

Figure SI 21: ^1H NMR PEG_{113}-b-$P[(PLG)_x$-co-$(PLG$-g-$Cy5)_y]_{x+y}$ in DMSO-d_6.

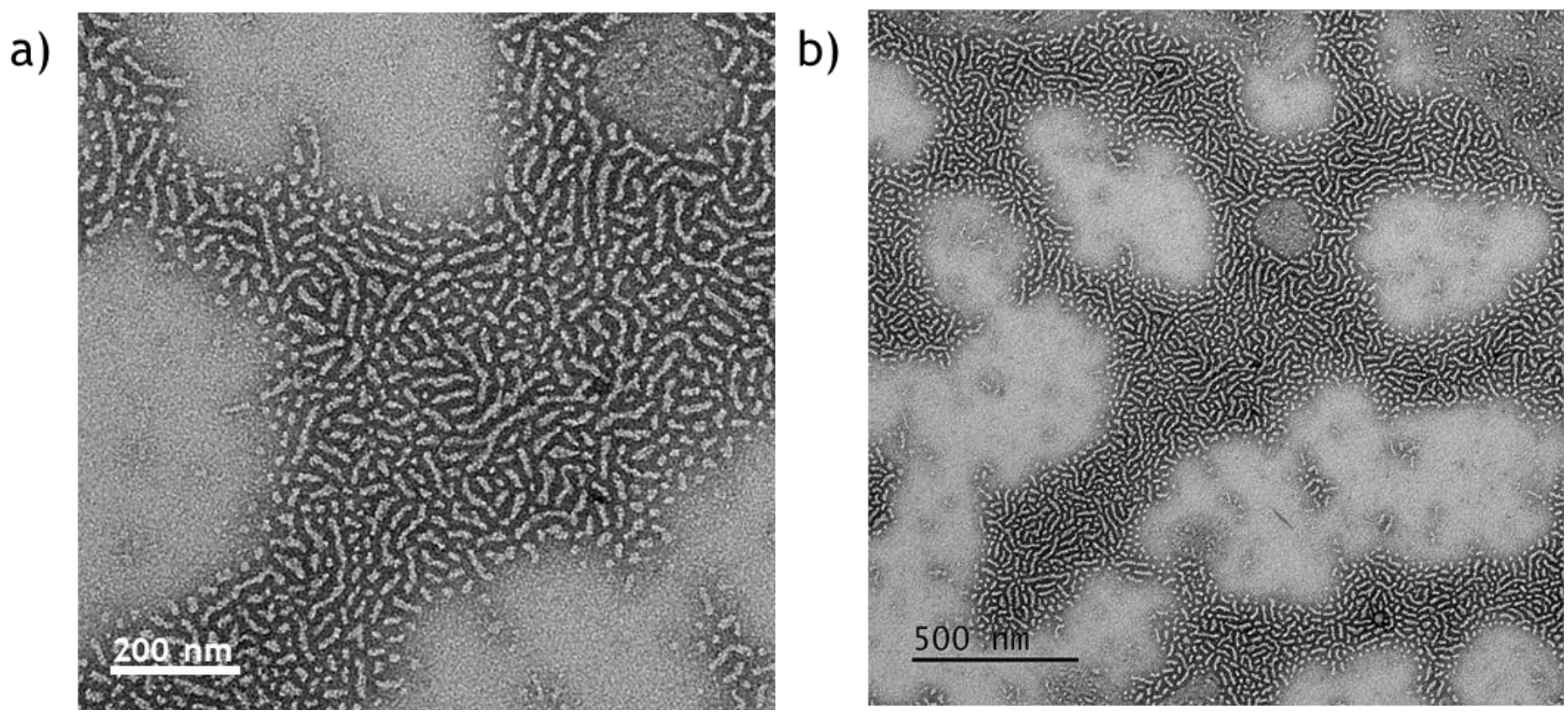

Figure SI 22: TEM images of Cy5-NPs stained using uranyl acetate. a) Right after synthesis. b) Freeze-dried NPs re-suspended in ultra-pure water.

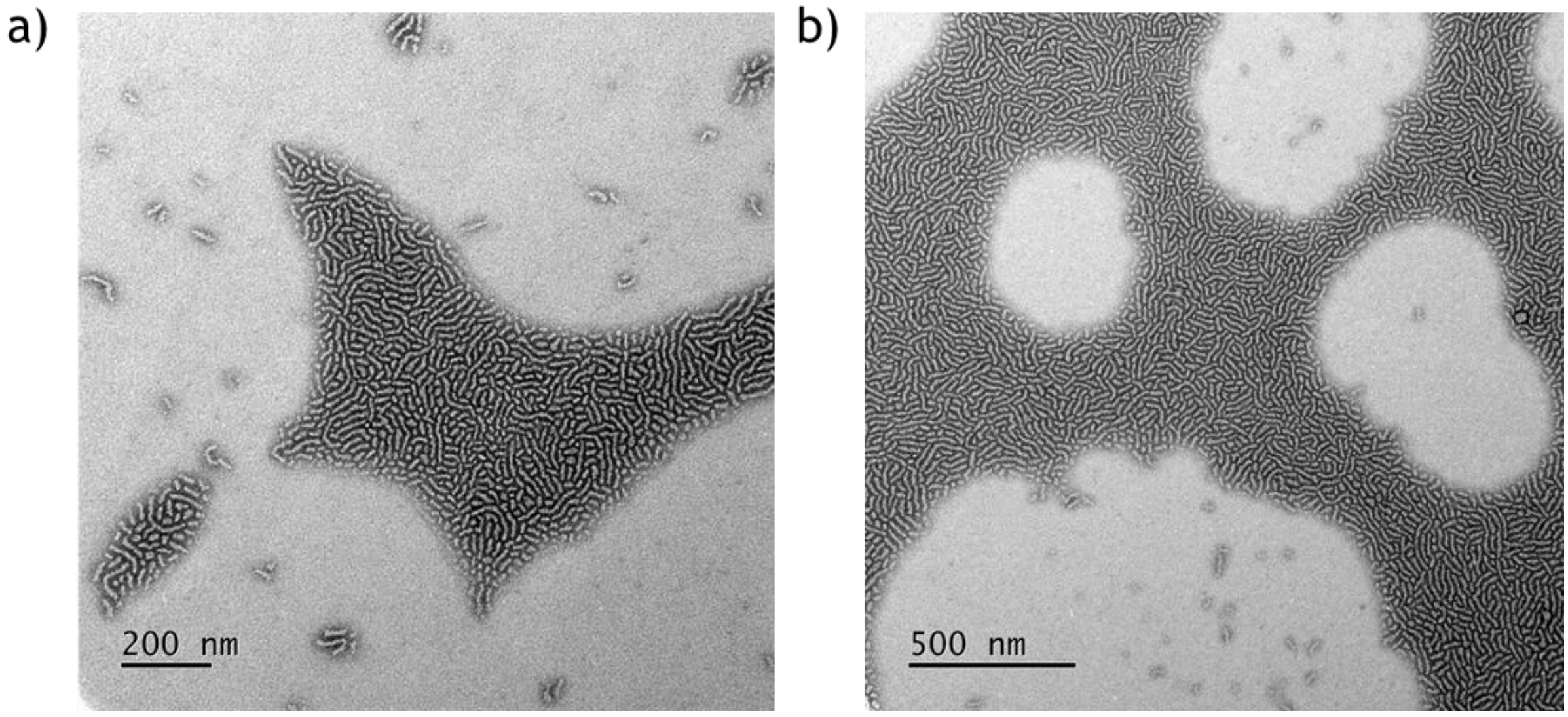

Figure SI 23: TEM images of NBD-NPs and Cy5-NPS mixed using ultrasounds, stained using uranyl acetate. a) after ultra-sounds. b) freeze-dried and re-suspended in ultra-pure water.

4.4.5. Synthesis of biotinylated NPs

In a glove box, PLG-NCA (8.3 mg, 39 µmol, 20 eq.) was weight into 2 mL microcentrifuge tube. Then, the tube was removed from the glove box and cooled on an ice bath. A solution of $NaHCO_3$ (0.05M, pH=8.5, 0.25 mL) containing the initiator PEG_{113}-NH_2 (8.9 mg, 1.8 µmol, 90 %, [M]/[I]=20) and BiotinPEG_{71}-NH_2 (0.7 mg, 0.2 µmol, 10%, [M]/[I]=20), N-(3-Azidopropyl)-7-nitro-2,1,3-benzoxadiazol-4-amine (0.5 mg, 2 µmol, 5% mol), sodium ascorbate (0.19 mg, 1 µmol, 0.5 eq.) and copper sulphate (0.02 mg, 0.1 µmol, 0.05 eq.) was added to the PLG-NCA. The mixture was left under strong agitation (solid content 7%): the reaction was stirred first in a cold bath with ultrasounds and then at room temperature overnight with a magnetic stirrer. A yellow opalescent dispersion was obtained. The dispersion was transferred to a 3.5 kDa dialysis membrane and dialyzed against ultrapure water containing EDTA (10 mg. L^{-1}) for the first day, followed by ultrapure water for 2 days. An aliquot was kept to structural analysis (DLS, TEM, NTA) and the rest was freeze-dried. A yellow powder was obtained with a mass yield of 84%. SEC analysis indicates M_n=9.7 Kg.mol^{-1}, $Đ$=1.2.

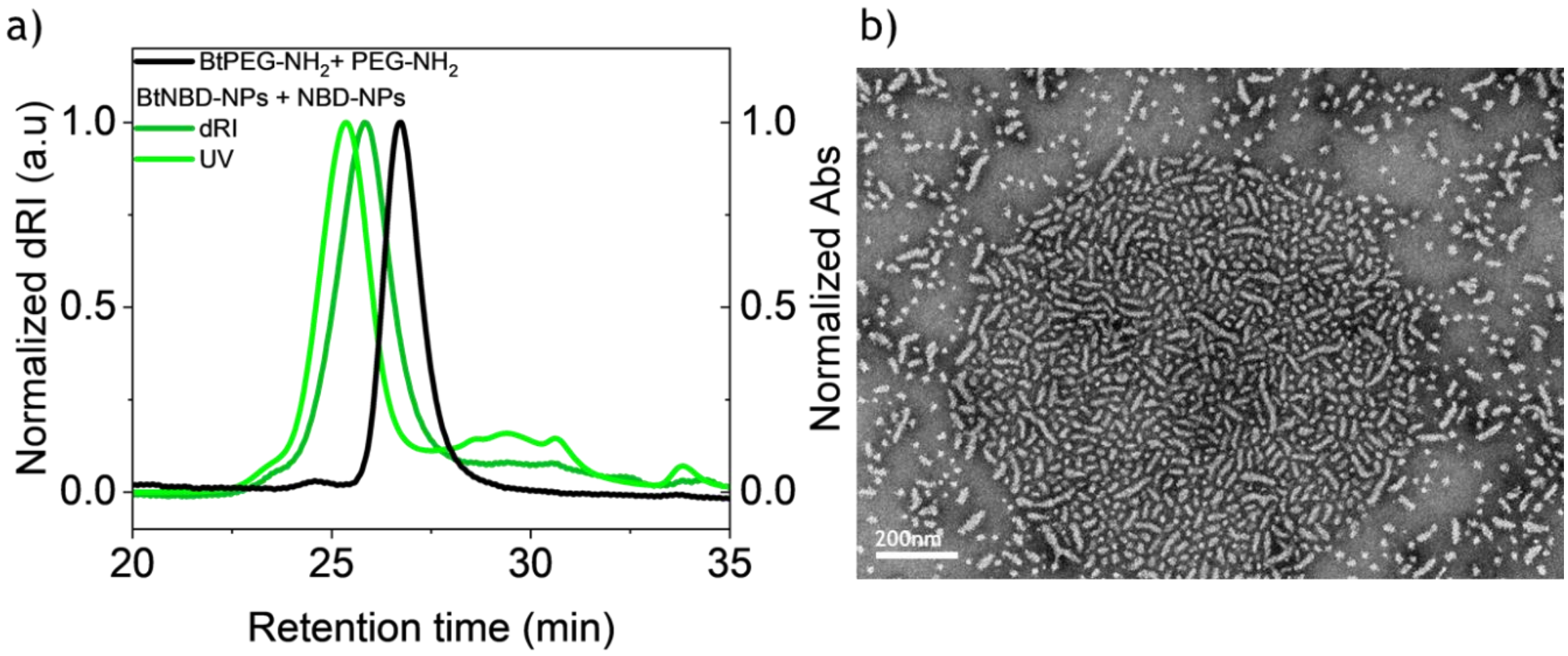

Figure SI 24: BtNBD-NPs a) Size-exclusion chromatograms in DMF (+LiBr 1mg.ml^{-1}), RI detection, UV detection. b) TEM image stained with uranyl acetate.

5. Spectra analysis

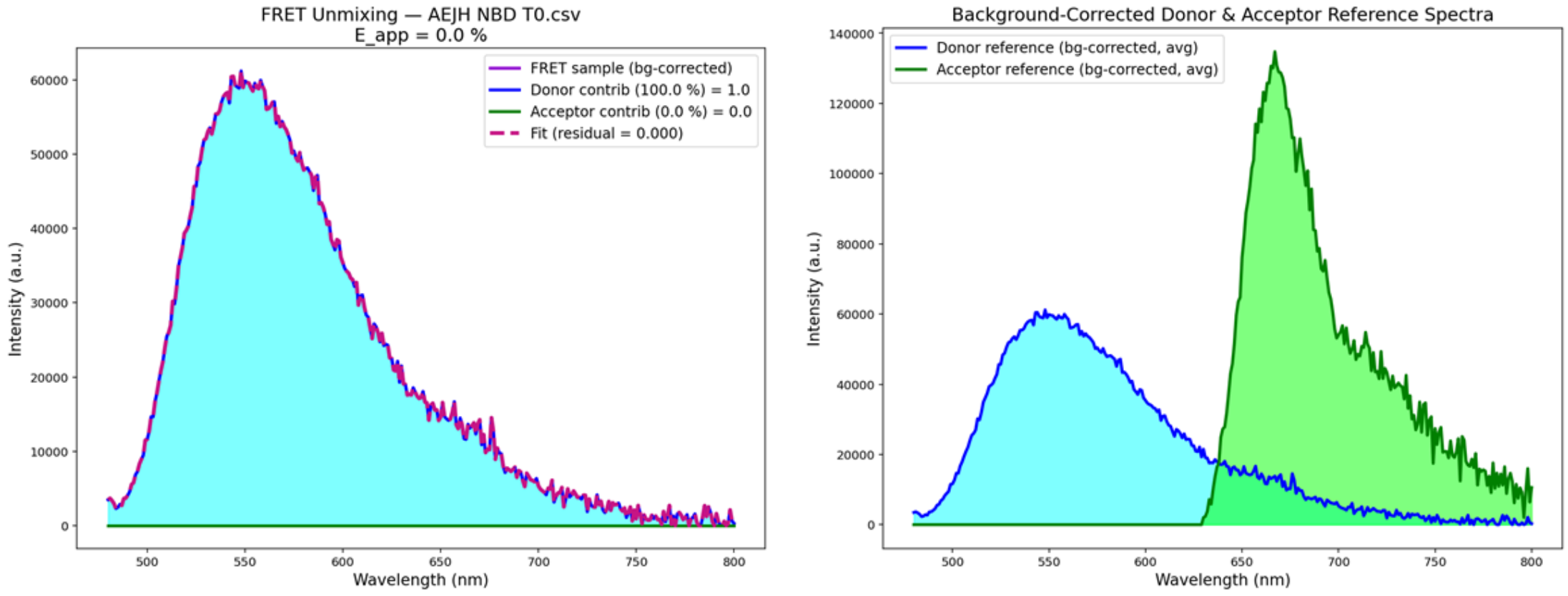

Figure SI 25: Deconvoluted fluorescence spectra of the references. Left: Fluorescence spectrum of the donor NBD at λ^{exc}=470nm. Right: Fluorescence spectrum of NBD-NPs at λ^{exc}=470nm and Cy5-NPs at λ^{exc}=630nm.

5.1. Fluorescence spectra of NBD-NPs with Cy5-NPs

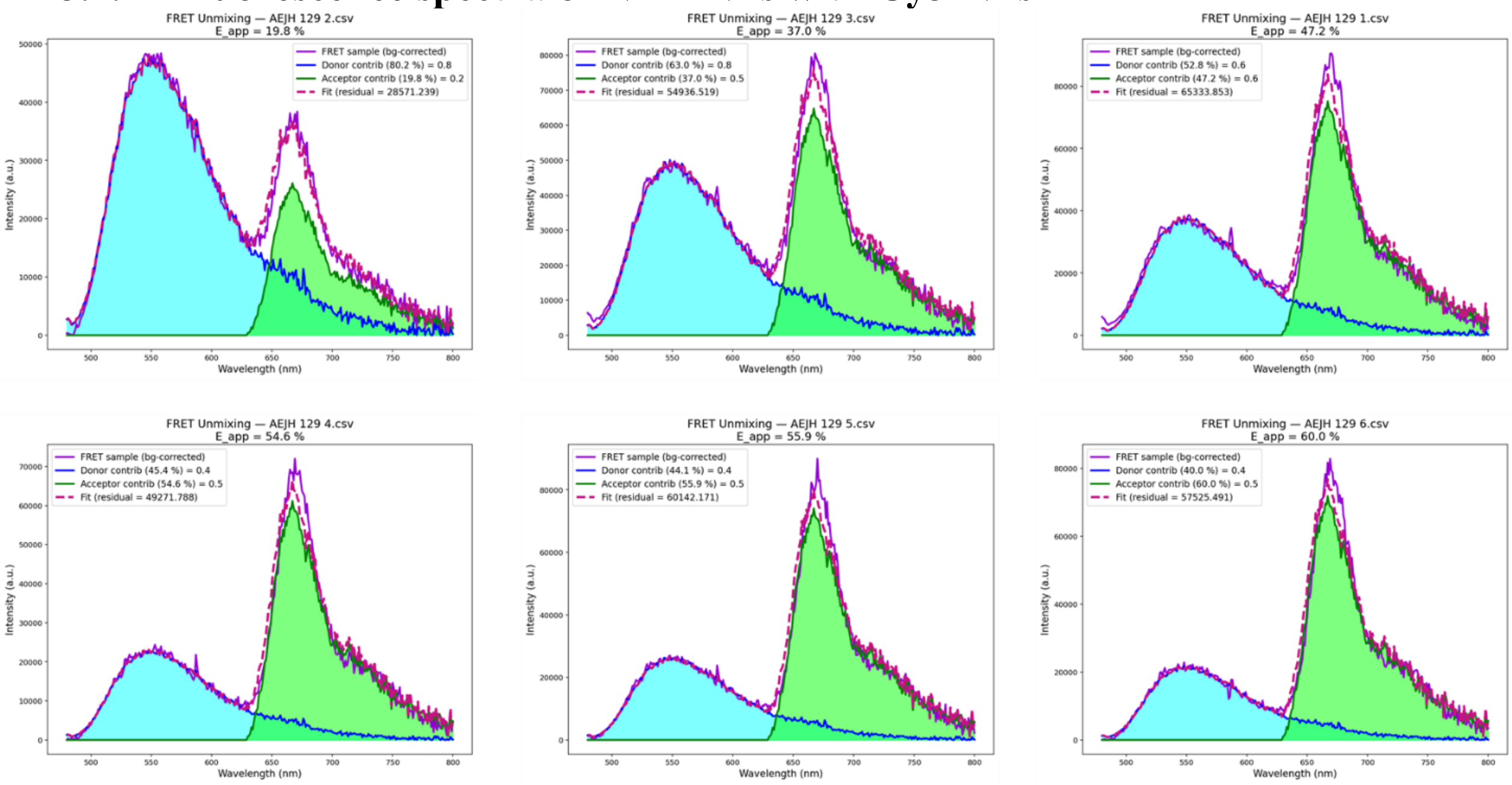

Figure SI 26: Deconvoluted fluorescence spectra of NBD-NPs mixed with Cy5-NPs at different concentrations.

5.2. Fluorescence spectra of NBD-NPs with Cy5-NPs after ultrasounds

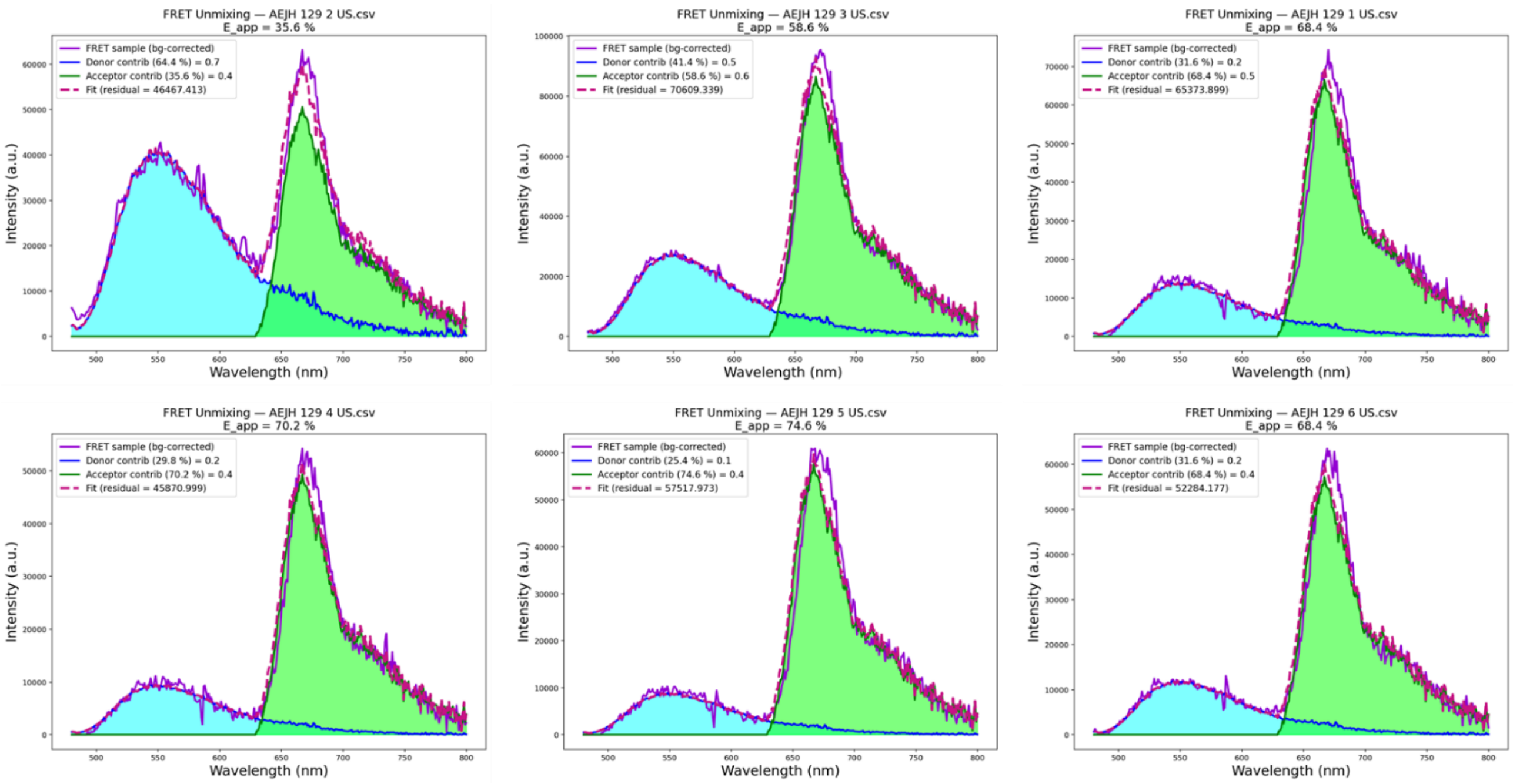

Figure SI 27: Deconvoluted fluorescence spectra of NBD-NPs mixed with Cy5-NPs at different concentrations, mixed using ultrasounds.

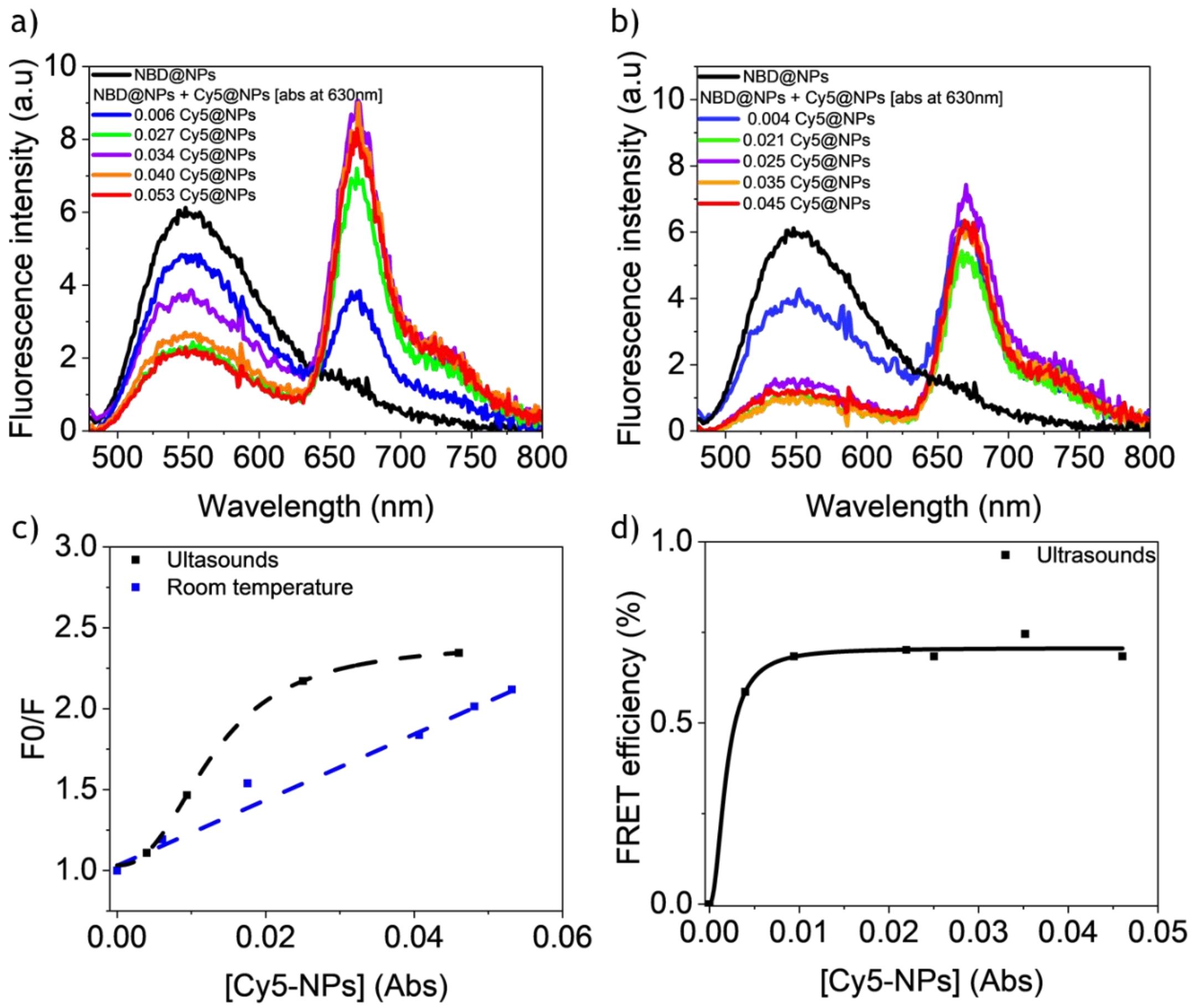

Figure SI 28: Fluorescence intensity of NBD-NPs in the presence of gradual concentrations of Cy5-NPs studied. a) At room temperature after 10 min of mixing. b) After ultrasounds. c) Stern-Volmer plot, ie fluorescence quenching of the NBD-NPs particles with gradual concentrations of Cy5-NPs at RT after 10 min, overlapped with FRET between NPs due to inter-chains exchanges (ultrasounds). Curve is fitted with a Hill equation. d) FRET efficiency after ultrasounds mixing.

5.3. Fluorescence spectra of BtNBD-NPs with SA-TR

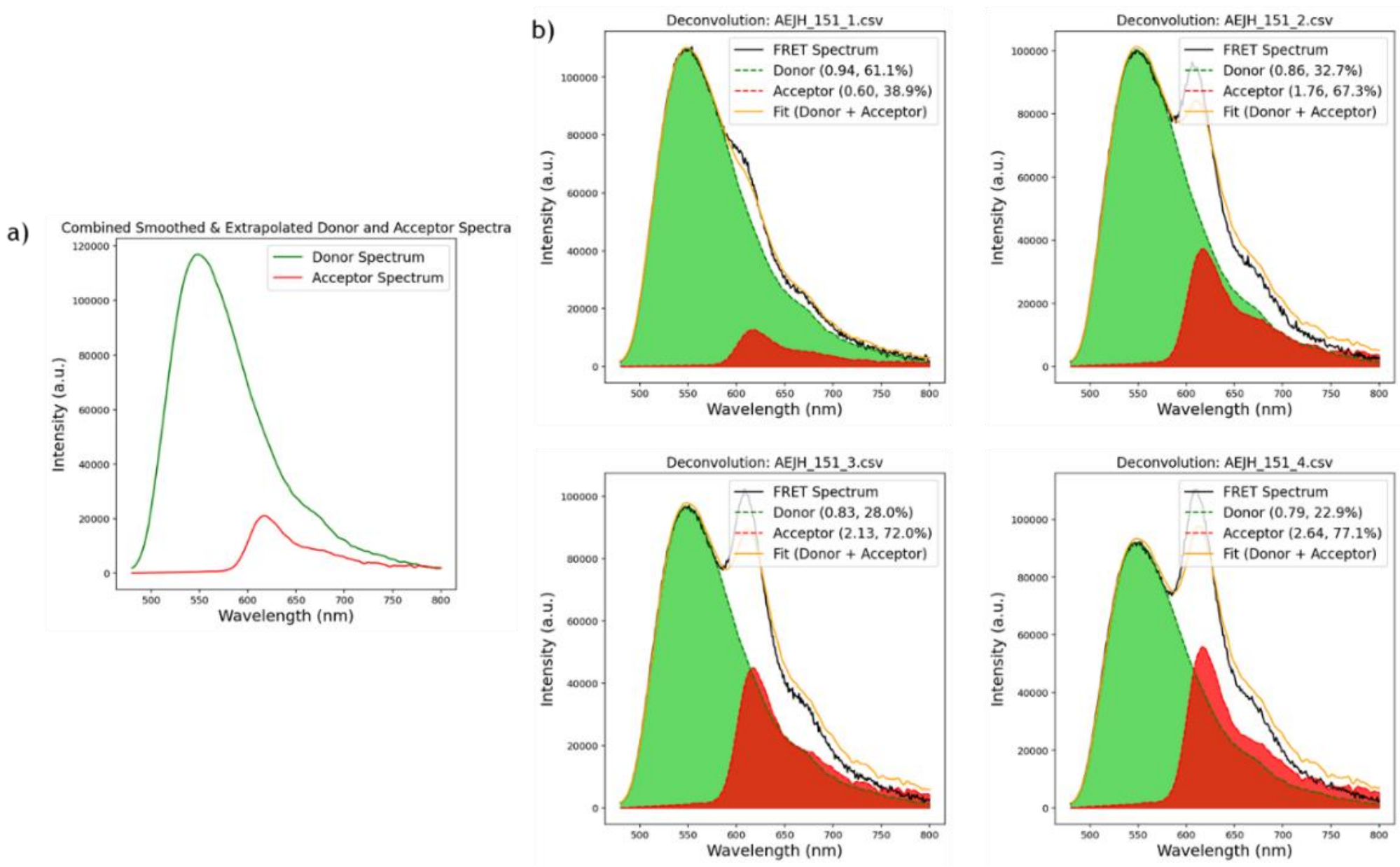

Figure SI 29: Deconvoluted fluorescence spectrum at λ^{exc}=470nm: a) References spectrum of BtNBD-NPs and SA-TR. b) Fluorescence spectrum of the mix between BtNBD-NPs and SA-TR at different concentrations.

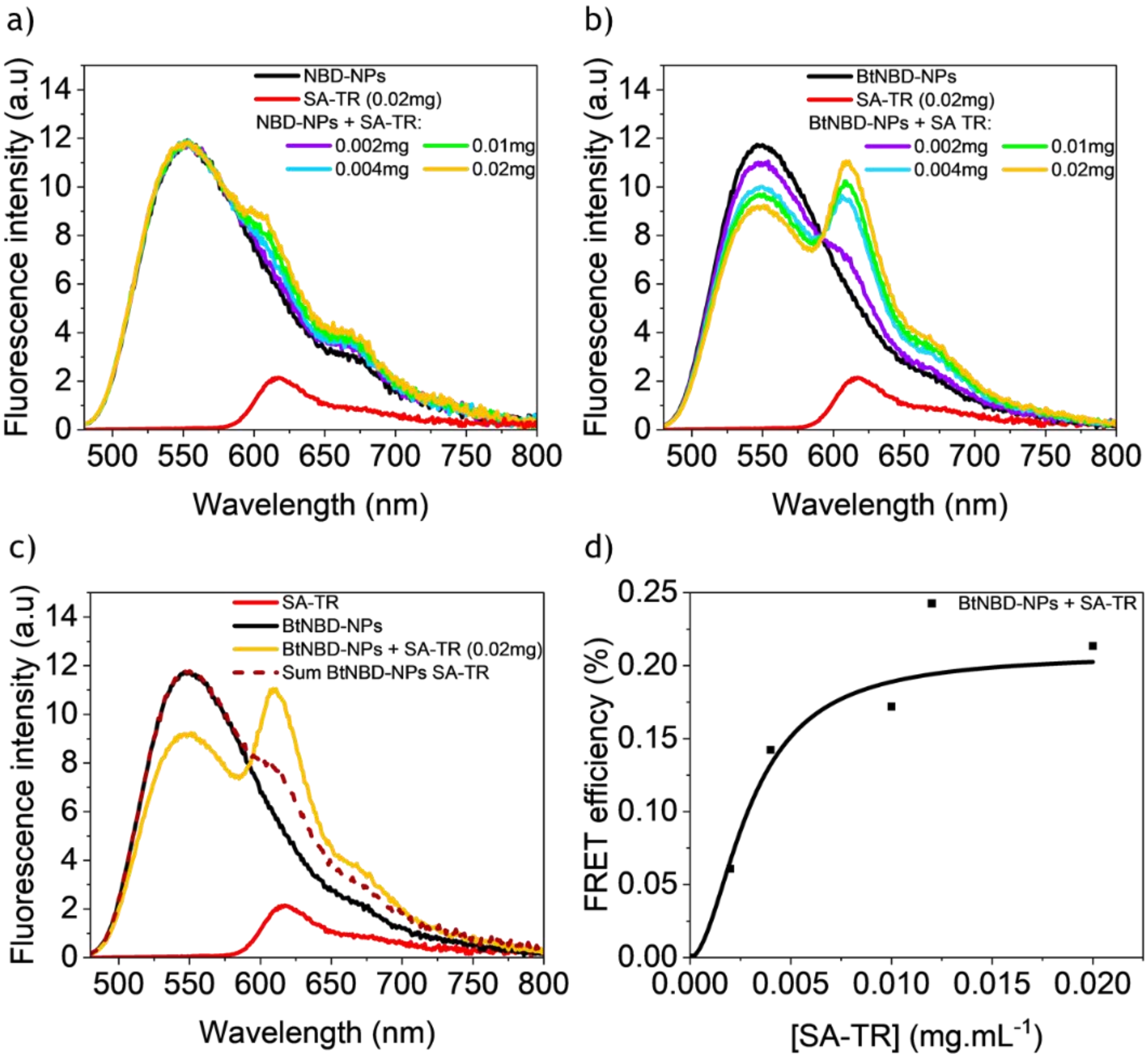

Figure SI 30: a) Fluorescence intensity of NBD-NPs at λ^{exc}=470nm mixed with SA-TR (control). b) Fluorescence intensity of BtNBD-NPs at λ^{exc}=470nm mixed with SA-TR. c) Fluorescence spectra(λ^{exc}=470nm) of btNBD-NPS, SA-TR and btNBD-NPS + SA-TR. The dotted lines correspond to the sum of btNBD-NPs spectra and SA-TR. d) FRET efficiency of BtNBD-NPs with SA-TR.

References

(1) Eaton, D. F. Reference Materials for Fluorescence Measurement. *Pure and Applied Chemistry* **1988**, *60* (7), 1107–1114. https://doi.org/10.1351/pac198860071107.

(2) Würth, C.; Grabolle, M.; Pauli, J.; Spieles, M.; Resch-Genger, U. Relative and Absolute Determination of Fluorescence Quantum Yields of Transparent Samples. *Nat Protoc* **2013**, *8* (8), 1535–1550. https://doi.org/10.1038/nprot.2013.087.

(3) Drake, J. M.; Lesiecki, M. L.; Camaioni, D. M. Photophysics and Cis-Trans Isomerization of DCM. *Chemical Physics Letters* **1985**, *113* (6), 530–534. https://doi.org/10.1016/0009-2614(85)85026-0.

(4) Sens, R.; Drexhage, K. H. Fluorescence Quantum Yield of Oxazine and Carbazine Laser Dyes. *Journal of Luminescence* **1981**, *24–25*, 709–712. https://doi.org/10.1016/0022-2313(81)90075-2.

(5) Ovesný, M.; Křížek, P.; Borkovec, J.; Švindrych, Z.; Hagen, G. M. ThunderSTORM: A Comprehensive ImageJ Plug-in for PALM and STORM Data Analysis and Super-Resolution Imaging. *Bioinformatics* **2014**, *30* (16), 2389–2390. https://doi.org/10.1093/bioinformatics/btu202.

(6) Crocker, J. C.; Grier, D. G. Methods of Digital Video Microscopy for Colloidal Studies. *Journal of Colloid and Interface Science* **1996**, *179* (1), 298–310. https://doi.org/10.1006/jcis.1996.0217.

(7) Allan, D. B.; Caswell, T.; Keim, N. C.; van der Wel, C. M.; Verweij, R. W. Soft-Matter/Trackpy: V0.7, 2025. https://doi.org/10.5281/zenodo.16089574.

(8) Zhou, Z.; Fahrni, C. J. A Fluorogenic Probe for the Copper(I)-Catalyzed Azide−Alkyne Ligation Reaction: Modulation of the Fluorescence Emission via 3(n,Π*)−(π,Π*) Inversion. *J. Am. Chem. Soc.* **2004**, *126* (29), 8862–8863. https://doi.org/10.1021/ja049684r.

(9) *Extinction Coefficient [Cy5 (Cyanine-5)] | AAT Bioquest*. https://www.aatbio.com/resources/extinction-coefficient/cy5_cyanine_5 (accessed 2026-02-02).

(10) Fischer, H.; Polikarpov, I.; Craievich, A. F. Average Protein Density Is a Molecular-Weight-Dependent Function. *Protein Science* **2004**, *13* (10), 2825–2828. https://doi.org/10.1110/ps.04688204.

(11) Engler, A. C.; Lee, H.; Hammond, P. T. Highly Efficient "Grafting onto" a Polypeptide Backbone Using Click Chemistry. *Angew Chem Int Ed* **2009**, *48* (49), 9334–9338. https://doi.org/10.1002/anie.200904070.

(12) Nguyen, M.; Stigliani, J.-L.; Bijani, C.; Verhaeghe, P.; Pratviel, G.; Bonduelle, C. Ionic Polypeptide Polymers with Unusual β-Sheet Stability. *Biomacromolecules* **2018**, *19* (10), 4068–4074. https://doi.org/10.1021/acs.biomac.8b01084.

(13) Leuchs, H. Ueber Die Glycin-Carbonsäure. *Berichte der deutschen chemischen Gesellschaft* **1906**, *39* (1), 857–861. https://doi.org/10.1002/cber.190603901133.

(14) Nguyen, T. P.; Easley, A. D.; Kang, N.; Khan, S.; Lim, S.-M.; Rezenom, Y. H.; Wang, S.; Tran, D. K.; Fan, J.; Letteri, R. A.; He, X.; Su, L.; Yu, C.-H.; Lutkenhaus, J. L.; Wooley, K. L. Polypeptide Organic Radical Batteries. *Nature* **2021**, *593* (7857), 61–66. https://doi.org/10.1038/s41586-021-03399-1.

(15) Hu, Y.; Tian, Z.-Y.; Xiong, W.; Wang, D.; Zhao, R.; Xie, Y.; Song, Y.-Q.; Zhu, J.; Lu, H. Water-Assisted and Protein-Initiated Fast and Controlled Ring-Opening Polymerization of Proline *N* -Carboxyanhydride. *National Science Review* **2022**, *9* (8), nwac033. https://doi.org/10.1093/nsr/nwac033.

(16) Sivakumar, K.; Xie, F.; Cash, B. M.; Long, S.; Barnhill, H. N.; Wang, Q. A Fluorogenic 1,3-Dipolar Cycloaddition Reaction of 3-Azidocoumarins and Acetylenes. *Org. Lett.* **2004**, *6* (24), 4603–4606. https://doi.org/10.1021/ol047955x.
